# Genomic characterization of AML with aberrations of chromosome 7: a multinational cohort of 519 patients

**DOI:** 10.1186/s13045-024-01590-1

**Published:** 2024-08-19

**Authors:** Adriane Halik, Marlon Tilgner, Patricia Silva, Natalia Estrada, Robert Altwasser, Ekaterina Jahn, Michael Heuser, Hsin-An Hou, Marta Pratcorona, Robert K. Hills, Klaus H. Metzeler, Laurene Fenwarth, Anna Dolnik, Christine Terre, Klara Kopp, Olga Blau, Martin Szyska, Friederike Christen, Jan Krönke, Loïc Vasseur, Bob Löwenberg, Jordi Esteve, Peter J. M. Valk, Matthieu Duchmann, Wen-Chien Chou, David C. Linch, Hartmut Döhner, Rosemary E. Gale, Konstanze Döhner, Lars Bullinger, Kenichi Yoshida, Frederik Damm

**Affiliations:** 1 Present Address: Department of Hematology, Oncology, and Cancer Immunology, Charité - Universitätsmedizin Berlin, Corporate Member of Freie Universität Berlin, Humboldt Universität zu Berlin, and Berlin Institute of Health, Berlin, Germany; 2 Department of Internal Medicine III, University Hospital of Ulm, Ulm, Germany; 3 Department of Hematology, Hemostasis, Oncology and Stem Cell Transplantation, Hannover Medical School, Hannover, Germany; 4 Department of Internal Medicine IV, University Hospital Halle (Saale), Martin-Luther-University Halle-Wittenberg, Halle, Germany; 5 Division of Hematology, Department of Internal Medicine, and Division of General Medicine, Department of Internal Medicine, National Taiwan University Hospital, No. 7, Chung Shan South Road, Taipei City, Taiwan; 6 Hospital de la Santa Creu i Sant Pau. Institut de Recerca Sant Pau. Department of Medicine, Universitat Autonoma of Barcelona, Barcelona, Spain; 7 Nuffield Department of Population Health, University of Oxford, Oxford, UK; 8 Department of Hematology, Cell Therapy, Hemostaseology and Infectious Diseases, University Hospital Leipzig, Leipzig, Germany; 9 Unité Mixte de Recherche (UMR) 9020–UMR1277, Canther–Cancer Heterogeneity, Plasticity and Resistance to Therapies, University of Lille, Centre National de la Recherche Scientifique (CNRS), INSERM, Centre Hospitalo-Universitaire (CHU) Lille, Institut de Recherche sur le Cancer de Lille (IRCL), Lille, France; 10 Laboratoire de Cytogénétique, Service de Biologie, CH de Versailles, Le Chesnay, France; 11 Hematology Department, Saint Louis Hospital, AP-HP, Paris, France; 12 Department of Hematology, Erasmus MC Cancer Institute, and Erasmus University Medical Center Rotterdam, Rotterdam, The Netherlands; 13 Hematology Department, IDIBAPS, Hospital Clínic de Barcelona, University of Barcelona, Barcelona, Spain; 14 Institut de Recherche Saint-Louis (IRSL), Institut National de la Santé et de la Recherche Médicale (INSERM) U944, Centre National de la Recherche Scientifique (CNRS) UMR 7212 GenCellDis, Université Paris Cité, Paris, France; 15 Department of Haematology, University College London Cancer Institute, London, UK; 16 German Cancer Consortium (Deutsches Konsortium Für Translationale Krebsforschung, DKTK), Partner Site, Berlin, Germany; 17 Division of Cancer Evolution, National Cancer Center Research Institute, Tokyo, Japan

**Keywords:** AML, del(7q), Monosomy 7, Complex karyotype, *KMT2C*, *TP53*, *IDH2*, *PTPN11*

## Abstract

**Background:**

Deletions and partial losses of chromosome 7 (chr7) are frequent in acute myeloid leukemia (AML) and are linked to dismal outcome. However, the genomic landscape and prognostic impact of concomitant genetic aberrations remain incompletely understood.

**Methods:**

To discover genetic lesions in adult AML patients with aberrations of chromosome 7 [abn(7)], 60 paired diagnostic/remission samples were investigated by whole-exome sequencing in the exploration cohort. Subsequently, a gene panel including 66 genes and a SNP backbone for copy-number variation detection was designed and applied to the remaining samples of the validation cohort. In total, 519 patients were investigated, of which 415 received intensive induction treatment, typically containing a combination of cytarabine and anthracyclines.

**Results:**

In the exploration cohort, the most frequently mutated gene was *TP53* (33%), followed by epigenetic regulators (*DNMT3A*, *KMT2C, IDH2*) and signaling genes (*NRAS*, *PTPN11*). Thirty percent of 519 patients harbored ≥ 1 mutation in genes located in commonly deleted regions of chr7—most frequently affecting *KMT2C* (16%) and *EZH2* (10%). *KMT2C* mutations were often subclonal and enriched in patients with del(7q), de novo or core-binding factor AML (45%). Cancer cell fraction analysis and reconstruction of mutation acquisition identified *TP53* mutations as mainly disease-initiating events, while del(7q) or −7 appeared as subclonal events in one-third of cases. Multivariable analysis identified five genetic lesions with significant prognostic impact in intensively treated AML patients with abn(7). Mutations in *TP53* and *PTPN11* (11%) showed the strongest association with worse overall survival (OS, *TP53*: hazard ratio [HR], 2.53 [95% CI 1.66–3.86]; *P* < 0.001; *PTPN11*: HR, 2.24 [95% CI 1.56–3.22]; *P* < 0.001) and relapse-free survival (RFS, *TP53*: HR, 2.3 [95% CI 1.25–4.26]; *P* = 0.008; *PTPN11*: HR, 2.32 [95% CI 1.33–4.04]; *P* = 0.003). By contrast, *IDH2*-mutated patients (9%) displayed prolonged OS (HR, 0.51 [95% CI 0.30–0.88]; *P* = 0.0015) and durable responses (RFS: HR, 0.5 [95% CI 0.26–0.96]; *P* = 0.036).

**Conclusion:**

This work unraveled formerly underestimated genetic lesions and provides a comprehensive overview of the spectrum of recurrent gene mutations and their clinical relevance in AML with abn(7). *KMT2C* mutations are among the most frequent gene mutations in this heterogeneous AML subgroup and warrant further functional investigation.

**Supplementary Information:**

The online version contains supplementary material available at 10.1186/s13045-024-01590-1.

## Introduction

Acute myeloid leukemia (AML) is characterized by aberrant proliferation of hematopoietic progenitor cells in the bone marrow, leading to suppression of normal hematopoiesis [1]. Chromosomal aberrations and gene mutations play a pivotal role during leukemogenesis and are essential for risk stratification and personalized treatment approaches [2, 3]. Aberrations of chromosome 7 [abn(7)], for which the DNA sequence and annotation were only described two decades ago[4], are common and found in ~ 10% of newly diagnosed AML [5, 6]. The most common abn(7) include complete loss of chromosome 7 [monosomy 7, (−7)] and deletions of the long arm of chromosome 7 [del(7q)] [7]. Both frequently appear in the context of a complex karyotype (CK) [6, 8], which is an established poor prognostic factor [2, 3]. Outside a CK, the current 2022 ELN risk classification assigns only −7 to the adverse risk category, while del(7q), in the absence of additional good or adverse genetic markers, belongs to the intermediate risk group [3]. With respect to co-occurring gene mutations, only a few studies have systematically investigated the landscape and clinical impact of concomitant aberrations in AML with abn(7) [9–12]. However, *TP53* mutations frequently occur in AML with abn(7), especially in cases with CK, and are associated with dismal prognosis [13, 14]. *TP53*-status has been recently included as a new disease category in the International Consensus Classification of Myeloid Neoplasms and Acute Leukemias (ICC) [15].

Four commonly deleted regions (CDR) in chromosome 7 (chr7) have been reported: 7q21.2, 7q22.1, 7q34, and 7q35-36.1 [16]. These regions encode for several genes with a well-established functional role in hematopoiesis and are recurrent targets of somatic mutations in various hematologic malignancies such as *EZH2, BRAF,* or *SAMD9* [17–22]. For example, mutations affecting *EZH2* have been reported in up to 30% of follicular lymphoma and 5–10% of myelodysplastic syndromes, chronic myelomonocytic leukemia, and AML [2, 18, 23–25]. Also, deregulated mRNA expression of genes within these CDRs has been aligned with patient outcome, e.g., altered expression of the methyltransferase *MLL5/KMT2E*, located on chr7q22.3, associates with poor prognosis [26].

To date, studies focusing on AML with abn(7) were showing limitations regarding cohort size, sequencing technologies applied, and cohort heterogeneity, often investigating a wide variety of myeloid neoplasms with abn(7) precluding generalization for AML [9, 10, 12].

Therefore, we embarked on a comprehensive study to decipher the genomic landscape of AML with abn(7) in a large international cohort of 519 adult AML patients using a combination of whole-exome (WES) and targeted sequencing (TS). This workflow enabled the detection of previously underestimated somatic mutations and copy number variations (CNV) and allowed for the identification of genetic markers important for further risk refinement in this specific AML subgroup.

## Methods

### Patients

**Fig. 1:**
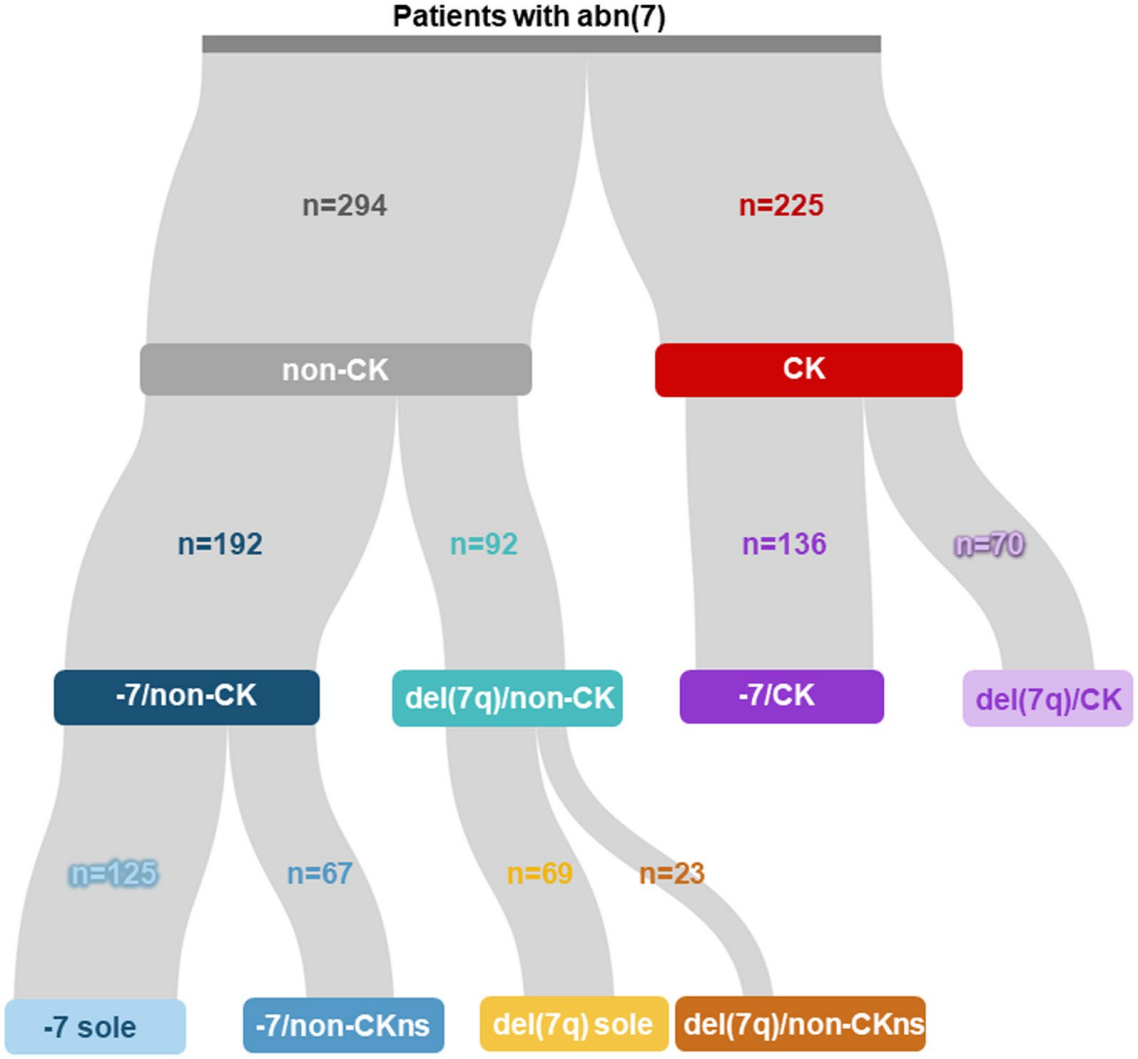
Classification of the abn(7) cohort according to cytogenetic characteristics. Depiction of the classification of abn(7) patients into different groups according to the karyotype information available (n = 519). The first level separates a group of abn(7) samples with complex karyotype (CK) from the non-CK cases. A second division separates non-CK cases and CK cases according to the presence of monosomy 7 (−7/non-CK or −7/CK) or the presence of a del(7q) [del(7q)/non-CK or del(7q)/CK]. A group of samples not fitting these definitions was not included (other/non-CK, n = 10 or other/CK, n = 19). A third division separates samples by the presence or absence of another cytogenetic alteration in addition to the abnormality detected in abn(7)/non-CK. Samples with an accompanying alteration (non-complex karyotype non-sole, non-CKns) and samples with only a chr7 aberration (sole)

A total of 519 samples (bone marrow or peripheral blood) from first diagnosis of adult AML patients with abn(7) were collected from collaborating study groups in France, Germany, the Netherlands, Spain, Taiwan, and the United Kingdom. Patients had a median age of 59 years (range 15–89 years). For 60 patients, complete remission (CR) samples were used as non-tumor controls. Baseline karyotype information was obtained through conventional karyotyping at diagnosis. 225 patients (43%) had a chr7 aberration in the context of a CK (Fig. 1). Among the 294 patients without a CK (non-CK), 125 patients showed a −7 (−7sole) and 69 patients a deletion of 7q as sole aberrations [del(7q)sole]. Eighty percent of patients (n = 415) received intensive induction treatment, typically containing a combination of cytarabine and anthracyclines (“7 + 3”). Fifty-three percent of patients were treated within prospective trials of participating study groups. Furthermore, 33% of patients underwent allogeneic hematopoietic stem cell transplantation (allo-HSCT) in first CR (CR1, n = 124) or as salvage (n = 44) therapy. The characteristics and therapy specifications of patients are shown in Table 1 and supplemental Dataset 1. Written consent was obtained from all patients according to the Declaration of Helsinki, and the local ethics committee granted ethical approval.

**Table 1:** Patient characteristics of the abn(7) cohort

Baseline patient characteristics	All patients, n = 519	Intensively treated patients, n = 415
Sex		
Female	233 (45%)	189 (46%)
Male	286 (55%)	226 (54%)
Age (years)		
Median (IQR)	59 (47, 69)	55 (44, 64)
AML type		
De novo	404 (78%)	336 (81%)
Secondary	75 (14%)	47 (11%)
Therapy-related	39 (7%)	31 (7%)
Missing data	*1 (*< *1%)*	*1 (*< *1%)*
ELN risk category [3]		
Favorable	11 (2%)	11 (3%)
Intermediate	31 (6%)	24 (6%)
Adverse	475 (92%)	378 (91%)
Missing data	*2 (*< *1%)*	*2 (*< *1%)*
WBC at diagnosis (/nL)		
Median (IQR)	9 (3, 30)	9 (3, 30)
Missing data	*14 (3%)*	*9 (2%)*
Blast count at diagnosis (%)		
Median (IQR)	55 (33, 80)	58 (37, 82)
Missing data	*46 (9%)*	*31 (7%)*
Platelets at diagnosis (/nL)		
Median (IQR)	47 (24, 96)	52 (24, 102)
Missing data	*49 (9%)*	*43 (10%)*
Hemoglobin at diagnosis (g/dL)		
Median (IQR)	8.5 (7.15, 9.7)	8.5 (7.1, 9.9)
Missing data	*108 (21%)*	*102 (24%)*
Intensive treatment		
Yes	415 (80%)	415 (100%)
No	100 (19%)	–
Missing data	*4 (1%)*	–
Complete remission		
Yes	268 (52%)	253 (61%)
No	249 (48%)	160 (39%)
Missing data	*2 (*< *1%)*	*2 (*< *1%)*
Allo-HSCT		
In CR1	126 (24%)	123 (30%)
Non-CR1/Salvage	38 (7%)	34 (8%)
Unknown timepoint	6 (1%)	6 (1%)
No	301 (58%)	208 (50%)
Missing data	*48 (9%)*	*44 (11%)*
Treatment within trial		
Yes	276 (53%)	275 (66%)
No	239 (46%)	139 (34%)
Missing data	*4 (1%)*	*1 (*< *1%)*

Baseline characteristics of the whole (n = 519) and intensively treated (n = 415) abn(7) patient cohort. Continuous variables are given with a median (interquartile range, IQR), and discrete variables are provided with no. (%). CR1 = first complete remission

Italic values indicate missing data

### Whole-exome sequencing in the abn(7) exploration cohort

#### Analysis of single nucleotide variations, mutation signatures, and copy number variations from WES

Paired first diagnosis/CR samples were available from 60 patients for explorative mutation analysis through WES. Samples were prepared using the SureSelect XT-HS exome and SureSelect Human All Exon v7 library preparation kits (Agilent Technologies, Santa Clara, CA, USA). Sequencing was performed at the Genomic Core facility of the Berlin Institute of Health (BIH) on the NovaSeq 6000 platform (Illumina, San Diego, CA) with a median coverage of 211-fold. We used a detection cut-off for variant allele frequency (VAF) of ≥ 5%. Called variants were filtered thoroughly as described previously [27–29]. Analysis of mutational signatures and variant filtering are specified in the supplemental methods.

### Targeted sequencing in the abn(7) extension cohort

#### Panel design and analysis of single nucleotide variations using TS

Based on our WES-results, literature research, and data from additional AML cases with abn(7) from the Cancer Genome Atlas database (TCGA) [2], we designed a unique gene panel covering 66 genes (Table S1) and genomic regions frequently affected by CNVs. A single nucleotide polymorphism (SNP)-backbone of 1217 probes was included to identify CNVs (supplemental dataset 2).

TS was performed on 467 diagnostic samples (= extension cohort) using a customized Twist Bioscience Gene Panel (Twist Bioscience, San Francisco, CA, USA). Samples were prepared with the Twist Bioscience Custom Prep Kits with unique molecular identifiers for error correction following the manufacturer's protocol. Sequencing was performed using a NovaSeq 6000-Sequencer (Illumina, San Diego, CA, USA) at the BIH Core Facility, with a mean coverage of 532-fold. Variants were called using an in-house pipeline [27] with a VAF cut-off at ≥ 2% after rigorous filtering criteria for quality and clinical scores (supplemental Methods, Figure S1).

#### Analysis of copy number variations from TS

Data obtained from the SNP-backbone was used to estimate copy number (CN) and B-allele-frequencies (BAF) for CNV and copy-neutral loss of heterozygosity (cnLOH) determination using the *PureCN* R-package [30]. For correction of assay-specific capture biases, we used 19 pooled healthy control samples. We restricted the analysis to samples with a ploidy between 1.5–3N. An alteration was considered a gain for log(CN) > 0.4 and a deletion with log(CN) < −  0.3. Upon curation, 342 samples passed our quality criteria, ensuring high reproducibility of the presented data. Manual curation was done to merge continuous segments and to rule out disagreements with BAF. We pursued internal validations (supplemental Methods) by calculation of CNV agreements with G-Banding information and with matching samples sequenced by long-read Oxford Nanopore Technology (n = 33, ONTseq, as previously described) [31] along with comparisons with WES-CNV-results.

### Cancer cell fraction analysis and Bradley–Terry model

To unravel clonal hierarchies, we calculated cancer cell fractions (CCF) for mutations and CNVs from WES data as previously described [32]. To investigate the timing and order of mutation acquisition, we used similar calculations of CCFs [33] based on the *PureCN* algorithm data for the extension cohort. We applied a Bradley–Terry Model with pairwise comparison of the highest CCFs of a gene-sample pair for samples with available single nucleotide variant (SNV)/CNV/cnLOH data as previously reported[27] (supplemental Methods).

### Statistical and survival analysis

All statistical analyses were performed using R statistical software packages (supplemental Methods). For survival analyses, only intensively treated patients were considered (n = 415) to ensure comparability and avoid potential treatment bias. The definitions of CR, overall survival (OS), and relapse-free survival (RFS) followed the recommended criteria [3]. Kaplan–Meier analysis was used to create survival curves. Log-rank tests were applied to evaluate differences between groups and were considered statistically significant if *P* < 0.05. Univariate and multivariable Cox proportional hazards models were used to investigate the association of variables for OS and RFS. The Cox proportionality assumption was guaranteed for all reported results.

## Results

### The genomic landscape of AML with abn(7)

**Fig. 2:**
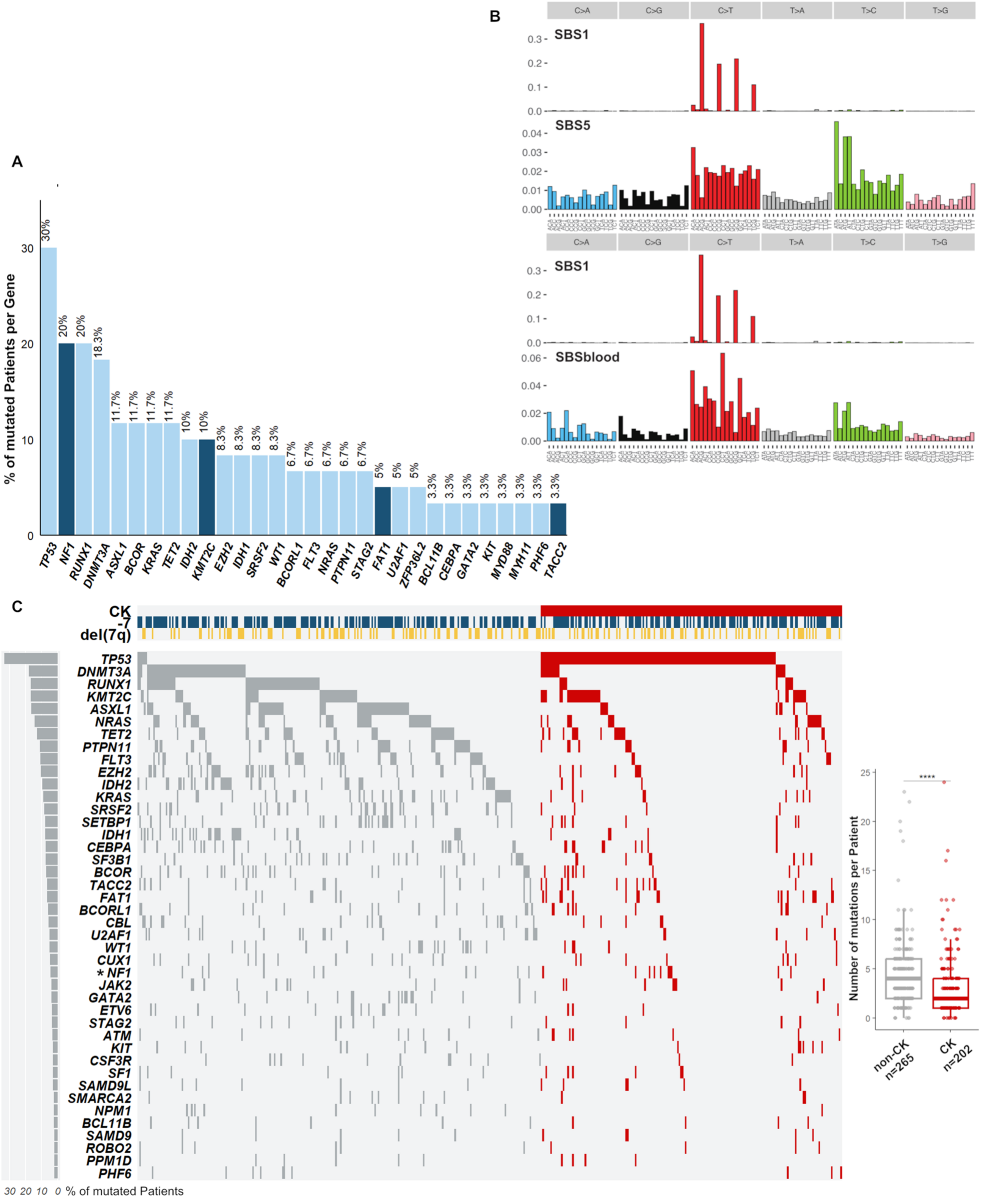
Mutations and SBS signatures found by WES and Targeted Sequencing in the abn(7) exploration and extension cohorts. **A** Bar graph showing the frequency of mutations identified by WES in patients (n = 60) per gene for genes mutated in ≥ 2 patients. Bars colored dark blue signify genes of particular importance due to high mutation frequency or previously underestimated prevalence in AML. The fraction of mutated patients per gene is shown above each bar (%). **B** The graph shows the mutational profile extracted from WES, which represents the two main signatures of most samples from the exploration cohort. Sig-A had high cosine similarities to the signatures reconstituted from the components that expectation maximization extracted SBS1/SBS5 in COSMIC (0.939) and SBS1/SBSblood in normal blood cells (0.945)[34, 35]. The bars represent the relative contributions of clustered genome-wide substitutions (SBS, y-axis) for each 96 trinucleotide sequences (x-axis) and distributed across the six possible cytosine or thymine bases substitutions. **C** Oncoplot showing mutations found in 43 of the 64 genes included in the TS gene panel in 452 patients of the extension cohort (n = 467 patients). According to baseline karyotype information, patients were segregated into two major groups: abn(7)/non-complex karyotype (non-CK, grey) or abn(7)/complex karyotype (CK, red). The marks on the top rows correspond to patients classified by cytogenetic information into having −7 (dark blue) or del(7q) (yellow). *FLT3* includes all *FLT3* alterations. *In the TS panel, only exons 28–38 of *NF1* were investigated. The left bar plot shows the frequency (%) of mutated patients per gene. On the right side, a boxplot shows the statistical difference between the number of mutations found for these groups, median 4 versus 2 respectively for abn(7)/non-CK versus abn(7)/CK (two-tailed *t* test, n = 467, ****P < 0.0001)

Explorative WES of 60 paired diagnostic/CR samples revealed a total of 932 SNVs in 742 individual genes (median 12 SNVs/patient, supplemental dataset 3). Among these, we found 207 mutations in 59 genes known to be recurrently mutated in AML with a median of 2 mutated genes per patient. The most frequently mutated gene was *TP53* (30%), followed by *NF1* (20%), *RUNX1* (20%), and *DNMT3A* (18.3%; Fig. 2A). Genes involved in epigenetic regulation were mutated in 58.3% of our exploration cohort and included *DNMT3A* (18.3%), *ASXL1* (11.7%), *TET2* (11.7%), *IDH2* (10%), *KMT2C* (10%), *EZH2* (8.3%), and *IDH1* (8.3%, Fig. 2A). Of note, belonging to the group of genes located in the CDRs of chr7, *KMT2C,* and *EZH2* showed a high mutation frequency.

To dissect biological processes that result in the accumulation of somatic alterations during leukemogenesis, we investigated mutational signatures in our exploration cohort. One signature was extracted (Sig-A), which had high cosine similarities to SBS1/SBS5 in COSMIC (0.939) and SBS1/SBSblood in normal blood cells (0.945, Fig. 2B). The SBS1 and SBS5 (SBSblood) are age-related endogenous mutational signatures and have been reported as the predominant signatures in AML and myeloproliferative neoplasms [34] as well as in normal hematopoietic stem and progenitor cells [35].

**Fig. 3:**
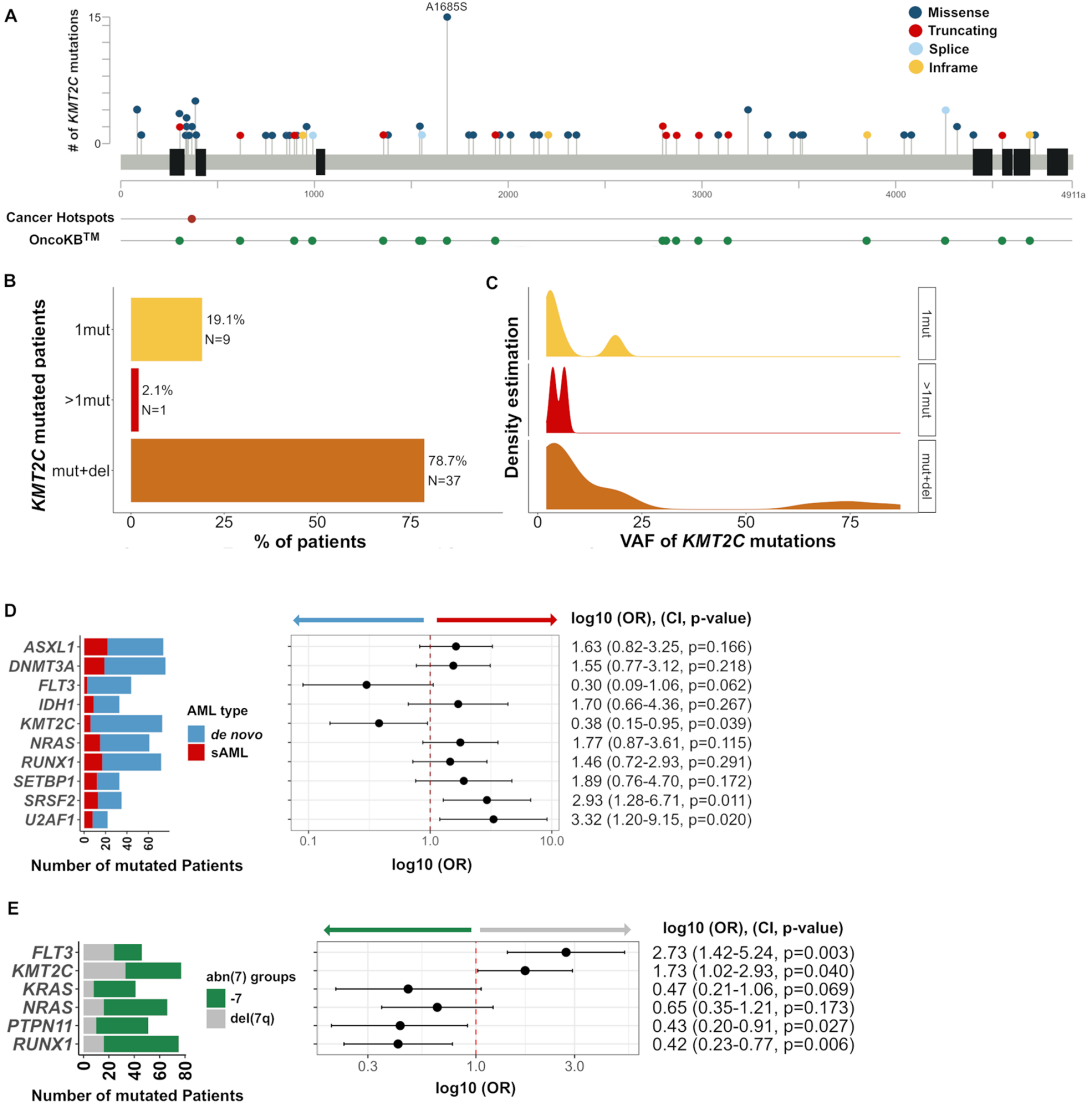
Frequent low allele-burden mutations in *KMT2C* in AML with abn(7). **A** Lollipop plot depicts the localization and frequency of each *KMT2C* variant (n = 98, in 78/467 patients, from *KMT2C* refSeq NM_170606.3). Distribution in the context of Pfam domains was adapted from MutationMapper from cbioportal [64, 65] with information on the overlap of mutations to a statistically significant hotspot in cancer [66] or to reports of functional effects in the oncology knowledge base OncoKB™ [67, 68]. Color codes were used to distinguish types of observed mutations: 72 missense (blue), 16 truncating (red), 4 inframe (yellow), and 6 alterations affecting splice sites (light blue). **B** Frequency distribution of genomic events affecting the *KMT2C* locus: SNVs and CNVs lead to a multi-hit classification of 47 *KMT2C*-mutated patients from the extension cohort (n = 342) into groups according to genomic events present in the locus (1mut, > 1mut and mut + del). **C** Distribution of the VAF values of *KMT2C* mutations found for patients in the *KMT2C* multi-hit groups with at least one mutation present (n = 47). **D**, **E** The Odds ratio plot shows a multivariable binomial logistic regression fitted for the (**D**) ten genes with a *P* < 0.1 in univariate analysis (Figure S4A) and for (**E**) the six genes with a *P* < 0.1 in univariate analysis (Figure S4B). To the left, a bar plot diagram depicts the number of mutated patients: for each of the ten genes included in the multivariate model color-coded by (**D**) type of AML (de novo AML, blue; sAML, red) and (**E**) for each of the six genes included in the multivariate model color-coded by abn(7) group [−7, green; del(7q), grey]. To the right, logOR with confidence intervals of 95%, CI, and *P* values are shown

Using TS in the extension cohort of 467 AML samples, we identified 1821 SNVs in 64 genes (median 3 SNVs/patient; Figure S2A, supplemental dataset 4). At least one gene mutation was detected in 452/467 investigated samples (96.7%). Consistent with our WES results, the most frequently mutated gene was *TP53* (33.4%), followed by *DNMT3A* (18%), *RUNX1* (16.7%), *KMT2C* (16.7%), *ASXL1* (16.3%), *NRAS* (14.3%), *TET2* (12.8%), *PTPN11* (11.1%), *EZH2* (10.3%) and *IDH2* (9.4%) (Fig. 2C, Figure S2B). Mutations affecting genes of the splicing machinery (e.g. *SRSF2* and *U2AF1)* were found in 25% of patients, while CHIP (clonal hematopoiesis of indeterminate potential)-associated gene mutations in *ASXL1*, *DNMT3A,* and *TET2* [36, 37] were found in 40% of patients and associated with older age, as expected (*P *< 0.001). Of note, the high prevalence of *KMT2C* mutations found by WES was confirmed by TS (16.7%). In 78 patients a total of 98 *KMT2C* mutations were found with a median VAF of 5% (Fig. 3A, Figure S3A). 27% of mutations were indel or splice site alterations and spread over the entire coding region, while missense mutations showed a hotspot affecting codon A1685 (n = 15). This particular mutation (COSV51390875) has been mainly reported as somatic in various cancer entities, including non-Hodgkin lymphoma [22, 38, 39]. Although we could not independently confirm the somatic origin due to lack of non-tumor tissue, the observed subclonality of *KMT2C* A1685S mutations made a germline origin unlikely (Figure S3B). Of note, this particular *KMT2C* variant was not detected in the exploration cohort. 79% of the *KMT2C*-mutated patients harbored a concomitant deletion in the *KMT2C* locus (Fig. 3B). *KMT2C* mutations were enriched in patients with core-binding factor (CBF) AML [45%, inv(16): n = 3/8, and t(8;21): n = 2/3] but not in patients with a inv(3)/t(3;3) [14.8%, 4/27, Table S2]. *KMT2C*-mutated patients were significantly associated with de novo AML (*P* = 0.039), whereas mutations in splicing factor genes, *SRSF2* and *U2AF1*, were significantly enriched in secondary AML (sAML; respectively *P* = 0.011 and *P* = 0.02 in the multivariate analysis, Fig. 3D, Figure S4A). Furthermore, we found both *KMT2C* and *FLT3* mutations to be significantly enriched in del(7q) patients (*P* = 0.04 and *P* = 0.003 respectively, Fig. 3E), while patients with −7 were enriched for *RUNX1* and *PTPN11* mutations (*P* = 0.006 and *P* = 0.027 respectively; Fig. 3E, Figure S4B). A similar association was also observed for *FLT3* and *KMT2C* mutations when comparing del(7q)/CK to –7/CK patients (*P* = 0.052 and *P* = 0.065 respectively, Figure S5).

Additionally, we identified unexpected recurrent mutations in *SETBP1* (7.7%), *FAT1,* and *TACC2* (6.4% each), and a considerable low frequency of *NPM1* (2.4%), the latter usually occurring in roughly 30% of adult AML cases [2]. TACC2 belongs to a conserved family of centrosome- and microtubule-interacting proteins and has been reported as a putative tumor suppressor in breast cancer [40, 41].

A significantly lower number of mutations was found in patients with abn(7)/CK as compared to abn(7) patients without CK (non-CK; median number of mutations/patient 2 vs. 4, *P* < 0.0001, Fig. 2C). We noted significant enrichment of *TP53* and *FAT1* mutations in patients with CK, while mutations affecting *IDH1, IDH2*, *CBL*, and *RUNX1* were predominantly found in non-CK cases (Figure S6). 30% of abn(7) patients (n = 140) harbored at least one mutation in genes located within the CDRs of 7q, most frequently in *KMT2C* (16.7%), *EZH2* (10.3%), and *CUX1* (4.7%, Figure S7A). While the highest frequencies of *EZH2* mutations were observed in patients with non-CK, *KMT2C* mutations were particularly enriched in del(7q)/CK patients (Figure S7B).

Within the non-CK group, we identified specific mutational patterns for −7 and del(7q). We found higher frequencies of *KRAS* and *RUNX1* mutations in −7/non-CK patients and more frequent *FLT3*-mutations in del(7q)/non-CK cases (Figure S8). These mutational patterns were also confirmed in patients with −7 and del(7q) as sole aberrations (Figure S9). Next, we searched for pairwise gene associations to identify patterns of mutation co-occurrence or mutual exclusivity. We confirmed several significant co-occurrences reported in other AML subgroups and related myeloid malignancies, such as *DNMT3A*/*IDH1* and *BCOR/BCORL1* [2, 42–44]. With respect to mutual-exclusivity, *TP53* mutations occurred exclusive of most other known AML mutations (Figure S10). Furthermore, exclusivities for *SRSF2/EZH2* and previously reported *TET2/IDH1/2* [45] were found. We noted a trend for mutual exclusivity for *KMT2C* mutations with *JAK2* (*P* = 0.008, FDR = 0.042), *ASXL1* (*P* = 0.009, FDR = 0.046) and *DNMT3A* mutations (*P* = 0.029, FDR = 0.12).

These data collectively indicate that distinct mutation profiles can be observed in patients with abn(7) and depend on both type of deletion (complete versus partial) and concurrent cytogenetic aberrations.

### Concomitant copy number variations in AML patients with abn(7)

Based on conventional karyotyping techniques (e.g., G-banding, FISH probing), 63% of the cohort had −7 and 32% del(7q). The remaining patients (5%) had various chr7 aberrations [e.g., iso(7p), r(7), add(7)]. Many additional cytogenetic aberrations were reported. The five most common were deletions affecting chr5 [−5/del(5q) = 28%], chr17 [−17/del(17p)/del(17q) = 14.6%], chr18 [−18/del(18p)/del(18q) = 10.2%], chr16 [−16/del(16q) = 8.1%], and gains of chr8 [+ 8/add(8q) = 9.8%].

In the extension cohort, high-quality CNV data was generated for 342 of 467 patients (73%, supplemental datasets 5,6). TS-based CNV analysis revealed a high concordance with WES-CNV data and with the five most frequent aberrations detected by conventional karyotyping, assuming the standard limit of detection for G-banding of > 10 Mb (Table S3, Figure S11-12A,B).

**Fig. 4:**
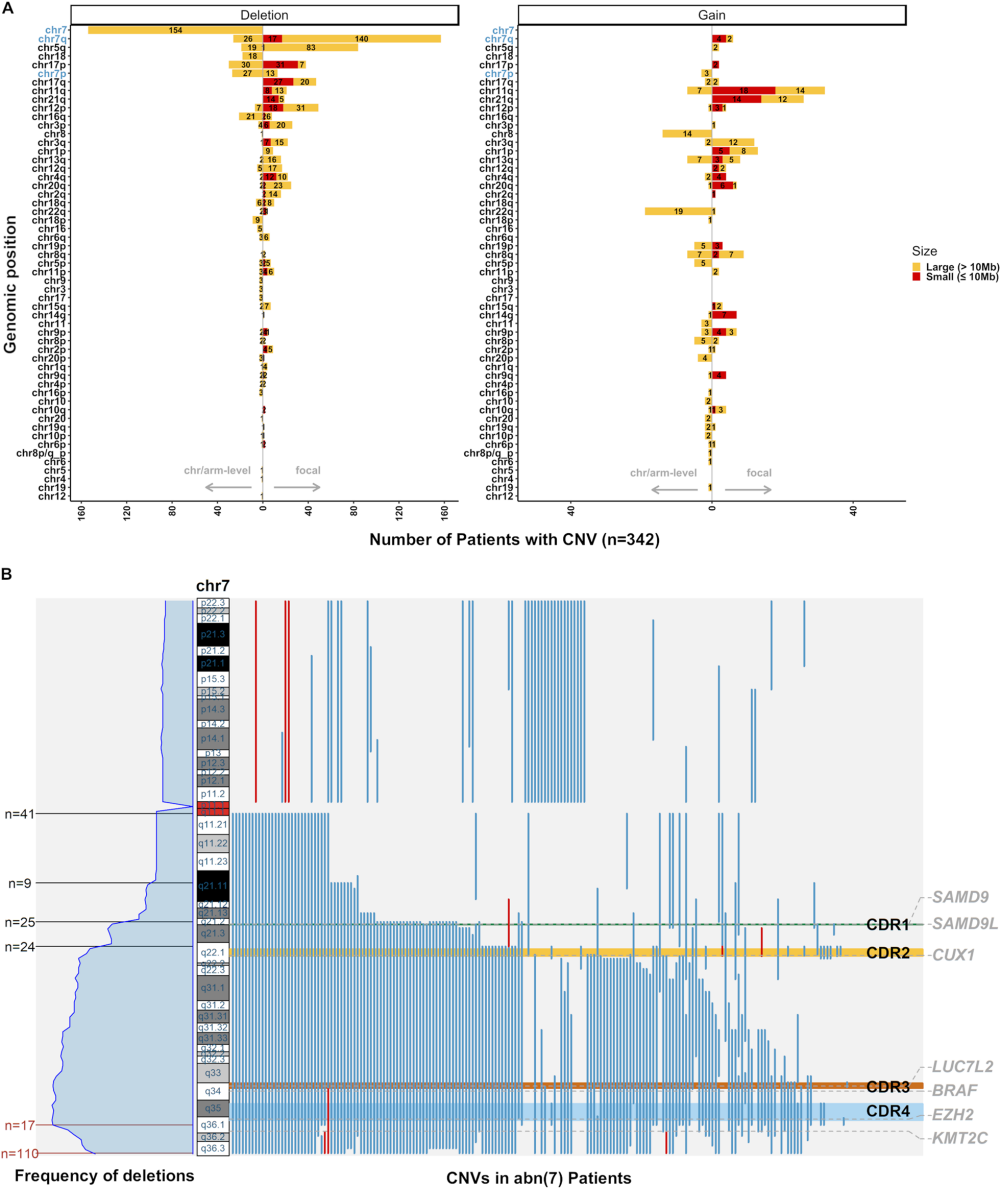
Positions and proportions of CNVs detected by TS. **A** Barplot illustrates the distribution of all 1376 manually curated CNVs derived from TS in the extension cohort (n = 342). Chromosomal CNVs spanning one arm or a whole chromosome are depicted on the left of each x-axis, while focal SNVs are shown to the right of the x-axis (according to arrows). The number of patients with a specific aberration is depicted according to the size of the event (yellow: large CNVs > 10 Mb; red: small CNVs ≤ 10 Mb). Chromosome 7 is displayed in blue. **B** To the right, genomic positions of chr7 covered by CNVs (deletions in blue, gains in red) identified in 188 AML patients (x-axis, excluding 154 cases with monosomy 7). Marked commonly deleted regions (CDRs)1–4 are adapted from Baeten et al. [16]. Potential genes of interest and their genomic positions are shown to the right. To the left, the frequency of deletions across the chromosome and recurrent breakpoint clusters, with the respective number of times a specific breakpoint occurred as a starting (black) or ending point of a CNV segment (red), are shown

Using TS-based CNV analysis, we identified a substantial number of genomic loci targeted by focal deletions/gains (≤ 10 Mb) that were missed by conventional G-banding due to its lower resolution. These focal events were most frequently observed as deletions in chr17p/q, chr12q, chr21q, and chr4q or gains in chr11p/q (Fig. 4A). In agreement with a recent study in *TP53*-mutated AML[46], most of these focal deletions affect known AML genes (Figure S12C, Table S4) such as *TP53* (chr17p13.1, 8.5%), *NF1* (chr17q11.2, 6.1%), or *ETV6* (chr12p13.2, 5.3%, Figure S13). Furthermore, we noted several previously underappreciated small CNVs: gains of *KMT2A* (chr11q, 5%), *U2AF1* (chr21q, 2.9%), and *BCL11B* (chr14q, 2%), and deletions of *RUNX1* (chr21q, 3.5%) and *TET2* (chr4q, 2.6%). ONTseq confirmed the presence of these focal CNVs in randomly chosen cases (Figure S14, Table S3). In fact, when comparing the presence of TS-based CNVs affecting chr17, we noted that 11 out of 78 patients (14% of patients) had no chr17 abnormality reported by conventional karyotyping and showed focal deletions affecting *TP53* and/or *NF1*.

Collectively, these data showed the detection advantages of next-generation-sequencing-(NGS)-based karyotyping versus conventional G-banding, as a high proportion of CNVs fell below the limit of detection through G-banding (Table S4). Furthermore, genomic regions enriched for CNVs were also common targets of somatic mutations in AML with abn(7), suggesting genetic convergence through CNVs and SNVs, a phenomenon typical for tumor suppressor genes.

### Commonly deleted regions of chromosome 7 and clonal trajectories of genomic alterations in AML with abn(7)

Previously, four CDRs of chr7q have been defined using SNP-arrays, FISH, and mCGH-assays [16]. In our study, small focal deletions affecting chr7q were found in 17 patients (5%), predominantly in CDR2 (3.5%) and CDR4 (1.2%, Fig. 4B, Table S5). The most common focal deletions affected chr7:q21.2-q36.3 (n = 31) or chr7:q22.1-q36.3 (n = 26); the vast majority resulted from two recurrent breakpoints at the beginning of CDR1 or CDR2 and reached the telomere (Fig. 4B). Next, we investigated whether the chr7-CDRs showed differences in concurrent CNVs by co-occurrence and mutual-exclusivity analysis (Figure S15). Patients with the most frequent focal del(7q)(chr7:q21.2−q36.3) were mutually exclusive to deletions affecting chr7p (*P* = 0.05, FDR = 0.07), chr17p13.3−p13.1 (region spanning *TP53-*locus, n = 19) and chr17q11.2 (region spanning *NF1-*locus, n = 16). This focal del(7q) was instead correlated with gains affecting chr3q26.2−q29 encoding the *MECOM*/*EVI1-*locus (Figure S15).

**Fig. 5:**
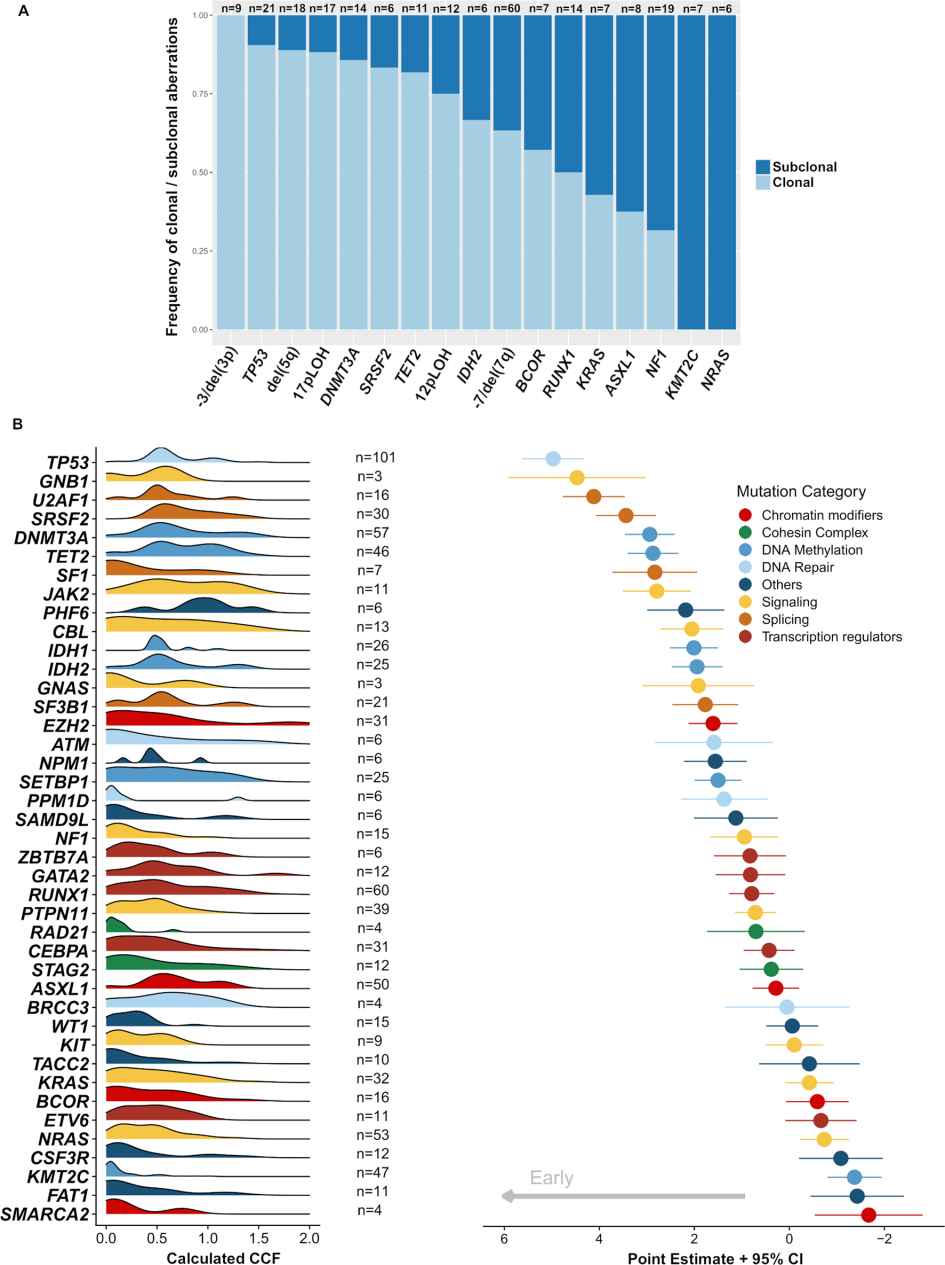
Clonal hierarchies of events in patients with aberrations in chromosome 7. **A** Clonality analysis of SNV and CNV events derived from the CCFs calculated using ASCAT [69] and CNACS [47] data in the exploration cohort (n = 60 paired diagnosis/remission samples). x/xLOH represents a chromosome or arm level CNV, and del(xp) or del(xq) represents the deletion of any part of the respective p or q arm. **B** Plot shows SNV acquisition order resulting from a Bradley–Terry model applied to mutation pairs, using the CCF calculated by correction of sample purity, ploidy, and CNV/cnLOH presence determined in pureCN (depicted on the left) in n = 342 patients. The number of mutations that entered the model is reported for each gene. To the right, the points correspond to point estimations, and the bars represent the 95% confidence intervals. Early mutations have high estimations, thus their points are arranged on the left. The median point estimation value is taken as a reference point for early versus late distinctions (grey arrow). Genes are color-coded by their assumed functional category

To investigate the clonality and timing of genomic aberrations, we applied two complementary approaches. First, CCF analysis was performed to assign CNVs and SNVs to (sub-)clonality using our WES-data (n = 60, supplemental dataset 7). CCF revealed that deletions of chr 3, 5, and 17p were mainly clonal events, suggesting their role as disease-founding aberrations. Interestingly, del(7q) and −7 were subclonal in one-third of cases and thus might appear as both an early and late event in leukemogenesis (Fig. 5A). Regarding the main abn(7) groups, −7/CK, −7/non-CK, del(7q)/CK, and del(7q)/non-CK, −7 appeared more often as clonal (88%) in −7/CK cases, while in the other three groups, the clonality of −7 and del(7q) is more evenly distributed (Figure S16). Second, our TS-based Bradley–Terry model revealed that mutations in splicing genes, in *TP53,* and in CHIP-associated genes (*DNMT3A* and *TET2)* were likely initiating events in the development of AML with abn(7). In contrast, mutations in genes involved in RAS- and Tyrosine-kinase-signaling, such as *KRAS*, *NRAS,* and *KIT,* were consistently ranked among the later events. Notably, except for *TP53*, mutations in the genomic regions frequently targeted by both CNVs and SNVs (e.g., *ETV6*, *KMT2C)* occurred at later stages of leukemogenesis (Fig. 5B).

### Survival analyses and clinico-biological correlations of abn(7) AML

Survival data were available for 518 patients. The median follow-up time for patients alive was 25 months (range, 0.2–160 months), with a median OS for all patients of 10.4 months. Due to therapeutic heterogeneity, we restricted subsequent survival analyses for clinico-biological correlations to intensively treated patients only (n = 415, Table 1). In the intensively treated cohort, the median OS was 11.9 months. While 61% of patients reached CR, the relapse rate was high at 67%.

**Fig. 6:**
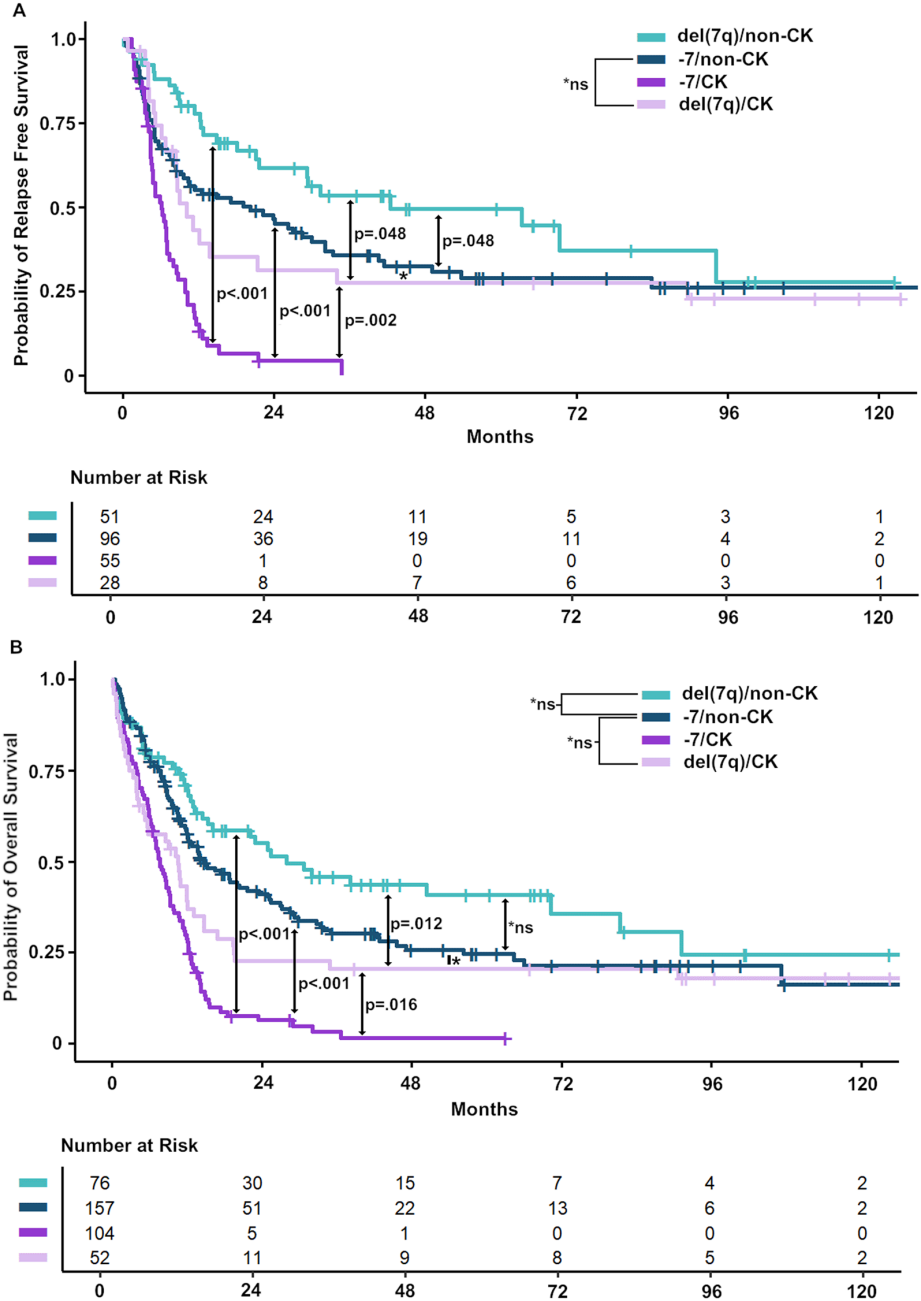
Survival analysis according to the major abn(7) groups. Kaplan–Meier Curves showing the probability of **A** RFS for n = 230 and **B** OS for n = 389 intensively treated patients with available clinical data comparing the four major abn(7) groups: −7/CK versus del(7q)/CK versus −7/non-CK versus del(7q)/non-CK. Patients classified as "other/non-CK" or "other/CK" are not included (n = 25). *P* values derived from pairwise LogRank Test, *ns = not significant

Kaplan–Meier survival analyses revealed significant differences in RFS between all main abn(7) groups [del(7q)/non-CK, −7/non-CK, del(7q)/CK, and −7/CK], except for −7/non-CK versus del(7q)/CK patients (*P* = 0.72), which seem to have comparable outcomes (Fig. 6A). For OS, the results were similar (Fig. 6B). Focusing on −7/non-CK versus del(7q)/non-CK patients, RFS was significantly inferior in −7/non-CK patients (*P* = 0.048, Fig. 6A), with a trend toward an inferior OS in this patient group (*P* = 0.081, Fig. 6B). When looking at abn(7) as sole abnormalities, we found no differences in survival endpoints between −7sole and del(7q)sole patients (Figure S17).

To evaluate the prognostic importance of clinical and genetic variables on survival and response parameters for patients with abn(7), ten variables with a significance level of P < 0.1 in univariate were included in multivariate analyses (Table 2 and Table S6). We found older age (> median, 59 years) and higher white blood cell counts (WBC > median (9/nL) to be significant clinical predictors of worse OS (HR, 1.4 [95% CI 1.1–1.8], *P* = 0.007 and HR, 1.69, [95% CI 1.32–2.16], *P* < 0.001, respectively, Table 2A), and RFS (HR, 1.47, [95% CI 1.04–2.09], *P* = 0.030 and HR, 2.23, [95% CI 1.58–3.15], *P* < 0.001, respectively, Table 2B). Furthermore, we identified *TP53*abn (= mutations and/or deletions of the *TP53* locus) and *PTPN11*mut as the strongest genomic predictors of inferior OS (HR, 2.53, [95% CI 1.66–3.86], and HR, 2.24, [95% CI 1.56–3.22], both *P* < 0.001, Table 2A, Figure S18A,B) and RFS (HR, 2.3, [95% CI 1.25–4.26], *P* = 0.008 and HR, 2.32, [95% CI 1.33–4.04], *P* = 0.003, respectively, Table 2B). The presence of a CK showed no significant influence on survival endpoints, likely due to its close association with the *TP53*abn status. Concerning response to therapy, we found *TP53*abn and *PTPN11*mut to associate with an inferior CR rate (OR, 0.44, [95% CI 0.21–0.92], *P* = 0.03 and OR, 0.45, [95% CI 0.23–0.88], *P* = 0.02, Table S6).

**Table 2:**
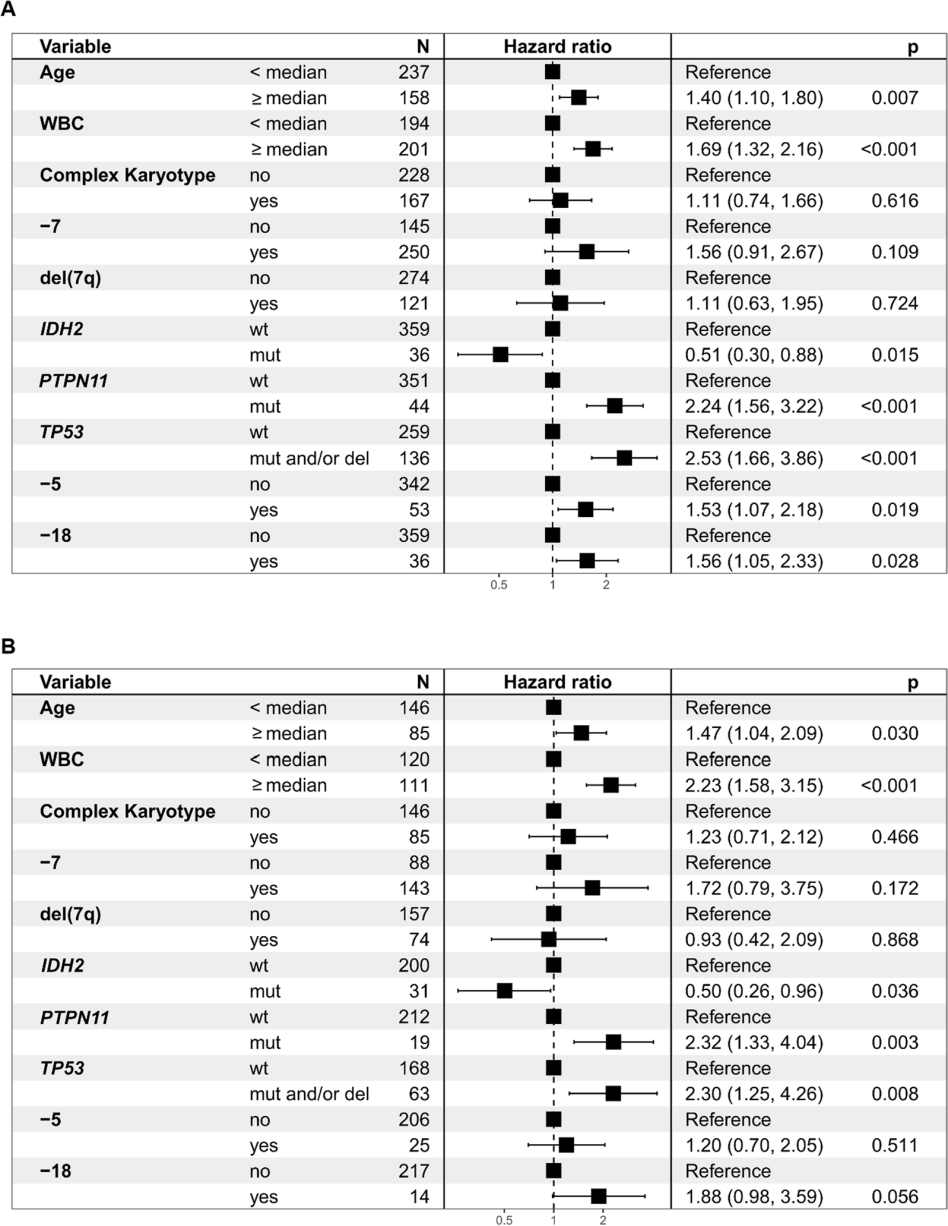
Multivariate analysis of overall survival and relapse free survival of abn(7) patients.

Tables A and B show the multivariate model for analysis of (A) OS (n = 395) and (B) RFS (n = 231) for intensively treated patients of the abn(7) cohort. In the Cox regression analysis, genomic events (gene mutations and cytogenetic aberrations) were taken as variables and potential clinical confounders as covariates. For clinical continuous variables, a separation into two groups was determined by the median value for age (59 years old) and for WBC (white-blood-cell counts, 9/nL). Genomic events were included in the multivariate analysis Cox regression analysis if they were detected in the > 5% of patients and had a univariate *P* ≤ 0.10 for OS and RFS before adjustments for multiple comparisons. Cytogenetic aberrations like −7 (Monosomy 7), del(7q), other monosomies, and complex karyotype (CK) are retrieved from clinical information. The hazard ratio is given as HR (95% of CI), and P-values are shown

Of note, *IDH2*mut was associated with better OS and RFS in our cohort (OS: HR, 0.51, [95% CI 0.3–0.88], *P* = 0.015, and RFS: HR, 0.5, [95% CI 0.26–0.96], *P* = 0.036, Table 2, Figure S18C,D), and with a higher CR rate (OR, 3.5, [95% CI 1.38–10.78], *P* = 0.01, Table S6). While allo-HSCT in CR1 generally improved OS and RFS in this cohort, *IDH2*mut patients showed an exceptional benefit from allo-HSCT in CR1 (Figure S19A-D). For *KMT2C*mut versus wild type (wt) patients, we detected no differences in clinical endpoints (Figure S20, Table S7). In fact, mutations in any of the most frequently affected genes located in the CDRs of chr7 (Figure S7A) did not influence survival endpoints (data not shown).

Focusing on *TP53* and CK-status, abn(7) patients with *TP53*wt/CK showed similar probabilities of OS and RFS compared with *TP53*wt/non-CK patients, further indicating that CK-status provided no *TP53*abn-independent prognostic information (Figure S21).

According to its proposed impact on survival in myeloid malignancies [47], we investigated the effect of *TP53* allelic status. Applying the current ICC-definition for *TP53* multi-hit status [15], we discovered that the majority of *TP53*abn patients belonged to the *TP53* multi-hit category (79%, Figure S22). Of note, in our cohort, there was no significant difference in survival between patients belonging to the *TP53* single-versus multi-hit category.

## Discussion

In this study, we deciphered the genomic landscape of adult AML with abn(7), including concomitant somatic and structural variants and their potential influence on prognosis in a large international cohort of 519 patients using a two-step NGS approach. As expected, for a large proportion of our patients, the chr7 aberration was embedded in a CK (43%), and CK-status was strongly associated with *TP53* abnormalities (*P* < 0.001) [10, 48–50]. Accordingly, *TP53* showed the highest mutation frequency (33%), and *TP53*mut appeared mostly mutually exclusive to other known gene mutations, further underscoring its importance in the current ICC-classification [15].

As a novel finding, we identified a high frequency of mutations in *KMT2C* (16.7%). KMT2C belongs to the KMT2 family of histone methyltransferases, which catalyze the methylation of lysine 4 at histone 3 (H3) and function as epigenetic regulators. The *KMT2* genes are among the most frequently altered genes in various cancer types, and *KMT2C* mutations have been mainly reported in solid malignancies to date [51, 52]. A recent study investigating the mutational spectrum of patients suffering from a wide variety of myeloid malignancies with abn(7) did not report recurrent *KMT2C* mutations [10]. Several lines of argumentation might explain this discrepancy: (1) *KMT2C* encodes for a large protein of 4911 amino acids and is characterized by a high GC-content and repetitive elements, which are both well-known factors for sequencing artifacts. We observed a similar prevalence of *KMT2C* mutations with WES and error-corrected TS and confirmed the somatic nature of these mutations using CR samples when available. (2) *KMT2C* mutations were mainly subclonal with a median VAF of 5%. Thus, studies using sequencing technologies without incorporation of error-correction are likely to miss the majority of *KMT2C* mutations. (3) Studies focusing on pediatric AML [53] and adult AML (CBF [54] and elderly [55] AML patients) reported *KMT2C* mutations at low frequencies, providing independent evidence of the true nature of these variants. Very recently, a high prevalence of *KMT2C* mutations was reported in adult blastic plasmacytoid dendritic cell neoplasm (BPDCN), affecting 48% of investigated cases [56]. With a prevalence of 45%, we found the highest *KMT2C* mutation prevalence in patients with CBF AML and abn(7). The majority of the *KMT2C* variants in our cohort were categorized as multi-hit (= concomitant mutation and deletion at the *KMT2C* locus). Considering that our clonality analyses revealed *TP53* and CHIP*-*associated gene mutations as disease-initiating and mutations of genes involved in signaling pathways and *KMT2C* as late events, *KMT2C* mutations seem to appear predominantly as the "second hit" driven by clonal selection pressure in abn(7) leukemia evolution. Collectively, these data suggest a rather supporting than initiating role for *KMT2C* mutations during leukemogenesis and disease progression. This conclusion would be backed by the report from Chen et al., where *TP53*-deficient mice only developed leukemia after transplantation of hematopoietic stem cells with concomitant *Kmt2c* and *Nf1*-knockdown [19], pointing to a collaboration of several pathways promoting leukemogenesis in a multistep manner. Therefore, it will be of interest to systematically study *KMT2C* mutations over time and investigate mutation stability and evolution in relapsing AML with abn(7). Furthermore, future studies are needed to validate the pathogenicity of the detected *KMT2C* variants.

Overall, our NGS-based CNV analysis revealed a very high concordance with diagnostic karyotyping based on G-banding/FISH analyses. In addition, this technology enabled us to calculate CN-adjusted VAFs, an essential prerequisite for investigations on clonal hierarchies, and to define precise breakpoints. Thereby, we uncovered a previously underappreciated association between the most frequent focal deletion of chr7q (chr7:q21.2–q36.3) with gains of the *MECOM/EVI1* locus (chr3q26.2–q29). Moreover, we identified a substantial number of focal lesions < 10 MB, which were likely missed by conventional karyotyping. Most of these small lesions covered loci of genes like *TP53*, *NF1*, or other recurrent AML genes, pointing toward the possibility of an even higher proportion of *TP53*-altered AML patients than currently expected.

The large size of our cohort allowed for a systematic investigation of genetic markers with patient outcome in AML with abn(7). In addition to the confirmation of the well-known detrimental outcome for patients harboring a *TP53* abnormality, we report for the first time a similar dismal prognosis for patients with *PTPN11* mutations in AML with abn(7). The prevalence of 11% in our study is slightly higher than in studies not focusing on distinct AML subgroups [57, 58]. In AML, *PTPN11* mutations mainly affect residues on the interacting surfaces of the Src-homology 2 domain and a tyrosine phosphatase domain, leading to a gain of function and activation of this proto-oncogene. This results in downstream activation of numerous pathways, including RAS/ERK1/2, FLT3, JAK/STAT, PI3K/AKT, and NF-κB, immune-evasion mediated by PD-1, and overexpression of anti-apoptotic proteins [59–61]. Furthermore, *PTPN11* and other RAS/RTK-pathway gene mutations have been associated with resistance-development to venetoclax/azacitidine treatment and poor outcome [62], indicating the high medical need for novel treatment approaches for *PTPN11*-mutant AML.

Furthermore, we observed that *IDH2-*mutant patients had a higher CR rate and longer RFS and OS than *IDH2*wt patients with AML abn(7). While being aware of the limitations of the retrospective nature of our analyses, we additionally observed a benefit of allo-HSCT in CR1 in patients with abn(7), which was especially accentuated in *IDH2*-mutant cases. The outcome of these *IDH2-*mutant patients somewhat resembled the outcome of patients with ELN 2022 intermediate-risk [3]. Importantly, this superior outcome is at least partially explained by a higher responsiveness to induction therapy and a higher usage of allo-HSCT in CR1 for *IDH2*mut as compared to *IDH2*wt patients (41% vs. 28%). These data are in line with a recent study investigating the role of *IDH1/2* mutations in 4930 AML patients that reported a significantly better RFS and OS for patients with *IDH2* mutations affecting residue R172 [63] as compared with *IDH*wt patients of the ELN 2017 intermediate and high-risk subgroups. In our cohort, both commonly affected codons (R140 and R172) showed similar superior survival. Collectively, it will be of special interest to see whether the incorporation of IDH2-inhibition leads to further outcome improvement in *IDH2*-mutant AML with abn(7).

To conclude, we retrospectively investigated the genomic landscape of adult AML with abn(7), revealing the nature of this genetically multifaceted leukemia subgroup. Its distinct clinical outcomes and genetic patterns depend on the type of abn(7) and the co-occurrence of other chromosomal aberrations. Although AML with −7 is classified into one ELN adverse-risk group, it should be noted that survival among these clinically poorly performing patients may still differ with a potential survival benefit in *IDH2*mut patients but at the same time, even poorer outcomes in the presence of *TP53* and *PTPN11* aberrations. Additional studies will be essential to elaborate on the functional consequences of *KMT2C* alterations in AML with abn(7).

### Supplementary Information

Supplementary Material 1.

Supplementary Material 2.

## Data Availability

Data for the WES and TS cohorts are summarized in the supplementary information files. All first diagnosis and complete remission WES data have been uploaded on EGA (Accession ID: EGAD50000000621).

## Abbreviations

abn(7): Aberrations of chromosome 7
Allo-HSCT: Allogeneic stem cell transplantation
AML: Acute myeloid leukemia
BIH: Berlin Institute of Health
BPDCN: Blastic plasmacytoid dendritic cell neoplasm
CBF AML: Core binding factor acute myeloid leukemia
CCF: Cancer cell fraction
CDR: Commonly deleted region
CHIP: Clonal hematopoiesis of indeterminate potential
Chr7: Chromosome 7
CK: Complex karyotype
CNV: Copy number variation
CR: Complete remission
del(7q): Deletion of the long arm of chromosome 7
ELN: European LeukemiaNet
FISH: Fluorescence in situ hybridization
HR: Hazard ratio
ICC: International Consensus Classification of Myeloid Neoplasms and Acute Leukemias
LOH: Loss of heterozygosity
NGS: Next-generation sequencing
Non-CK: Non-complex karyotype
ONT: Oxford Nanopore Technology
OR: Odds ratio
OS: Overall survival
RFS: Relapse-free survival
sAML: Secondary acute myeloid leukemia
SNP: Single nucleotide polymorphism
SNV: Single nucleotide variant
tAML: Therapy-related acute myeloid leukemia
TCGA: The Cancer Genome Atlas database
TS: Targeted sequencing
VAF: Variant allele frequency
WES: Whole-exome sequencing
WT: Wild type
−7: Monosomy 7
