# Supplementary material for: Genomic characterization of AML with aberrations of chromosome 7: a multinational cohort of 519 patients
Source: J Hematol Oncol. 2024 Aug 19;17:70. doi: 10.1186/s13045-024-01590-1 (PMC11331663; doi:10.1186/s13045-024-01590-1)
Supplement: Supplementary file 2 — Supplementary Material 2. [file 13045_2024_1590_MOESM2_ESM.docx]

Supplemental Data

**Genomic characterization of AML with aberrations of chromosome 7: a multinational cohort of 519 patients**

Adriane Halik^1^*, Marlon Tilgner^1^*, Patricia Silva^1^*, Natalia Estrada^1^, Robert Altwasser^1^, Ekaterina Jahn^2^, Michael Heuser^3,4^, [Hsin-An Hou](https://pubmed.ncbi.nlm.nih.gov/?sort=date&term=Hou+HA&cauthor_id=30610028)^5^, Marta Pratcorona^6^, Robert K Hills^7^, Klaus H Metzeler^8^, Laurene Fenwarth^9^, Anna Dolnik^1^, Christine Terre^10^, Klara Kopp^1^, Olga Blau^1^, Martin Szyska^1^, Friederike Christen^1^, Jan Krönke^1^, Loïc Vasseur^11^, Bob Löwenberg^12^, Jordi Esteve^13^, Peter J M Valk^12^, Matthieu Duchmann^14^, Wen-Chien Chou^5^, David C Linch^15^, Hartmut Döhner^2^, Rosemary E Gale^15^, Konstanze Döhner^2^, Lars Bullinger^1,16^, Kenichi Yoshida^17^, and Frederik Damm^1,16^.

# Supplemental Methods

## WES in the exploration cohort

### WES Analysis of SNVs and SBS signatures

Our pipeline for variant filtering in WES included the following steps:

Exclusion criteria:

- P-value by EBCall^1^ > 10^-4^
- P-value by Fisher's test (after realignment) > 10^-1.3^
- P-value by Fisher's test > 10^-1.3^
- variant allele frequency < .05
- strand ratio in tumor = 0 or 1
- genomicSuperDups (excluding *NF1*)
- exonic function defined by annovar = unknown
- SNPs with minor allele frequency > .001
- mutated reads in normal sample > 4 (less contaminated samples)

Sequencing coverage was 248-fold (115x-459x) for diagnosis samples and 238-fold (98x-571x) for remission controls.

For validation of the detected SNVs, we performed amplicon-based ultra-deep sequencing (n=74) and Sanger sequencing (n=30). In total, we were able to validate 10% of all 932 detected variants. For targeted deep sequencing, libraries were prepped using NEBNext Ultra DNA Library Prep Kit (New England Biolabs) and paired-end sequenced on a MiSeq sequencer (Illumina, Illumina, San Diego, CA, USA), as previously described^2-5^. Further validation was conducted visually via the Integrative Genomics Viewer (IGV)^6^.

Gene Panel Design: The DKMSv05 Gene Panel served as the basis for our customized gene panel. Further genes were selected according to

1. their appearance in our WES SNV data (≥2 SNVs per gene, rescue of genes known to be recurrently mutated in AML or other haematological malignancies if ≥1 SNV present)
2. other AML gene panels (The Cancer Genome Atlas [TCGA]^7^, Eisfeld *et al*.,^8^)
3. Data comparison with known databases or -sets (COSMIC database, Genomic Data Commons (GDC), BEATAML, Target-AML, Target-ALL and TCGA-LAML)
4. Exploration of gene enrichments within the 742 mutated genes using a functional interaction network over the Reactome database (CytoscapeVersion: 3.10.2 and ReactomeFIVersion 8.0.6 (from 2022)) for exploration of pathways and biological functions of different proteins/clusters (derived from several databases CellMap, Reactome, KEGG, NCI PID, Panther, and BioCarta)
5. Additional extensive literature research

The final gene panel included 66 genes of interest and a SNP-backbone based on the WES CNV data (**Tables S1 and S3**, supplemental Dataset 7) and consisted of a total number of 7080 probes with a target size of 466735 bp.

To understand mutational processes underlying leukemogenesis, we investigated single base substitution (SBS) mutational signatures from WES data. To this end, we performed signature extraction via the hierarchical Dirichlet process (HDP; https://github.com/nicolaroberts/hdp) as previously described^9^, and the expectation-maximization algorithm was used to deconvolute the extracted signatures into known COSMIC (v3.4) signatures in AML (SBS-1, SBS-5, SBS18, and SBS-31)^10^ or those identified in normal blood cells^11^.

### WES Analysis of CNV

To correlate existing karyotype information with WES-informed CNVs, we used an in-house pipeline applying ASCAT^12^ and CNACS^13^ as previously described^14^. The results were manually validated and subsequently merged to highlight the distribution of frequent gains and deletions per chromosome (**Figure S11A**).

## Targeted NGS approach in the extension cohort

### Analysis of SNVs

Our in-house Snakemake pipeline was applied as previously described^3^. In which consensus reads were aligned to the GRCh38.p13 reference, variant calls were retrieved as before, and annotation *dbSNP155* was added. To ensure the quality of data, only samples with a minimum of 99-fold coverage over the 66 genes were considered, and second attempts were made for samples with a median coverage <200-fold.

To overcome the limitations associated with the lack of paired samples for germline determinations in our TS approach, our filtering criteria were optimized by comparison of TS data with results from paired tumor-germline WES data. Variant filtering steps for TS samples are outlined in **Figure S1**. For internal validation, we included DNA specimens from 19 healthy controls. Candidate variants were extensively inspected for artifacts and mapping/calling errors through visual inspection with the Integrative Genomics Viewer (IGV)^6^. For selected samples, further validation steps included Sanger Sequencing, as previously reported^3-5^.

For *ASXL1*, we found a high mutation frequency, especially on position c.1934dupG, which has been linked to false positive artifact-related findings before. Hence, we performed an additional qPCR validation as described by Yannakou *et al.*^15^ using the G9 primer and as a positive control of the cell line Kasumi-1 (DSMZ, Braunschweig, Germany)^15^. According to these results, we considered validated *ASXL1* c.1934dupG mutations at VAF ≥15% from TS calculation and kept three mutations with a VAF of <15% after qPCR validation in non-WGA samples.

*FLT3*-ITD screening was performed using Genescan-based fragment analysis, enabling quantification of the allelic ratio performed by contributing centers^16-18^.

### TS Analysis CNVs

Copy-number variants were evaluated from target sequencing data of the in-house designed SNP-backbone. Putative somatic copy-number variations were obtained by application of the *PureCN* ^19^ algorithm to estimate tumor purity and ploidy, local copy numbers, and deletions of heterozygosity (LOH). Some samples presented with high CoefVar (>50) for any purity-ploidy pair considered or had no segment in chromosome 7 that reached a log(CN)>0.4 or a log(CN)<-0.3 and were therefore not used for CNV analysis. To consider a possible cnLOH (for manual curation), we required a segment to have –0.3<log(CN)<0.4 and an accompanying 0<BAF<0.33.

Arm-level aberrations were defined when CNV affect at least until the beginning of the last covered band of an arm, taking into account the observed coverage of segmentation results provided by *PureCN* (**Figure S11B**). Two arm-level events were manually merged into a chr-level CNV.

For a comparison between conventional G-Banding karyotype information and our CNV results, we used the five most frequent events reported in the karyotypes. These were deletions in chromosomes 5, 16, 17, 18 and gains in chromosome 8, for which we calculate agreements between karyotype and our short-read sequencing of SNP-backbone. We used K from Cohen's Kappa to compare the results of two techniques applied to the same sample. Considered a CNV detection possible with TS in a sample with the following karyotype information:

- Detection was expected for gains in chr8: +8 and iso(8)/hsr(8)/add(8)/i(8) in the absence of a derivative or marker chromosomes declaration (which were removed from analysis). Failure of detection was also expected for +8 or add(8) visualized in G-banding only in a small number of metaphases (~2).

- Detection was expected for deletions in chr5, 16, 17, and 18 if complete partial or chromosomal deletions, derivative or dicentric chromosomes, and isochromosomes were reported. All possibly ambiguous karyotypes were removed from the analysis (i.e., -16 and +16 in different metaphases with nearly the same occurrence frequency).

For additional internal validation of our CNV events, we conducted long-read Oxford Nanopore Technology (ONTseq) for 33 patient samples as described^20^. For each sample individually, QDNAseq^21^ was used to bin the data and infer the copy numbers for different bin sizes. Visualization and noise estimation of agreements between different segmentation lengths of the long-reads (100Kb, 500Kb, and 1000Kb) was done in IGV^6^. We used K from Cohen's Kappa to estimate agreements between these techniques applied to the same sample using the above-described frequent cytogenetic events (**Figure S14**).

## Cancer Cell Fraction Analysis and Bradley-Terry Model

The CCFs used for the determination of the clonal status of genetic alterations from the exploration cohort were calculated as VAF * (1/p), where p means tumor purity estimated by CNACS^13^ with a 95% confidence interval (CI). Genetic aberrations laying within this CI were labelled as "clonal", and variants exceeding the CI were classified as "subclonal". CCF of mutations affected by concomitant copy number changes were corrected as VAF * (1/p) * (p*CNt + CNn(1-p)), where CNt and CNn mean local tumor and normal copy numbers.

The temporal order of mutations was estimated from the TS data of 342 good-quality samples of the extension cohort and computed according to the Bradley-Terry model, in which the temporal order was estimated (point estimate with correspondent 95% CI)^2^, on the basis of CCF calculated from observed VAF of the mutations and the presence of CNVs/cnLOH similarly to what was done before^22^. For each gene-sample pair, the mutation with the highest VAF was used. To apply the Bradley-Terry model, CCFs of coexisting mutations of the same sample were pitted against other mutations. The mutation with a larger CCF was assigned as a winner, and the opposite variant was designated as a loser. Genes that participated in the model 3 times and won at least once were considered.

Some adaptations of our previously described CCF calculations^22^ were necessary due to the low number of mutations per sample in the extension cohort:

-Good read depths were observed with a mean of 742-fold and minimum of 97-fold; therefore, no cut-off was established.

-Restriction of Indels to size with a more reliable VAF for CCF calculation was done by excluding Indels with >15 bases changed between reference and alternative alleles.

-For tumor purity-ploidy pairs, we used the most likely pair calculated by the PureCN algorithm under the restriction of ploidy to the range of 1.5-3N and the sample quality restrictions described.

-The general formula of mutation cellular fractions solved for CCF was applied from recent reports^22,23^ and restricted to CCF≤2.

-Deletions that were rounded to CN=0 but where a mutation is found were considered to have a CN=1.

-Since accurate estimations of the correct CN for gains are complex, we tentatively assumed it as three for all reliable gains with CN>3. As aberrant CCFs were not included in the analysis (CCF>2), only 12 gains were considered within the remaining 1124 CCFs.

## Statistical analysis

Statistical analysis was performed using R version 4.4.3 and RStudio version 2023.12.1+402. The *discover* package was applied for co-occurrences and mutual exclusivities^24^, and odds ratios (OR) were calculated with *glmuni* and *glmmulti* functions from *finalfit*^25^. The quantification of agreements between two rater techniques was estimated by calculation of K from Cohen's Kappa with *irr* package. Primary analysis was performed on OS. Sensitivity analyses were performed on CR and RFS, and results are displayed for exploratory purposes. OS endpoints, measured from the date of first diagnosis, were death (failure) and alive at last follow-up (censored). RFS endpoints, measured from the date of documented CR, were relapse (failure), death in CR (failure), and alive in CR at last follow-up (censored). Pairwise comparisons of variables for exploratory purposes were performed using Wilcoxon rank sum, Pearson's Chi-squared, or Fisher's exact tests. Kaplan-Meier analysis was used to create survival curves. Differences between subgroups and survival curves were examined via log-rank tests. Multivariate Cox proportional hazard models were used to investigate associations between various factors and survival endpoints (OS and RFS). For CR, logistic regression models were applied. Cytogenetic aberrations and gene mutations were included in the multivariate models if they were detected in >5% of patients of the cohort with an unadjusted univariate P≤.1. P-values were considered statistically significant if P reached ≤.05.

# Supplemental Figures


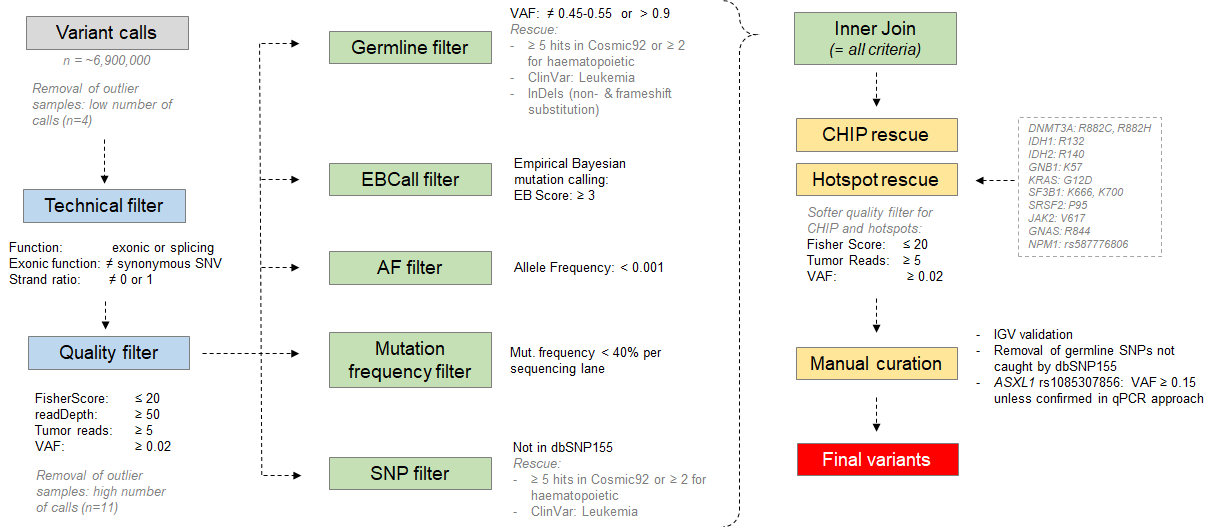


**Figure S1. Pipeline for filtering variant calls from targeted sequencing**

**A**


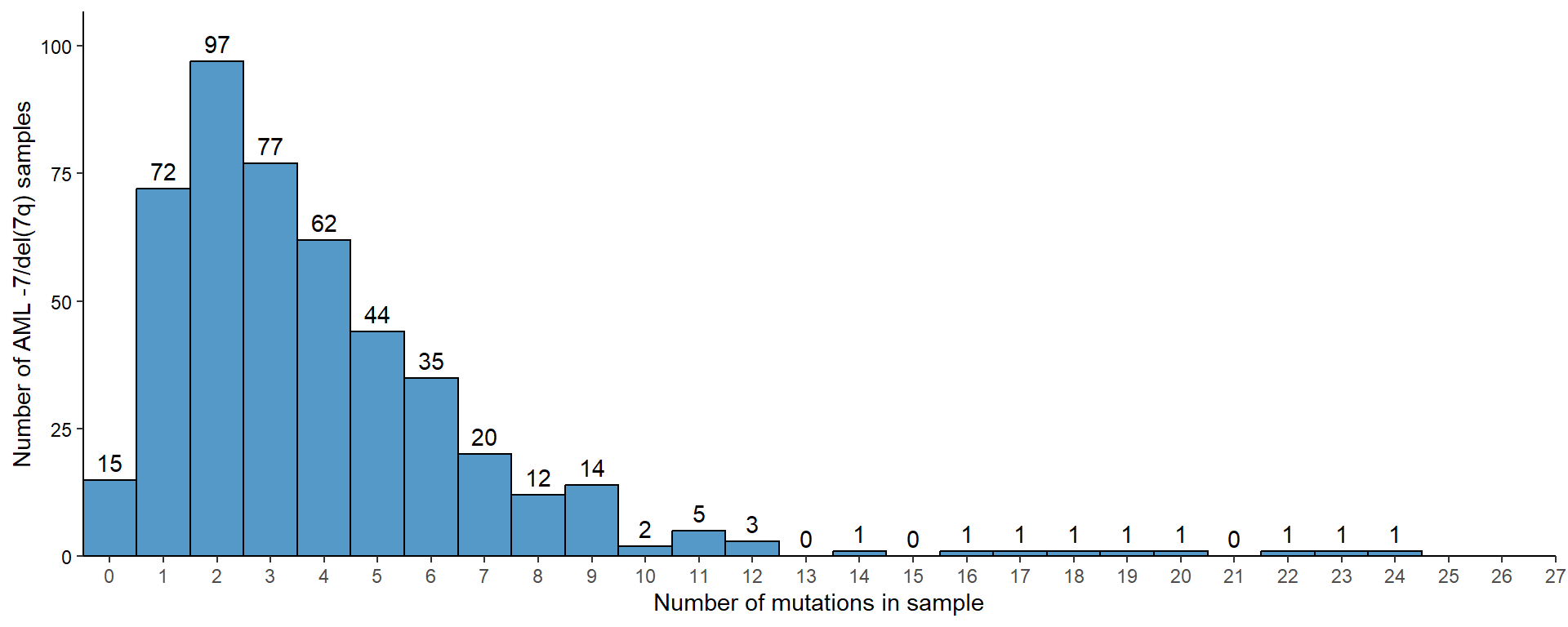


**B**
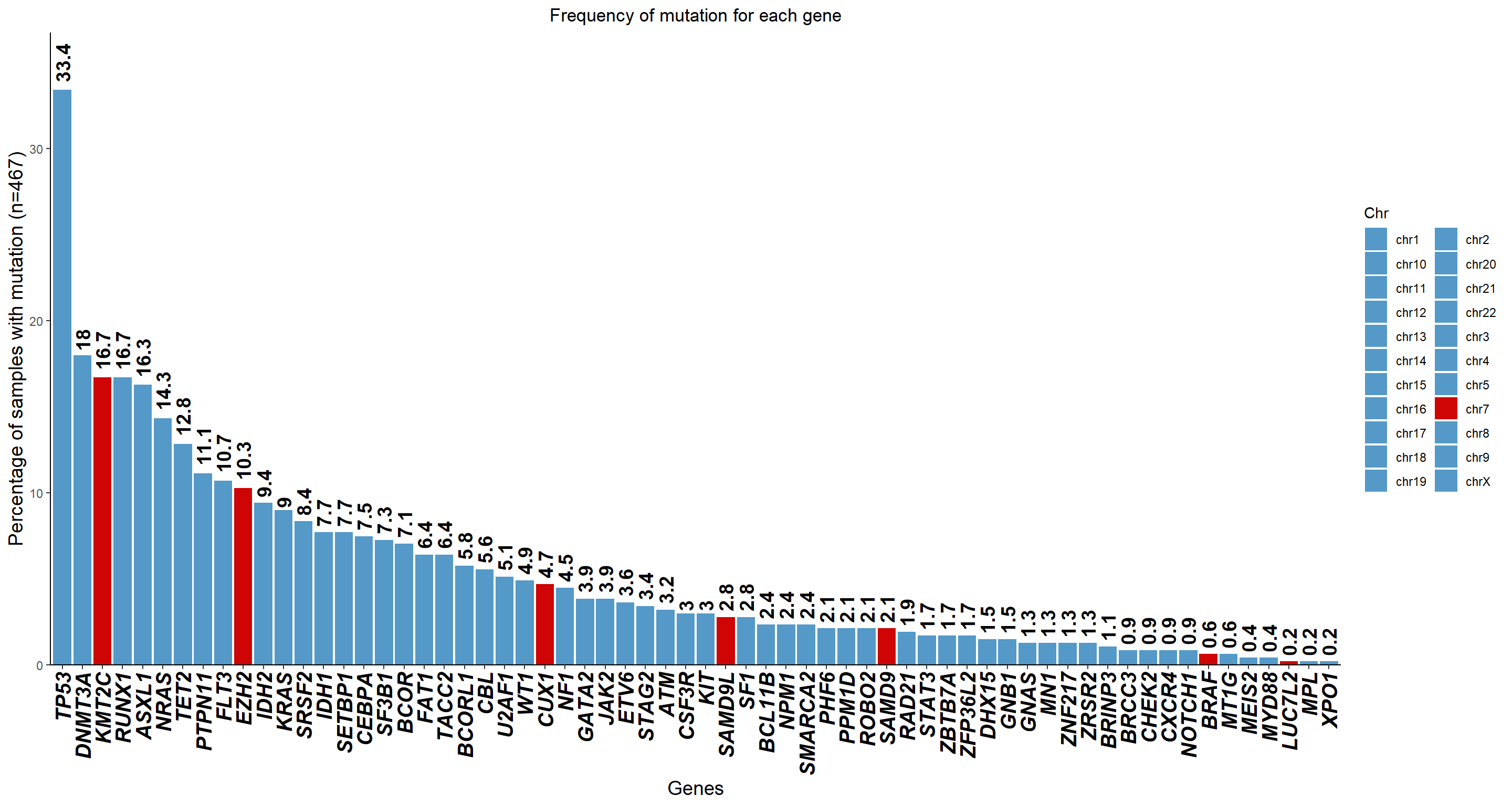


**Figure S2. Mutation frequencies detected by targeted sequencing**

1. Histogram reports the number of samples from the abn(7) exploration cohort carrying each number of mutations.
2. The bar graph shows mutation frequencies identified by TS in patients (n=467) per gene for all 64 genes mutated. Bars colored in red mark genes with genomic localization in chromosome 7. The frequency of mutations in the gene is shown in the graph above in percentage. *FLT3* represents an entity that includes all mutations found in TS, including 11 detected *FLT3*-ITDs and 20 additional *FLT3*-ITDs detected only by respective contributing centers through Genescan analysis.

| **A** | **B** |
| --- | --- |
| 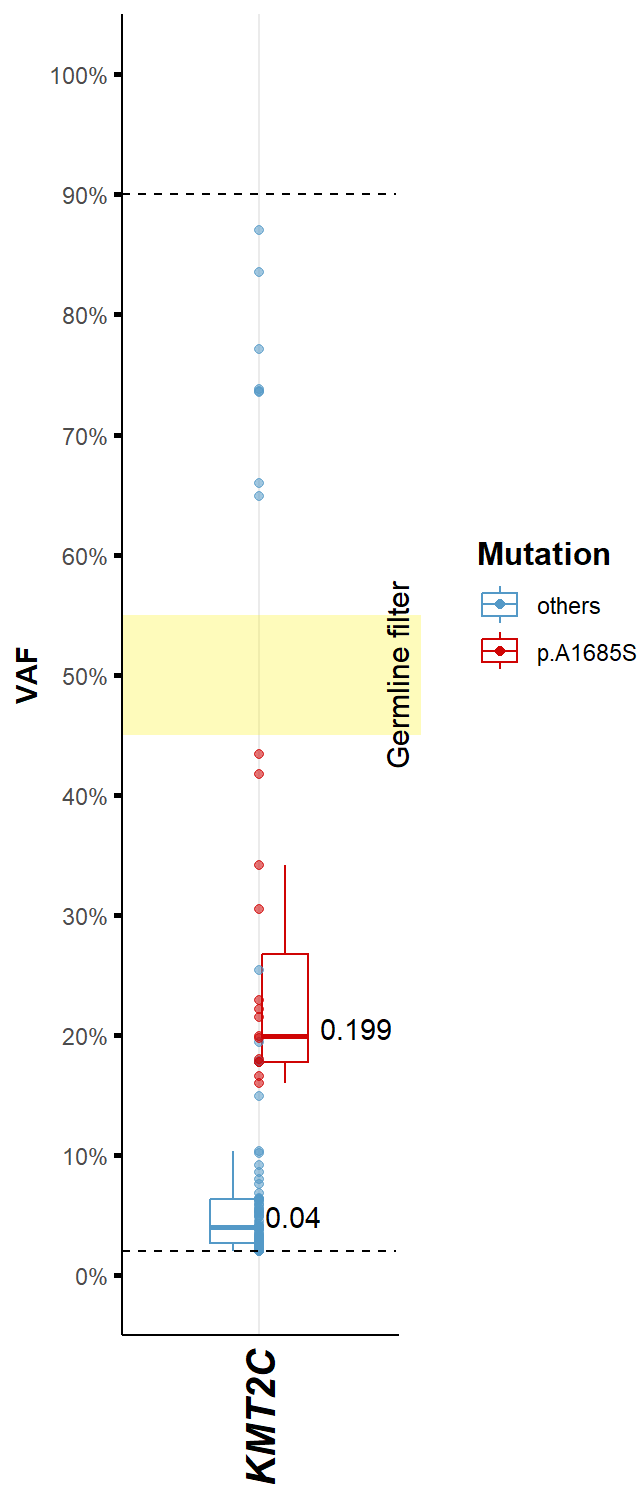 | 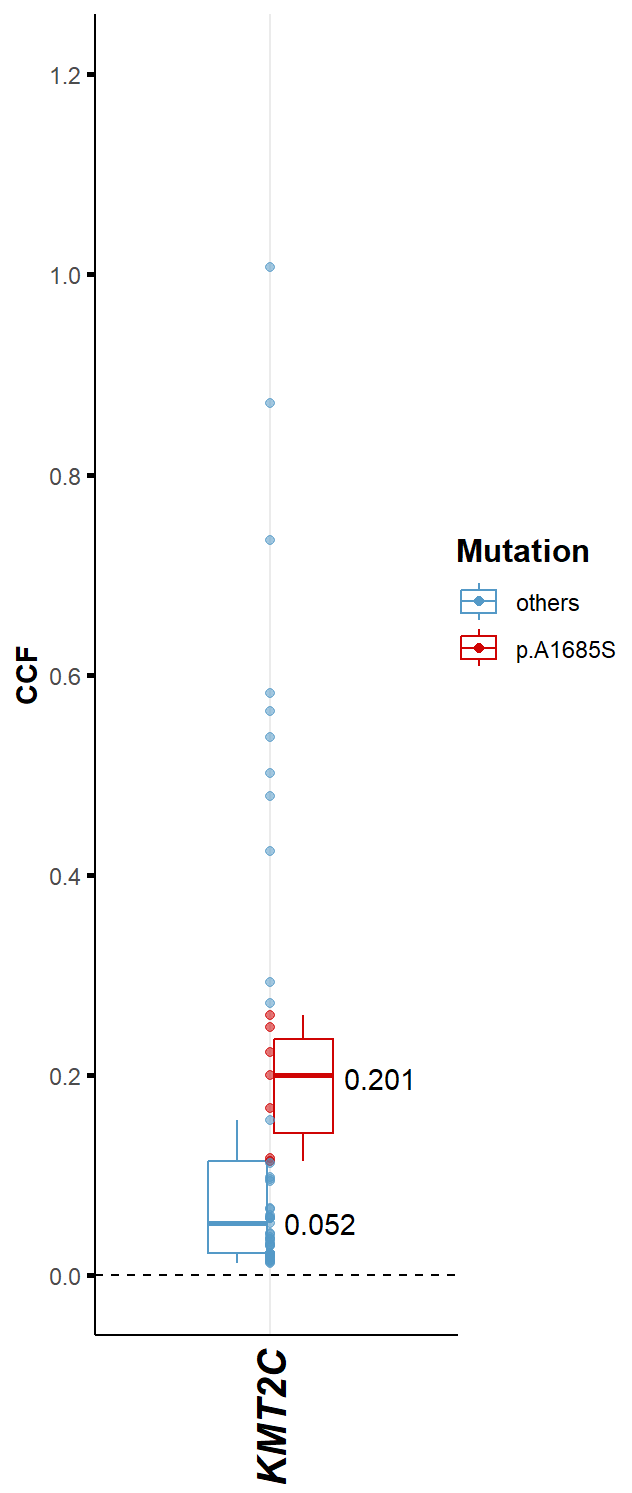 |

**Figure S3. Low allele-burden *KMT2C* mutations.**

Boxplots showing (A) the variant allele frequencies (VAF, n=89 mutations, A1685S: n=15) calculated according to variant vs reference proportions of reads uncorrected for copy number affecting aberrations and (B) cancer cell fractions (CCF, n=56 mutations, A1685S: n=7) calculated from VAFs and corrected by copy-number and sample purity. The hotspot mutation is marked separately in red (A1685S) and all others in blue. The germline filters for VAF and VAF thresholds applied in the TS data are shown.

**A**


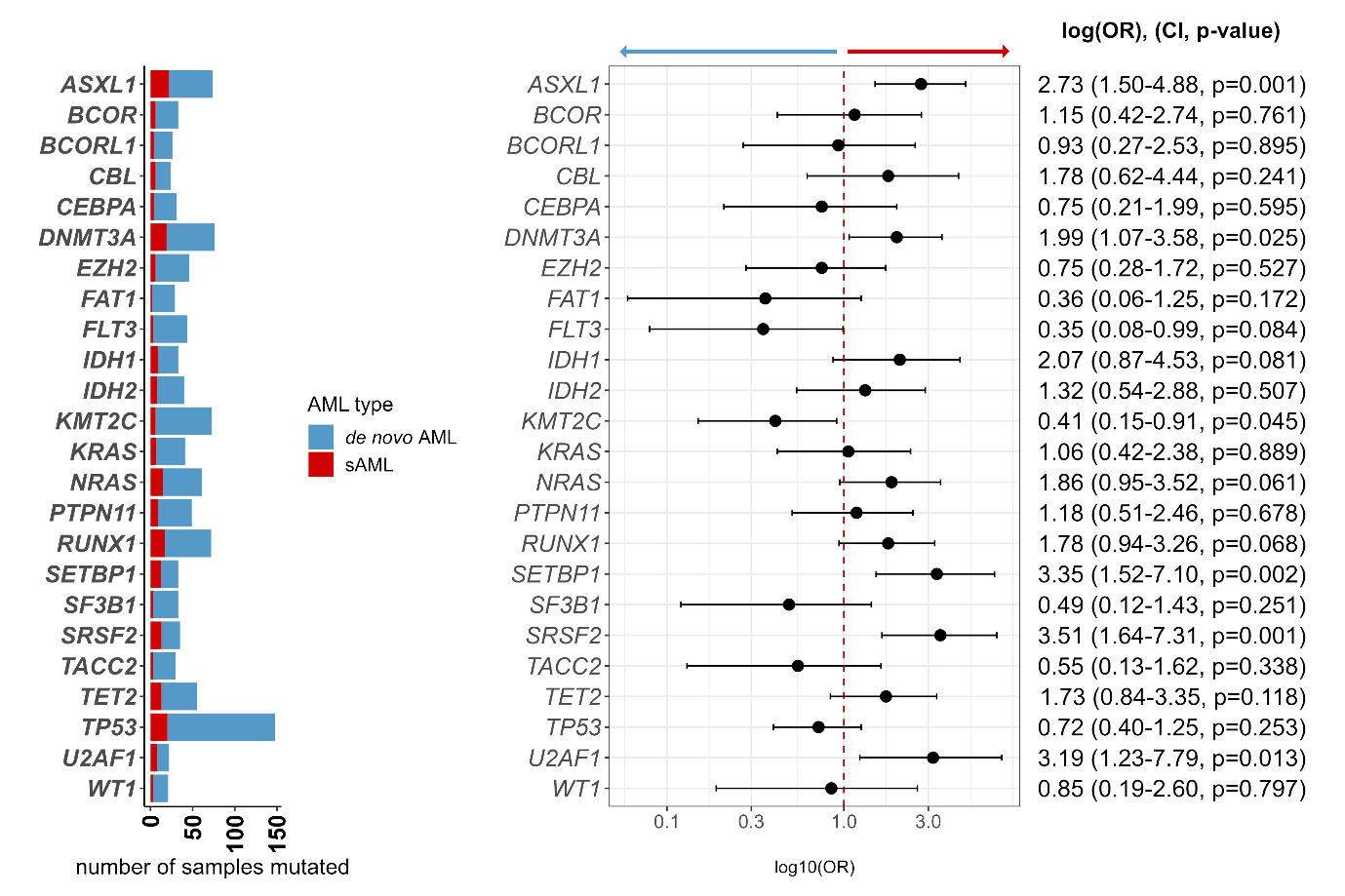


**B**

**
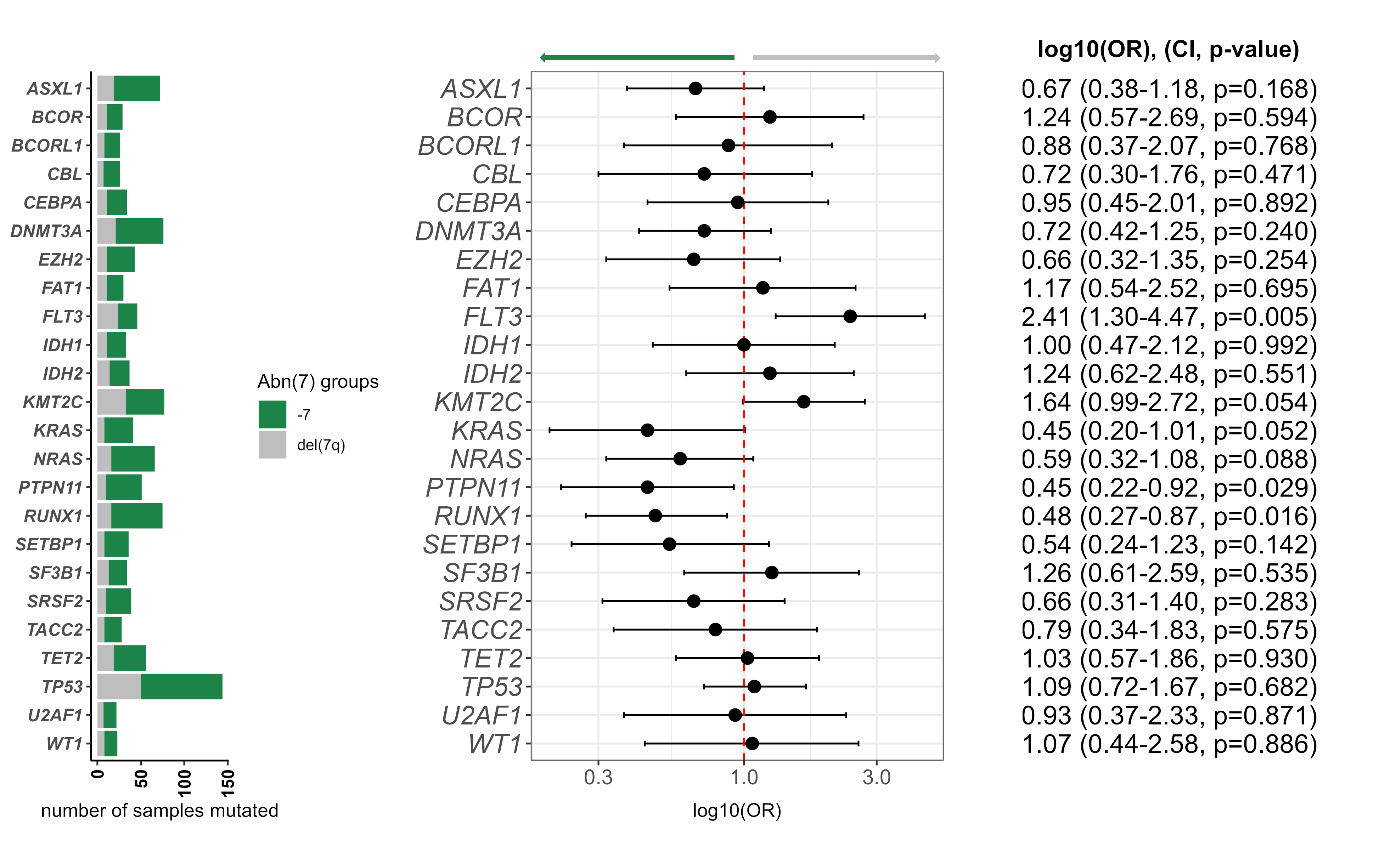
**

**Figure S4.** **Association of gene mutations with clinical characteristics of AML, *de novo* AML vs sAML and with cytogenetic aberrations, del(7q) vs –7.**

Odds ratio plot for the results from multiple univariable binomial logistic regressions fitted for each gene. The bar plot on the left depicts the number of samples included in tests for the genes tested. Genes were tested to determine if they have mutations in both groups *de novo* AML and sAML (A) or in del(7q) and –7 (B), and a minimum of 23 total mutations in the extension cohort (frequency of ≥5%). On the right-side log(OR) is given with a confidence interval of 95%, CI, and P-value.


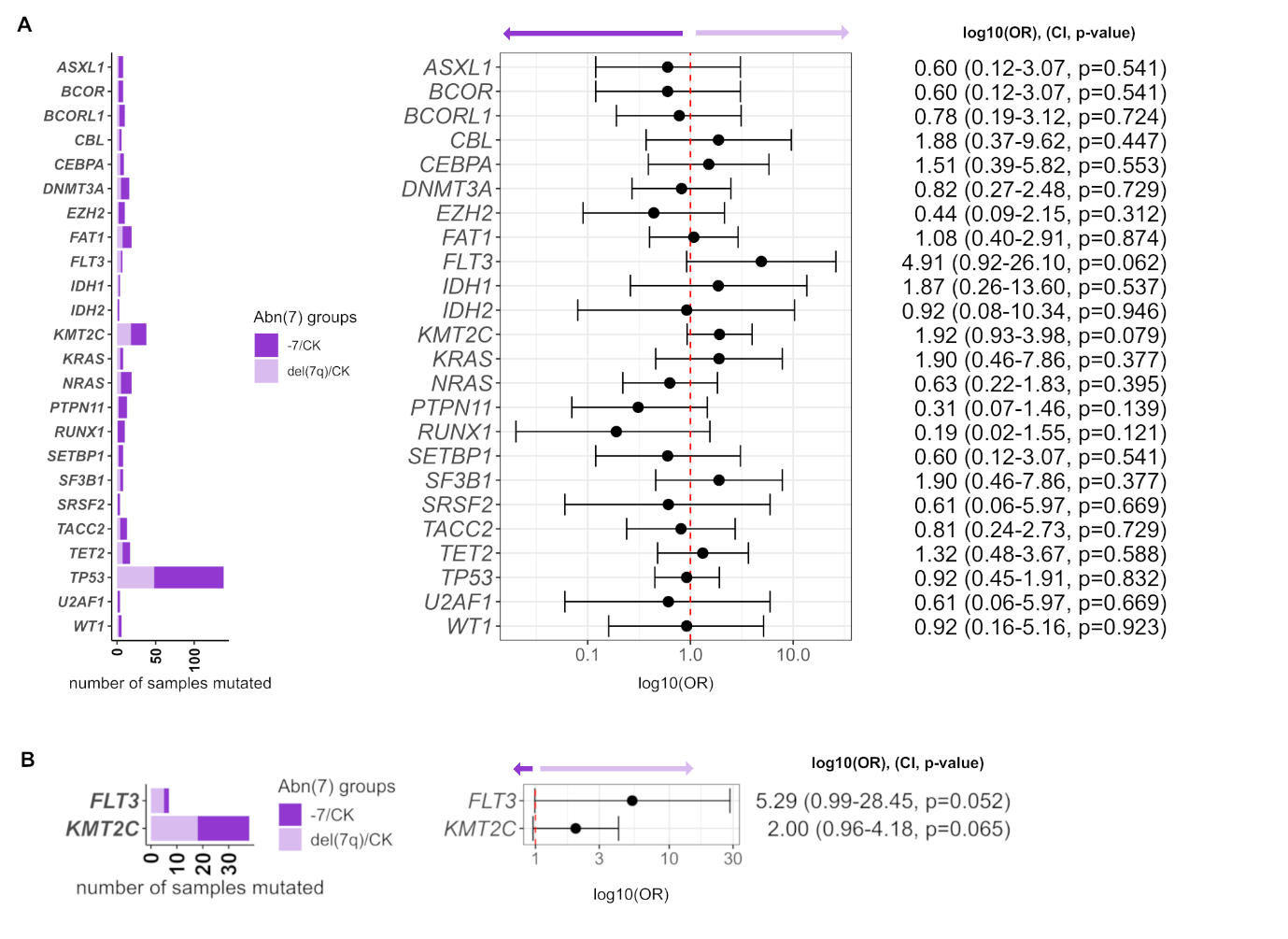


**Figure S5. Association of gene mutations with del(7q)/CK vs -7/CK patients.**

1. Odds ratio plot del(7q)/CK vs. -7/CK groups with results from multiple univariable binomial logistic regressions fitted for each of the 24 genes. The bar plot on the left depicts the number of samples included in tests for the genes tested. Genes were tested to determine if they have mutations in both groups and a minimum of 23 total mutations in the extension cohort (frequency of ≥5%).
2. Odds ratio plot for the results from a multivariable binomial logistic regression fitted for all genes that showed a tendency (with a P<.1) in the univariable fitting. The bar plot on the left depicts the number of samples included in the test for the 2 genes included in the multivariate model. On the right side logOR is given with a confidence interval of 95%, CI, and P-value.


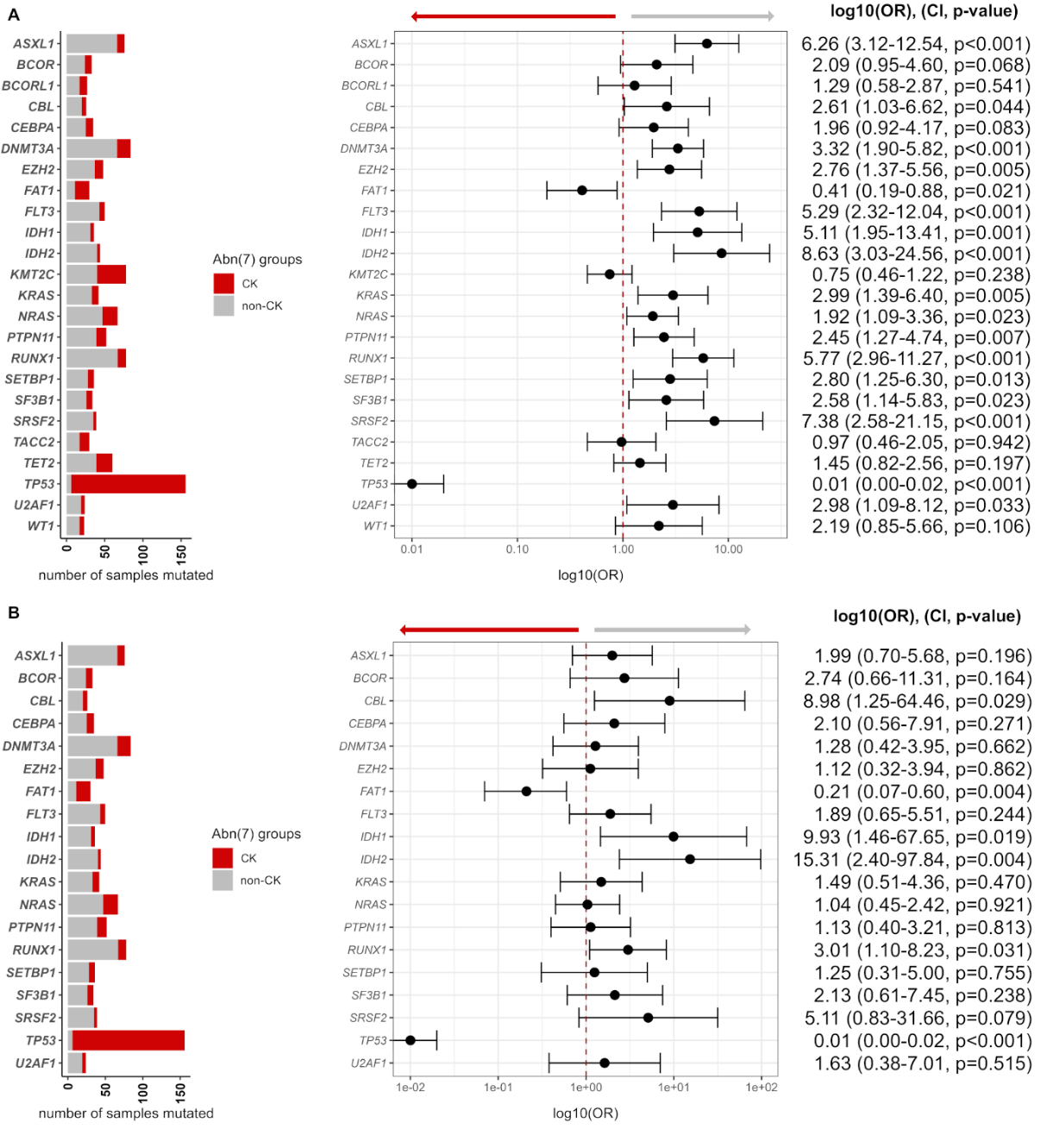


**Figure S6. Association of gene mutations with abn(7) non-CK vs CK patients.**

(A) Odds ratio plot abn(7)/non-CK vs. abn(7)/CK groups with results from multiple univariable binomial logistic regressions fitted for each of the 24 genes. The bar plot on the left depicts the number of samples included in tests for the genes tested. Genes were tested to determine if they have mutations in both groups and a minimum of 23 total mutations in the extension cohort (frequency of ≥5%).

(B) Odds ratio plot for the results from a multivariable binomial logistic regression fitted for all genes that showed a tendency (with a P<.1) in the univariable fitting. The bar plot on the left depicts the number of samples included in the test for the 19 genes included in the multivariate model. On the right side logOR is given with a confidence interval of 95%, CI, and P-value.

**A**


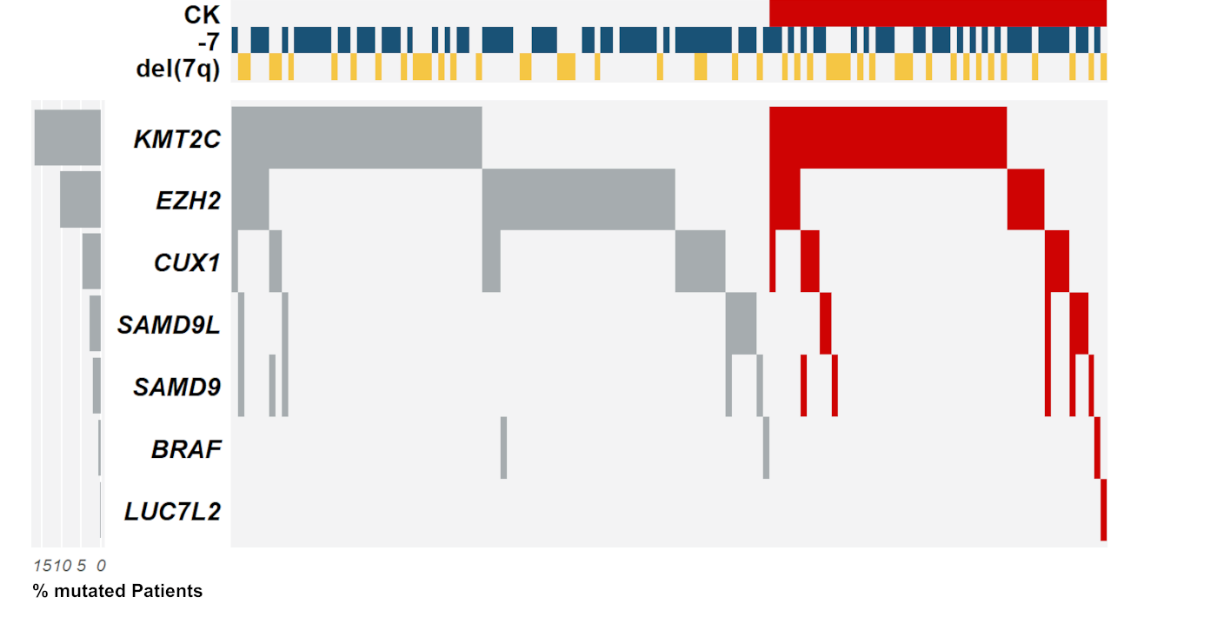


**B**


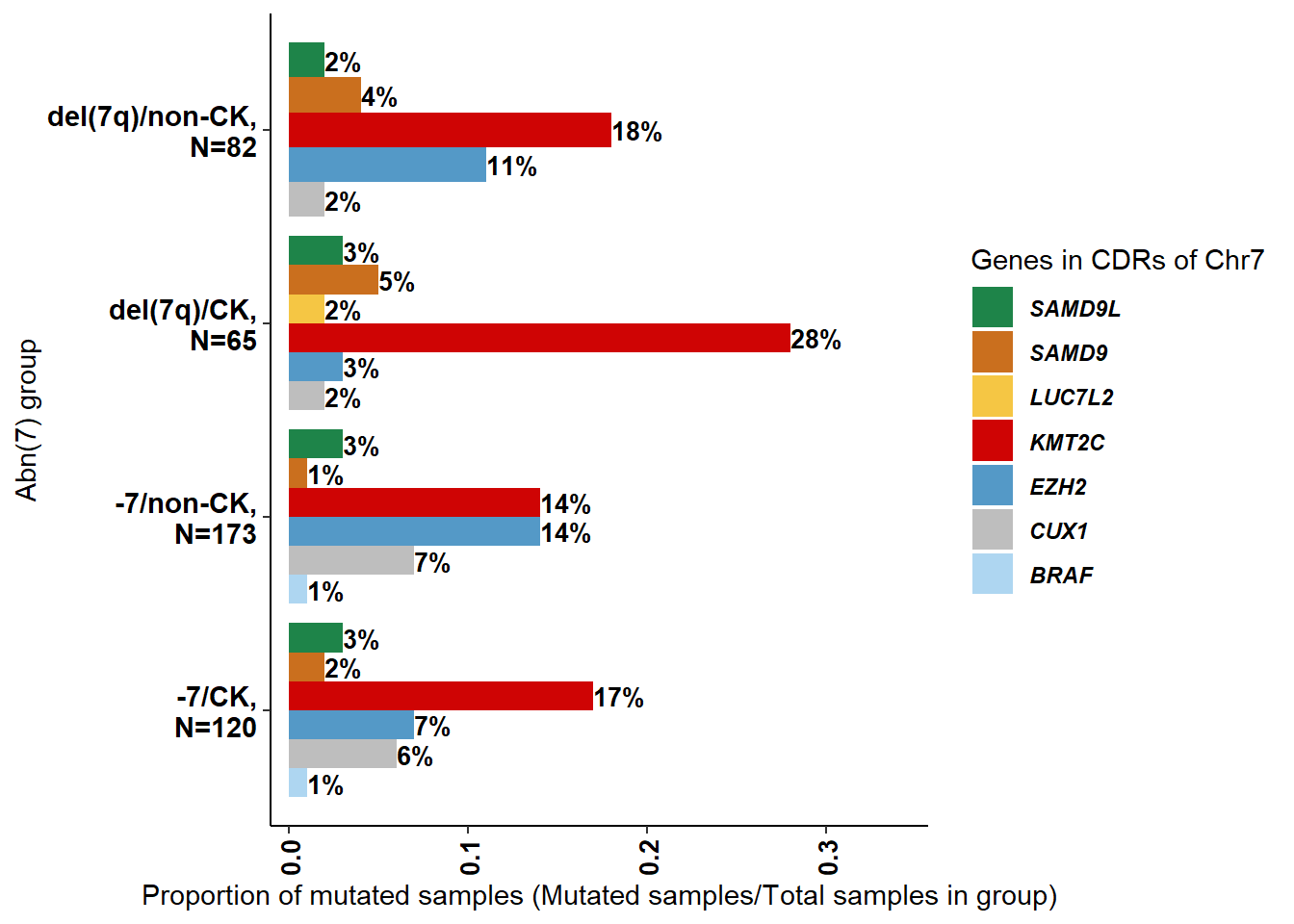


**Figure S7.** **Mutations located in genes from the CDRs of chromosome 7.**

1. Oncoplot showing the mutations found in 7 genes located on chromosome 7. Mutations were found across 140 of the 467 patients of the extension cohort (30%). Patients were segregated according to karyotype information into two major groups: abn(7) / non-complex karyotype (non-CK, grey) or abn(7) / complex karyotype (CK, red), as defined by the presence ≥3 cytogenetic alterations (according to ICC recommendations^26^. On top rows are marked patients that could be classified from cytogenetic information with a -7 (dark blue) or del(7q) (yellow). On the left, a bar plot shows the frequency (%) of patients with alterations found in each gene from n=467 patients.
2. Bar graph showing the proportion of patients with a mutation in a gene of chromosome 7 CDRs surveyed by TS, grouped according to karyotypes del(7q), or –7 and CK or non-CK. Frequency of patients with these mutations are given in the bars calculated according to the number of patients per group shown in the y-axis (N). Samples with other types of cytogenetic aberrations of chr7 were excluded (n=27).


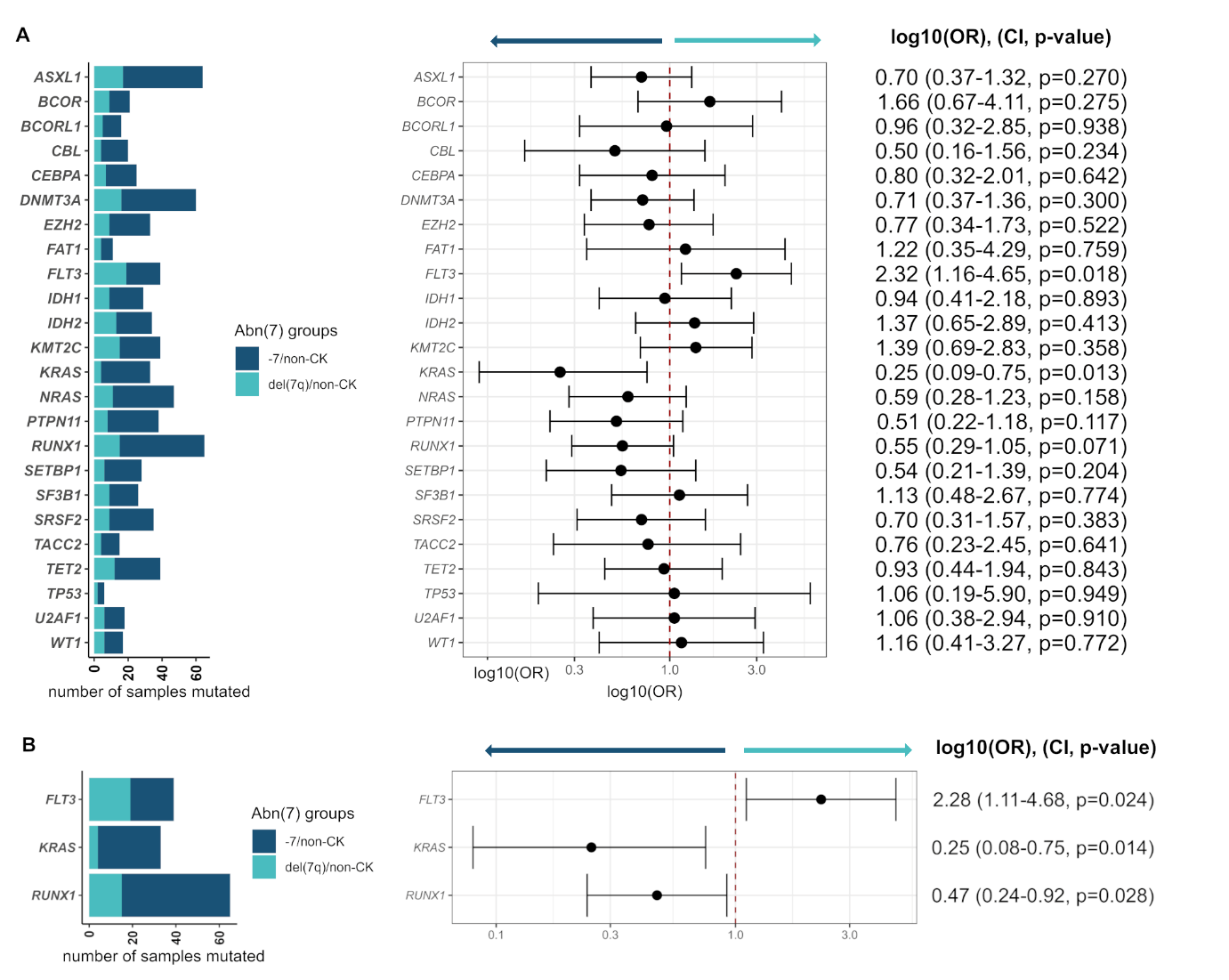


**Figure S8. Association of gene mutations with -7 vs del(7q) patients with a non-complex karyotype (non-CK).**

1. Odds ratio plot for the results from multiple univariable binomial logistic regressions fitted for each gene. The bar plot on the left depicts the number of samples included in tests for the genes tested. Genes were tested to determine if they have mutations in both groups -7/non-CK and del(7q)/non-CK and a minimum of 23 total mutations in the extension cohort (frequency of ≥5%).
2. Odds ratio plot for the results from a multivariable binomial logistic regression fitted for all genes that showed a tendency (with a P <.1) in the univariable fitting. The bar plot on the left depicts the number of samples included in the test for the three genes included in the multivariate model. On the right side, logOR is given with a confidence interval of 95%, CI, and P-value.


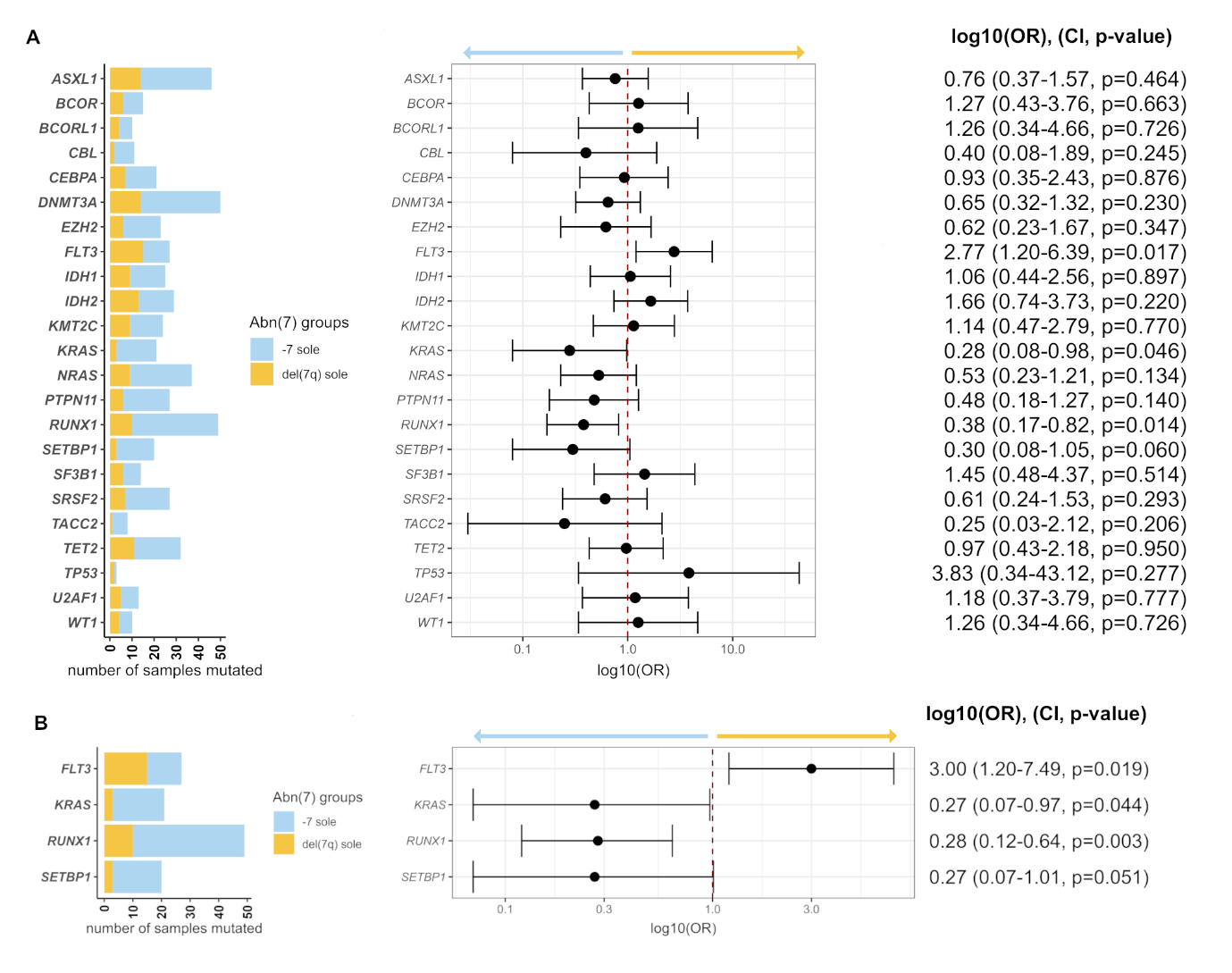


**Figure S9. Association of mutations with -7 vs del(7q) patients within samples with cytogenetic aberrations only in chromosome 7 (sole).**

1. Odds ratio plot for the results from multiple univariable binomial logistic regressions fitted for each gene. The bar plot on the left depicts the number of samples included in tests for the genes tested. Genes were tested to determine if they have mutations in both groups -7sole and del(7q)sole and a minimum of 23 total mutations in the extension cohort (frequency of ≥5%).
2. Odds ratio plot for the results from a multivariable binomial logistic regression fitted for all genes that showed a tendency (with a P <.1) in the univariable fitting. The bar plot on the left depicts the number of samples included in the test for the four genes included in the multivariate model.

On the right side, logOR is given with a confidence interval of 95%, CI, and P-value.


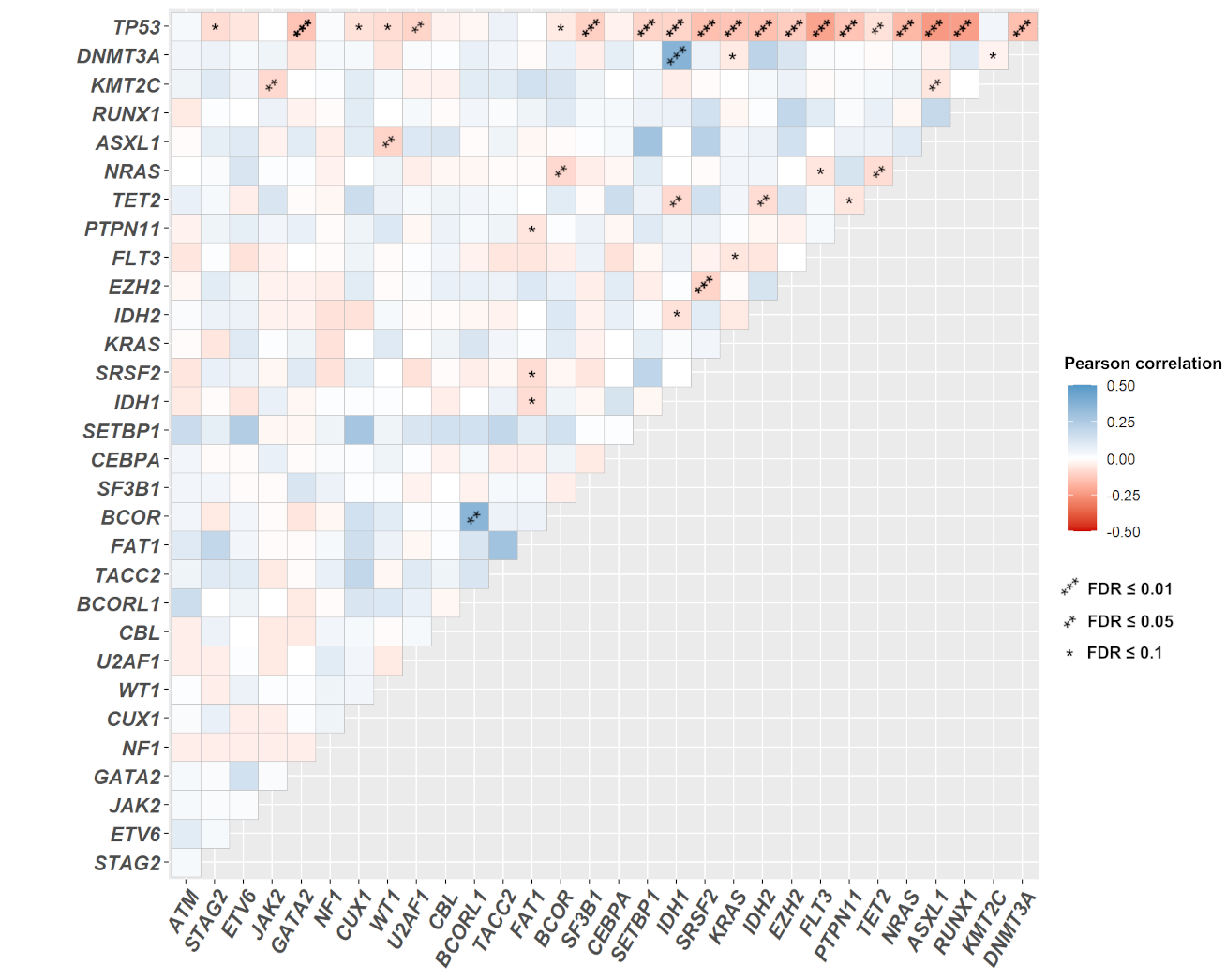


**Figure S10. Co-occurrence and mutual exclusivity patterns of mutations in the abn(7) cohort.**

Pearson's correlation plot for the mutations found in 467 patients of the extension cohort (blue shows different levels of co-mutation, and red shows various levels of mutual exclusivity). Asterisks highlight boxes to display the significant pairs of co-mutations and mutual exclusivity resulting from the application of the pairwise function from the discover algorithm^24^ to the genes present in more than 3% of the cohort (genes n=31, 465 pairs tested, FDR estimation method: discrete Benjamini-Hochberg, significance levels: * FDR ≤.1; ** FDR ≤.05; *** FDR ≤.01).

**A**
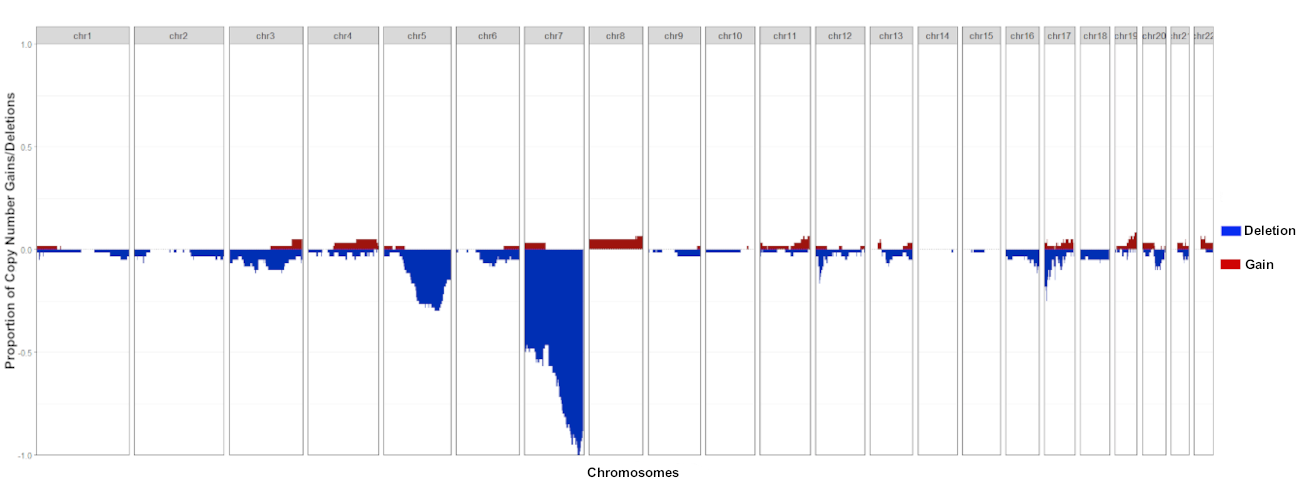


**B**


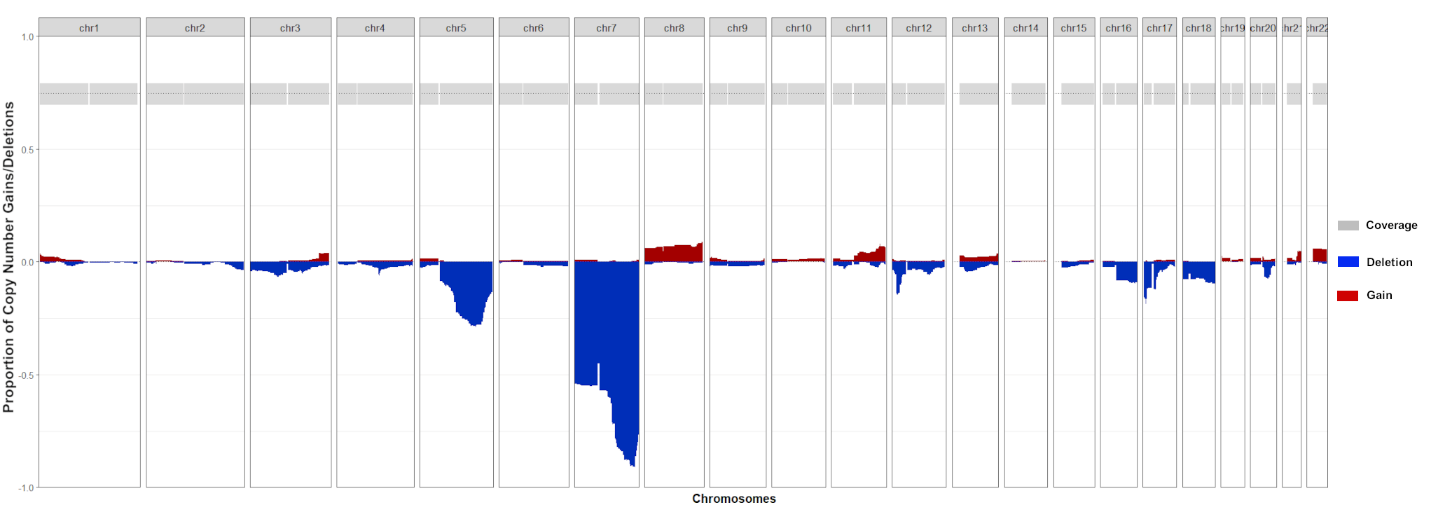


**Figure S11. Comparison of CNV patterns detected from WES and from TS.**

1. Distribution of CNVs across the autosomes, reporting deletions (blue) and gains (red) found in the exploration cohort with WES of paired diagnosis/CR samples. The proportion of affected patients per position was derived from WES data from the exploration cohort (n=60).
2. Distribution of CNVs reporting deletions (blue) and gains (red) found in the extension cohort across the autosomes. The proportion of affected patients per position was derived from TS data for the SNP-backbone of the extension cohort (n=342). Coverage obtained using the *PureCN* segmentation algorithm is shown (grey), gains (red), and deletions (blue) were manually curated after the cut-offs for log(CN) values of >0.4 and < -0.3, respectively.

**A**


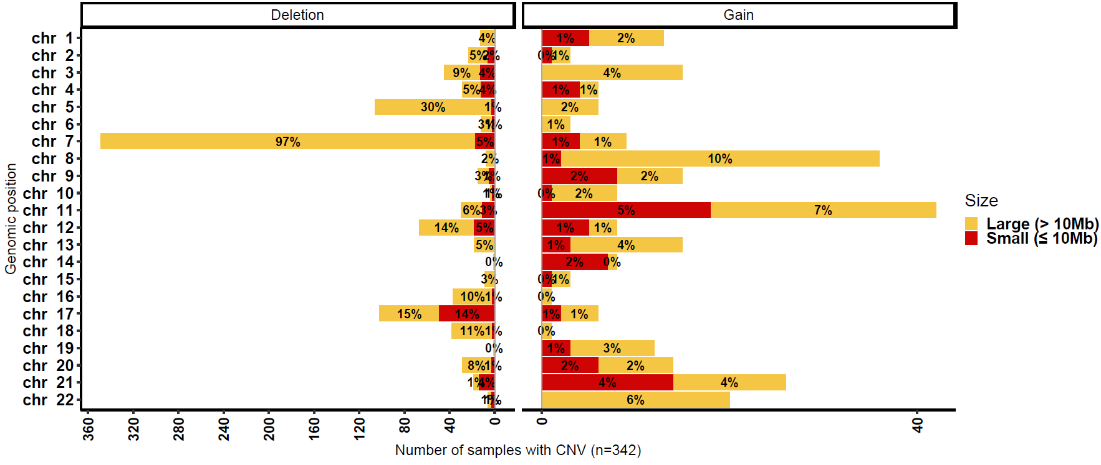


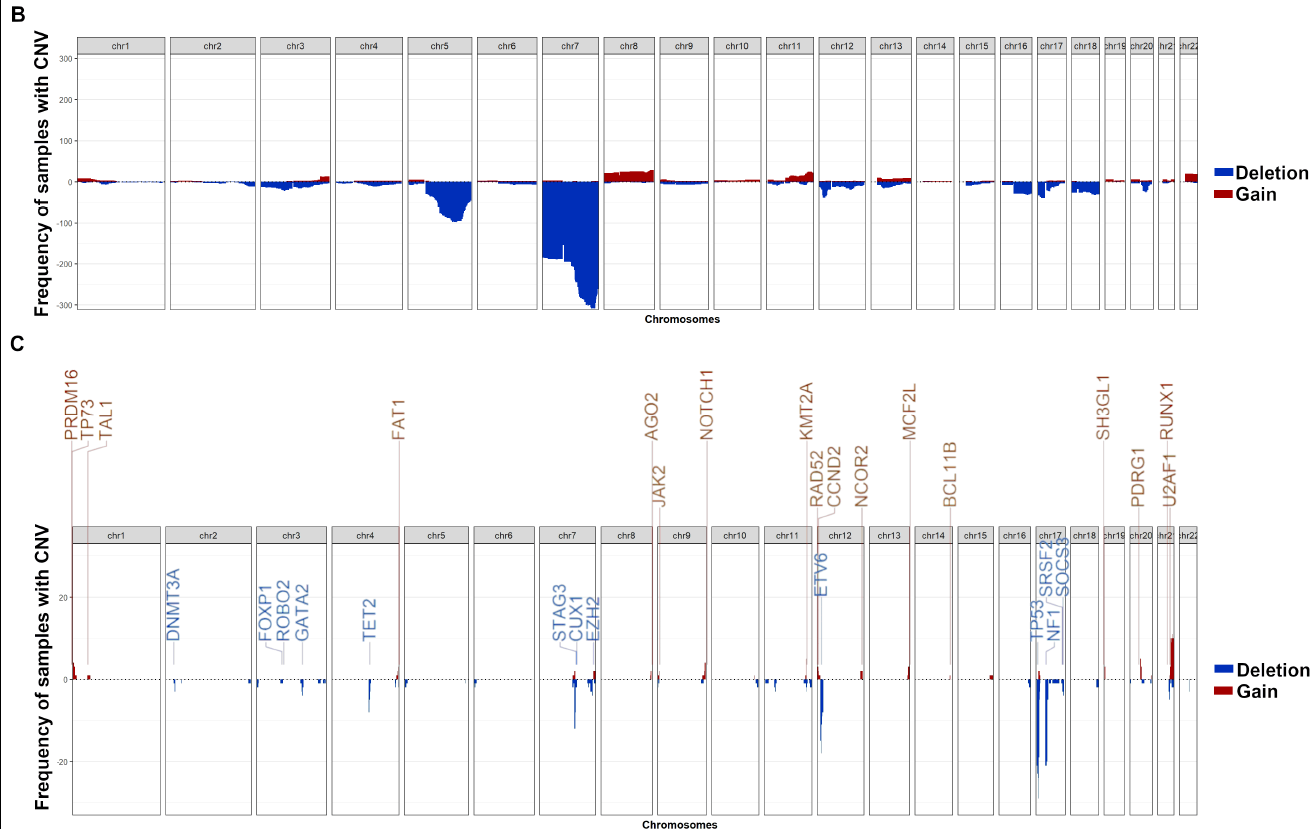


**Figure S12. Frequency and genomic positions of large and small CNVs in the abn(7) cohort.**

1. Distribution of large (>10Mb) and small (≤10Mb) CNVs across chromosomes. The percentage given in the bar plot relates to patients in the extension cohort with available CNV data (n=342).
2. Frequency of large (>10Mb) gains (red) and deletions (blue) across genomic positions affecting 340 of 342 patients.
3. Frequency of small (≤10Mb) gains (red) and deletions (blue) across genomic positions affecting 264 of 342 patients. Genes of interest in leukemia at these genomic positions are indicated.

| **A** | 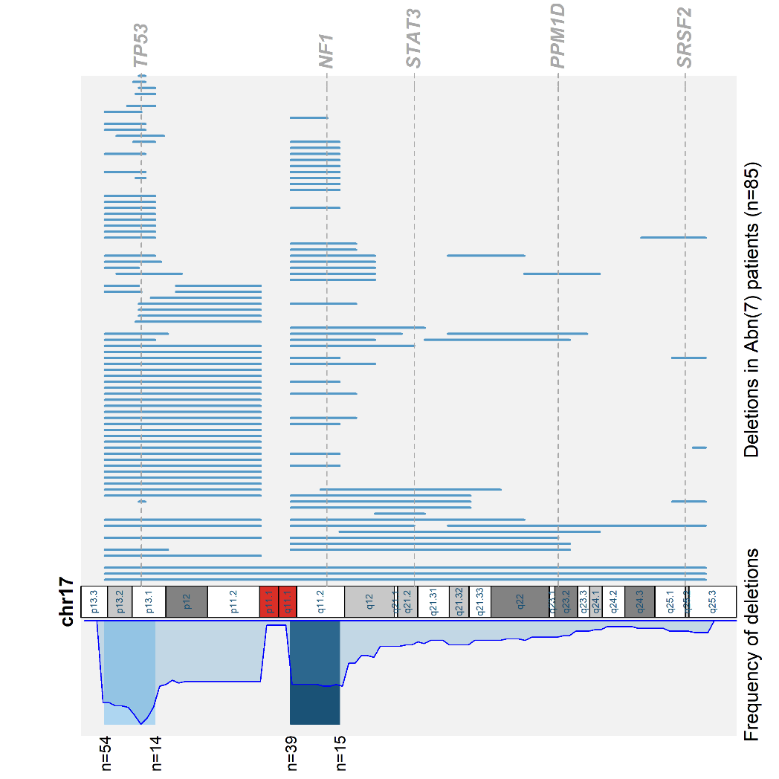 |
| --- | --- |
| **B** | 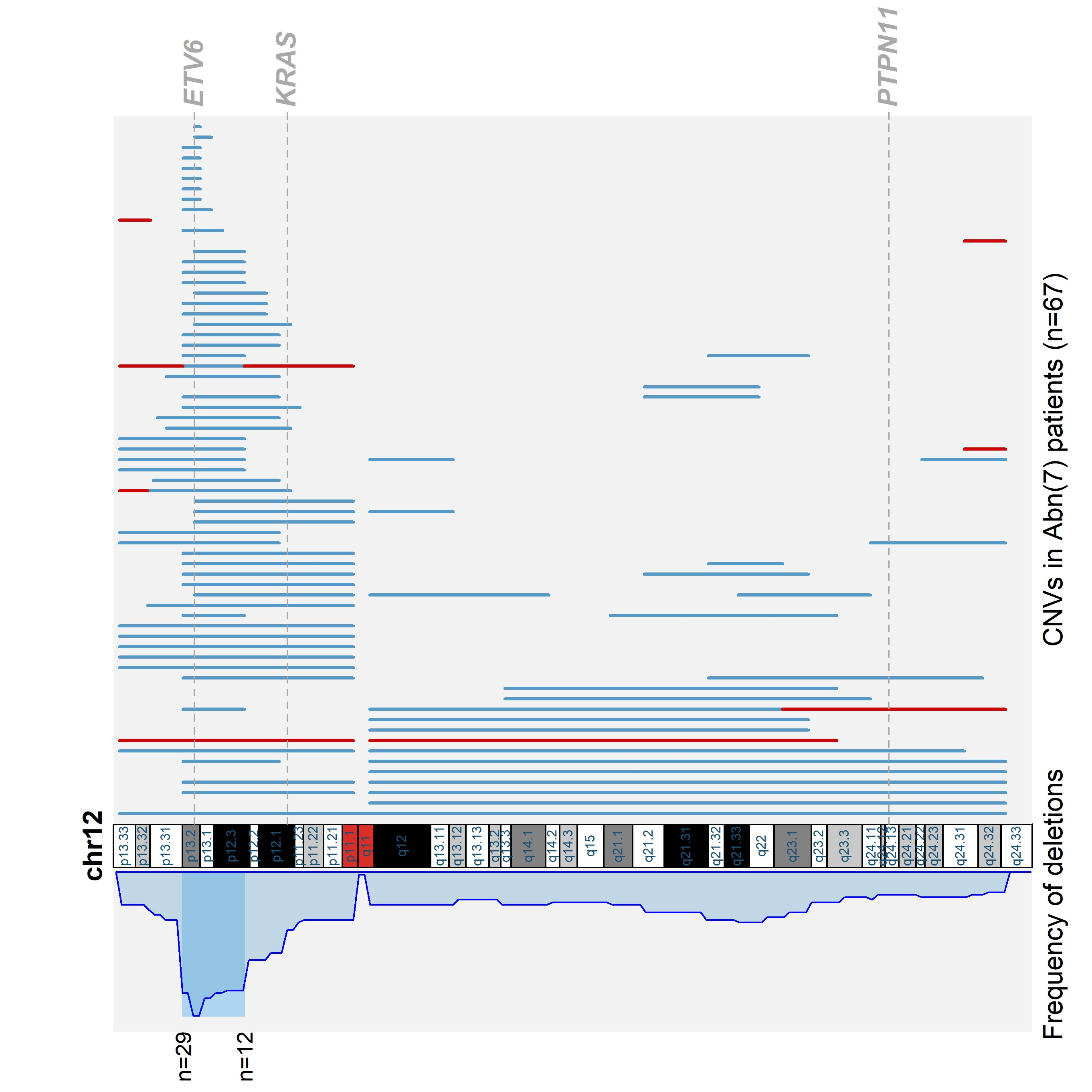 |

**Figure S13. Three main CDRs in chromosomes 17 and 12.**

Graphs show the genomic distributions of CNVs per sample. On the right side of the graphs, (A) deletions of chr17 are marked (blue), (B) gains and deletions of chr12 are shown (red and blue respectively). On the left side of each chromosome, densities of deletions across the chromosome are displayed. The most affected regions (CDRs) are colored in shades of blue, and n marks the number of occurrences of these breakpoints.

**A**


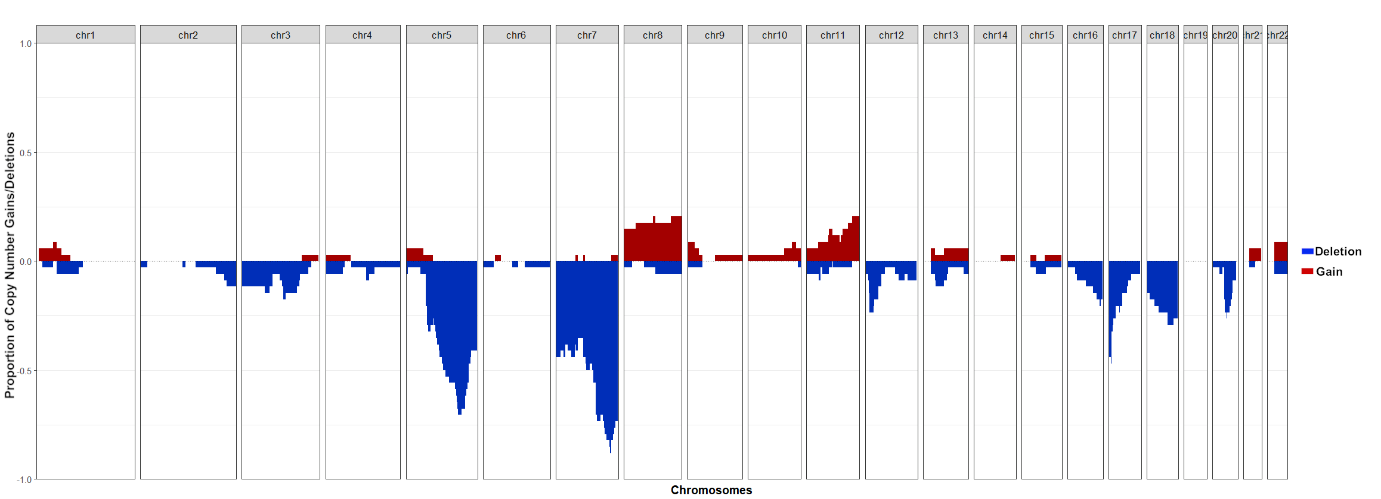


**B**
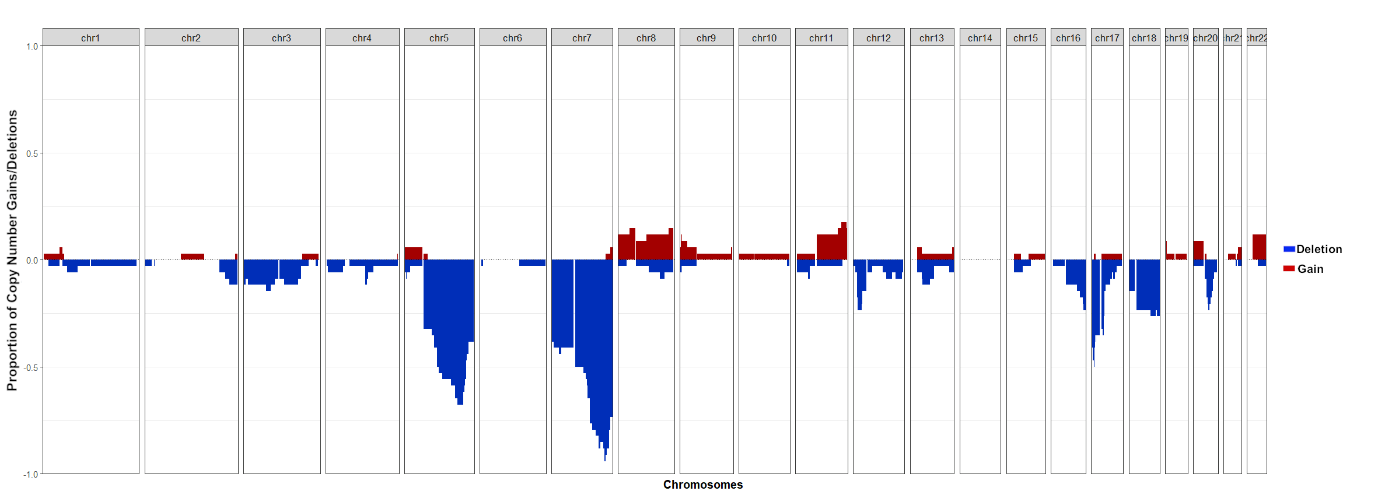


**Figure S14. Comparison of CNV profiles obtained with the TS approach and Oxford Nanopore sequencing.**

The graph shows the proportion of patients affected by a CNV, distributed per chromosomal position of the CNVs. It reports deletions (blue) and gains (red) found in the extension cohort across the autosomes for the same 33 samples sequenced by two different technologies: (A) For Oxford Nanopore sequencing, a CNV was considered gain with log(CN)>0.3 and deletion with log(CN)<-0.2, after curation of 100Kb, 500Kb and 1000Kb segmentation data. (B) For the data derived from TS of the SNP-backbone, a CNV was considered a gain with log(CN)>0.4 and a deletion with log(CN)<-0.3. TS and Nanopore agreements were nearly perfect (K=.87, Table S2). Some minor disagreements in the TS data could be confirmed to be expected from karyotype information and/or were expected from the WES CNV profile (e.g., gains in chromosomes 19 and 20).


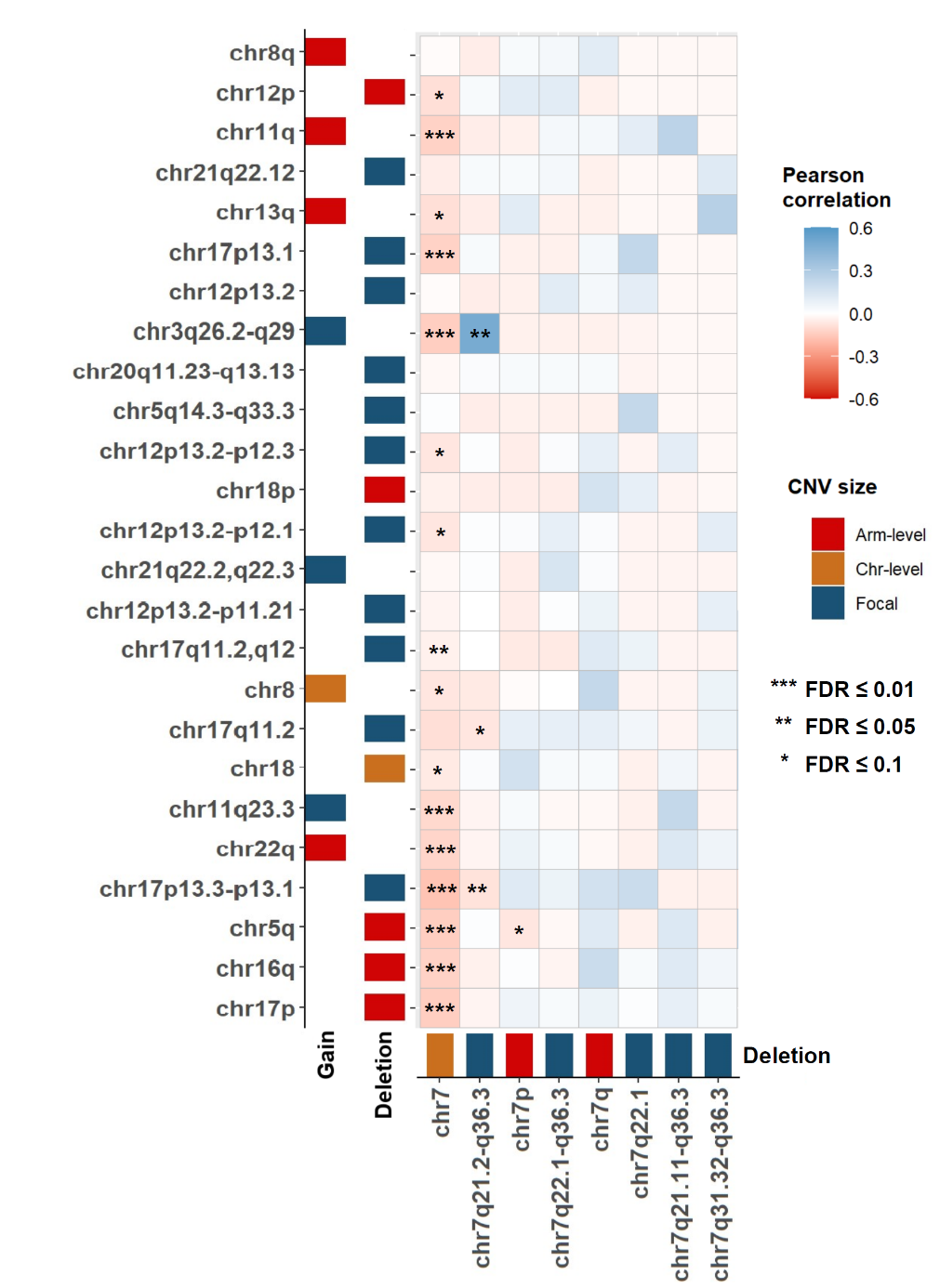


**Figure S15. Co-occurrence and mutual exclusivity patterns of chromosome 7 CDRs with other CNVs.**

Pearson's correlation for the CNVs using the TS of a SNP-backbone for the extension cohort (n=342 patients) according to their cytogenetic locations. In the gradient, blue shows different levels of co-mutation, and red shows different levels of mutual exclusivity. Overlaid with information for the significant pairs of co-occurrence and mutual exclusivity resulting from the application of the pairwise function from the discover algorithm^24^ to the segments present in more than seven patients of the cohort. CNV events are classified as chromosome/arm level and focal (n=33 different segments categorized at cytogenetic band level, 528 pairs tested, FDR estimation method: discrete Benjamini-Hochberg, significance levels: * FDR ≤.1; ** FDR ≤.05; *** FDR ≤.01).


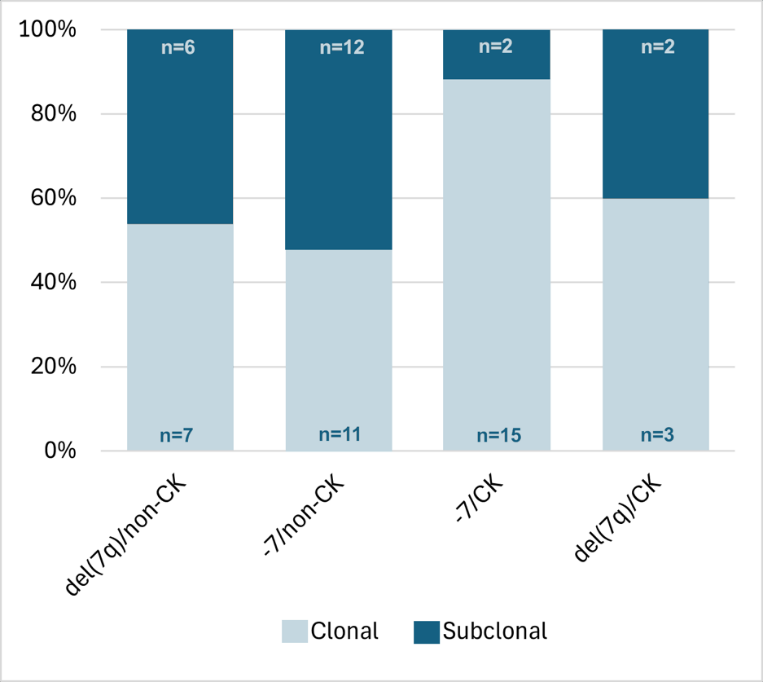


**Figure S16.** **Clonality distribution of -7 or del(7q) in the abn(7) groups segregated by karyotype.**

Bar graph showing the clonality distribution of -7 or del(7q) in the four main abn(7) groups, which is based on the CCF analysis in the WES exploration cohort of n=60 patients.


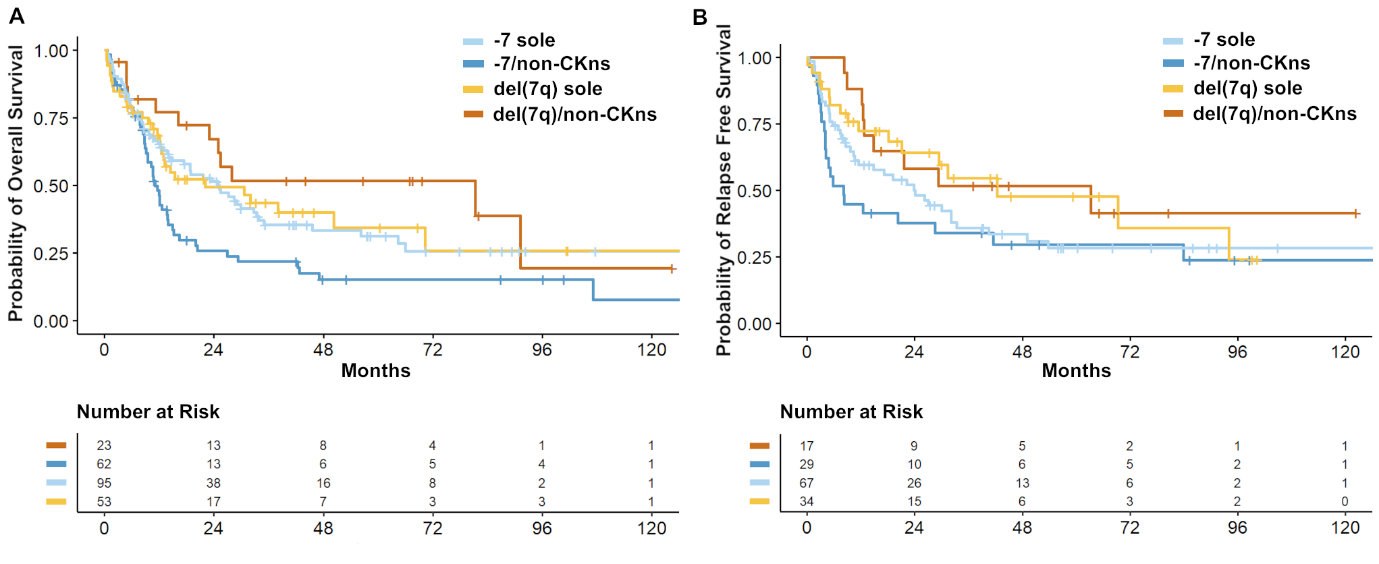


**Figure S17. Kaplan-Meier Curves for abn(7) non-CK groups**

Kaplan-Meier curves showing the probability of (A) OS (n=414) and (B) RFS (n=147) for intensively treated patients according to abn(7) / non-CK groups. Pairwise comparison LogRank Tests. (A) The only significant pairs were -7/non-CKns vs. del(7q)/non-CKns and -7sole vs. -7/non-CKns, both P=.042, ns = not sole. (B) P-values not significant in all groups.

**
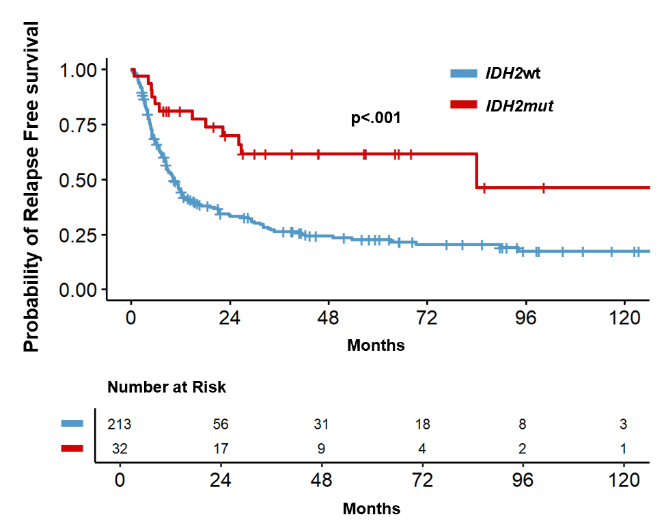

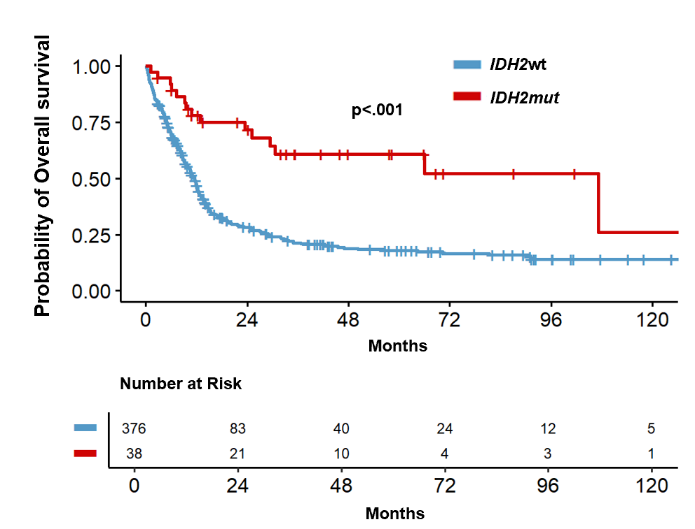

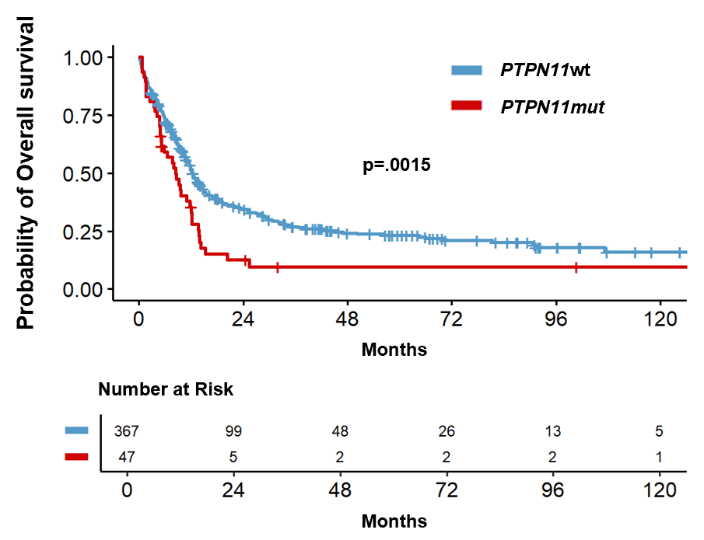

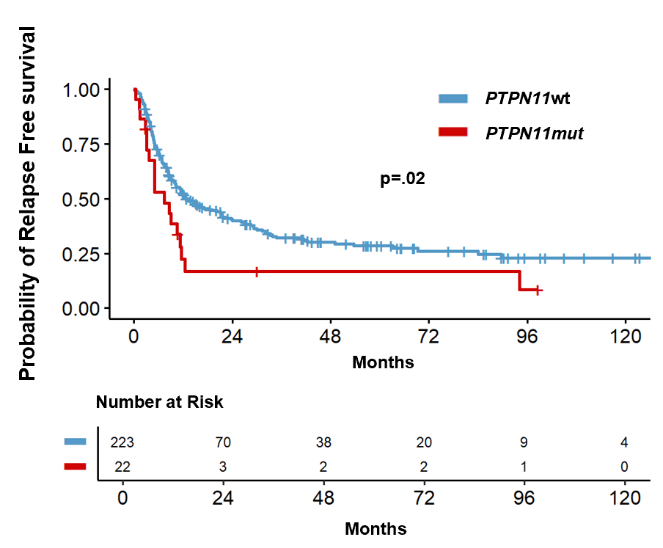
A B**

**C D**

**Figure S18.** **Kaplan Meier curves for abn(7) patients according to their *IDH2* and *PTPN11* mutation status.**

(A,B) Kaplan Meier curves chowing the probability of (A) OS of n=414 and (B) n=245 intensively treated patients according to their *PTPN11* mutation status.

(C,D) Kaplan Meier curves chowing the probability of (A) OS of n=414 and (B) n=245 intensively treated patients according to their *IDH2* mutation status.


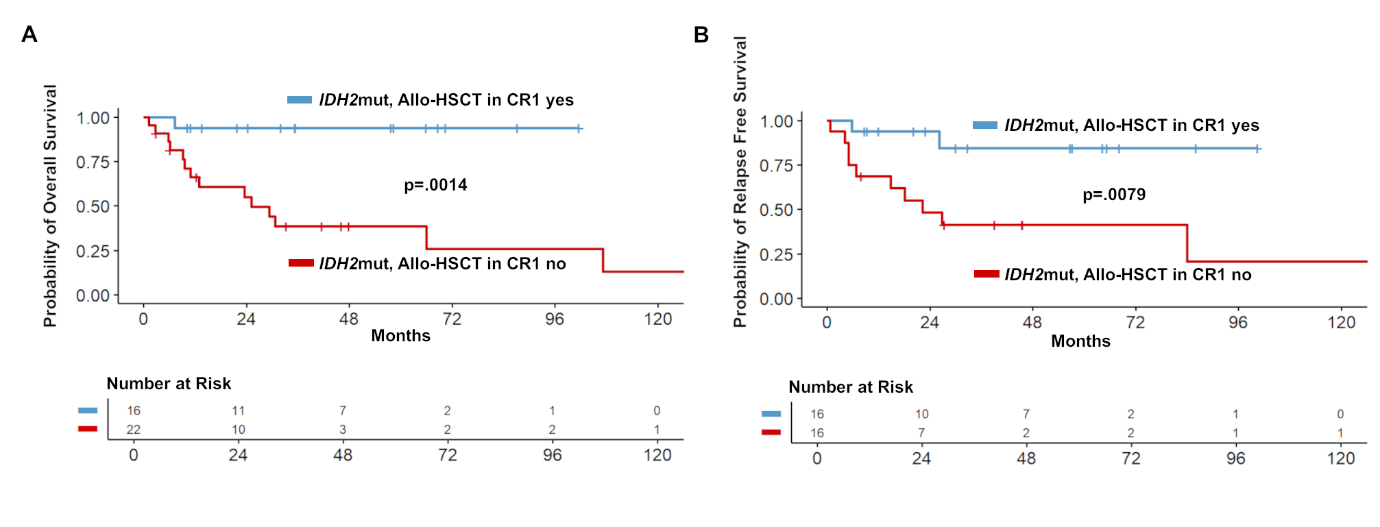


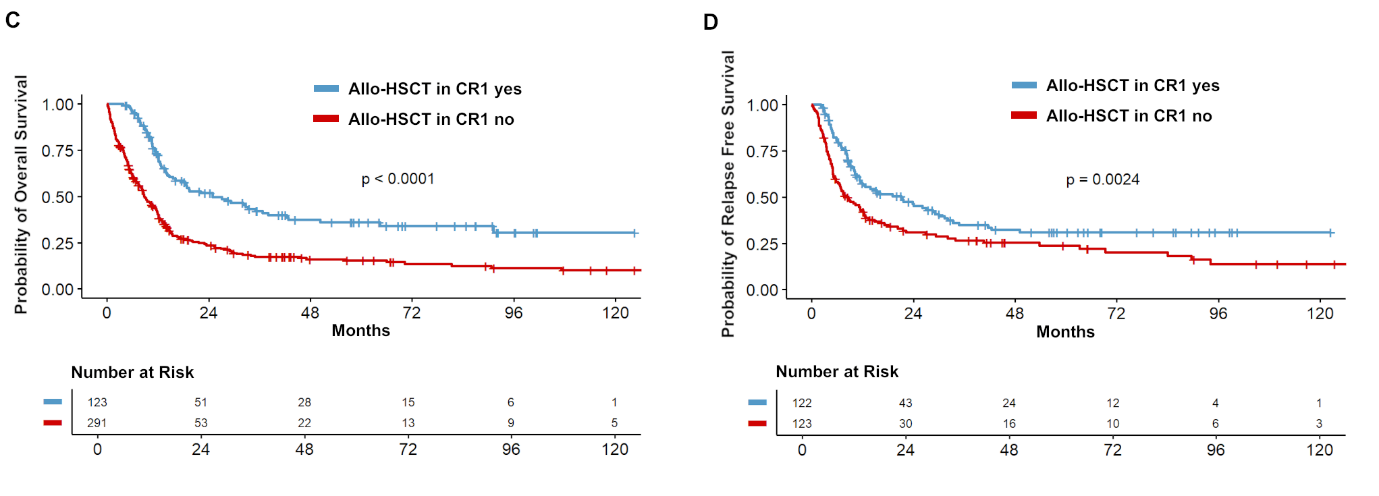


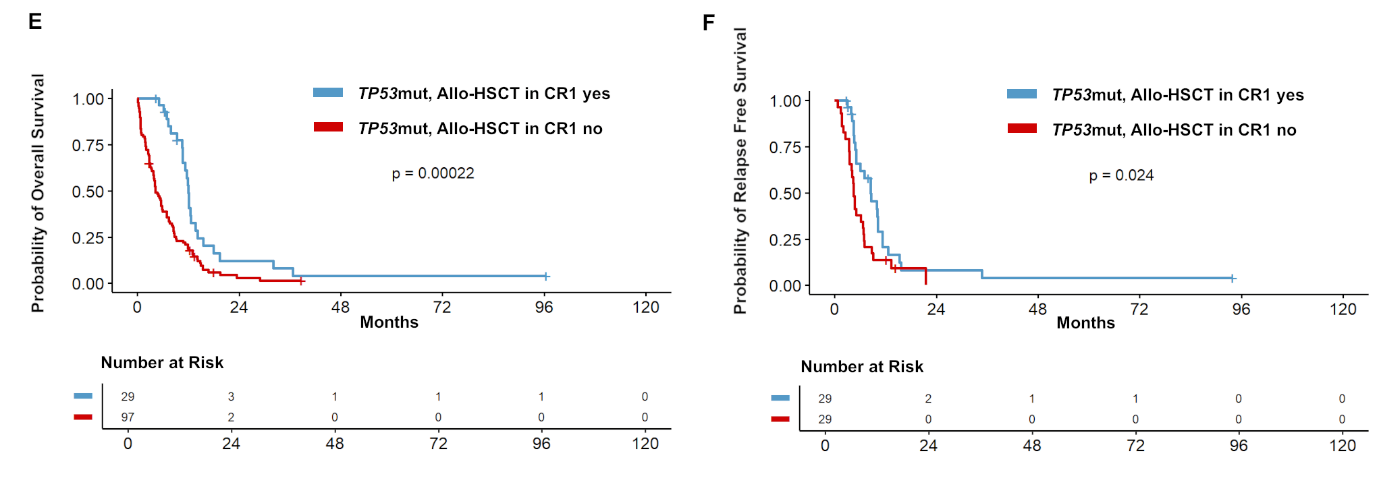


**Figure S19. Kaplan-Meier curves for patients according to their allogeneic transplantation status.**

(A,B) Kaplan-Meier Curves showing the probability of (A) OS (n=38) and (B) RFS (n=32) in intensively treated *IDH2*mut patients according to the performance of allogeneic stem cell transplantation (Allo-HSCT) in CR1 (yes vs. no). "Allo-HSCT in CR1 no" includes patients either without Allo-HSCT or with Allo-HSCT as a salvage therapy for refractory/relapsed disease.

(C,D) Kaplan-Meier Curves showing the probability of (C) OS (n=414) and (D) RFS (n=245) in intensively treated patients according to the performance of allogeneic stem cell transplantation (Allo-HSCT) in CR1 (yes vs. no). "Allo-HSCT in CR1 no" includes patients either without Allo-HSCT or with Allo-HSCT as a salvage therapy for refractory/relapsed disease.

(E,F) Kaplan-Meier Curves showing the probability of (E) OS (n=126) and (F) RFS (n=58) in intensively treated *TP53*mut patients according to the performance of allogeneic stem cell transplantation (Allo-HSCT) in CR1 (yes vs. no). "Allo-HSCT in CR1 no" includes patients either without Allo-HSCT or with Allo-HSCT as a salvage therapy for refractory/relapsed disease.


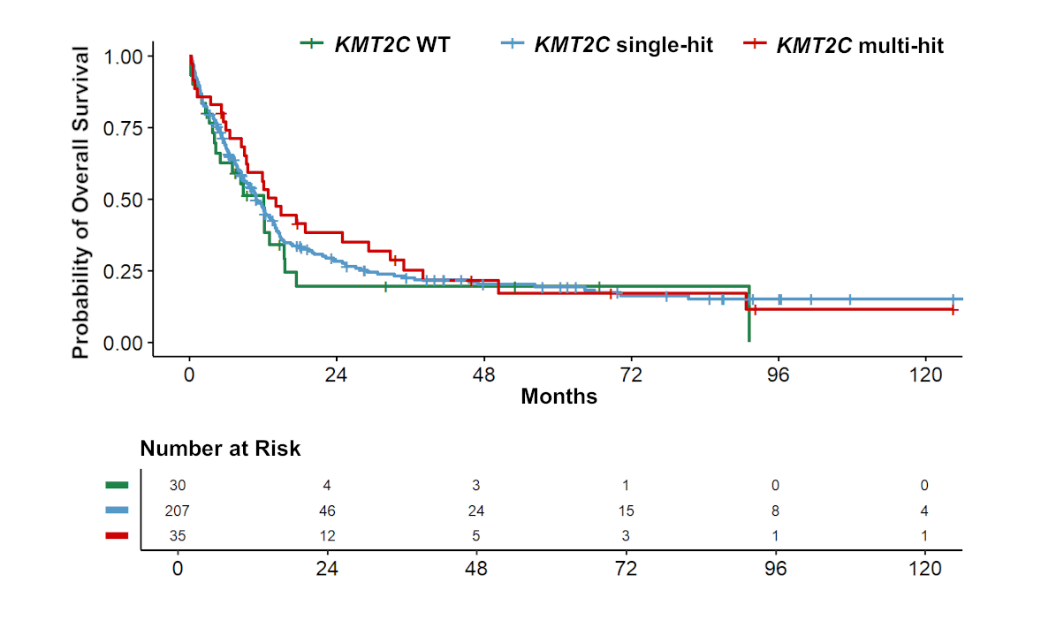


**Figure S20. Overall survival for abn(7) patients according to their *KMT2C* allelic status.**

Kaplan-Meier curve showing the probability of OS for the multi-hit groups affecting the *KMT2C* locus (n=272, patients intensively treated from the extension cohort with CNV data, LogRank Test, P= .58).


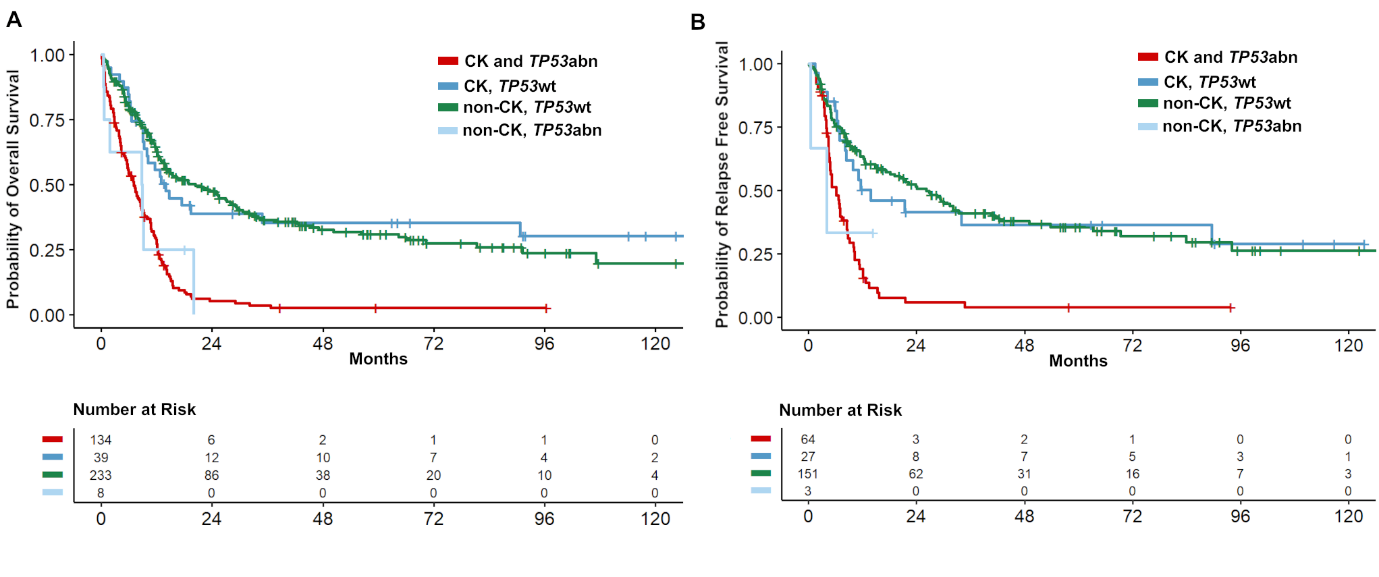


**Figure S21. Kaplan-Meier curves for abn(7) patients according to their CK and *TP53*abn status.**

Kaplan-Meier Curves showing the probability of (A) OS (n=414) and (B) RFS (n=245) in intensively treated abn(7) patients according to CK and *TP53* status. (A) Pairwise comparison LogRank Tests: CK and *TP53*abn vs. non-CK, *TP53*abn P= .85; non-CK, *TP53*wt vs. CK, *TP53*wt P= .89; non-CK, *TP53*abn vs. CK, *TP53*wt P= .071, all others P< .001. (B) Pairwise comparison LogRank Tests: non-CK, *TP53*wt vs. CK, *TP53*wt P= .82; non-CK, *TP53*wt and CK, *TP53*wt vs. CK and *TP53*abn P< .001.


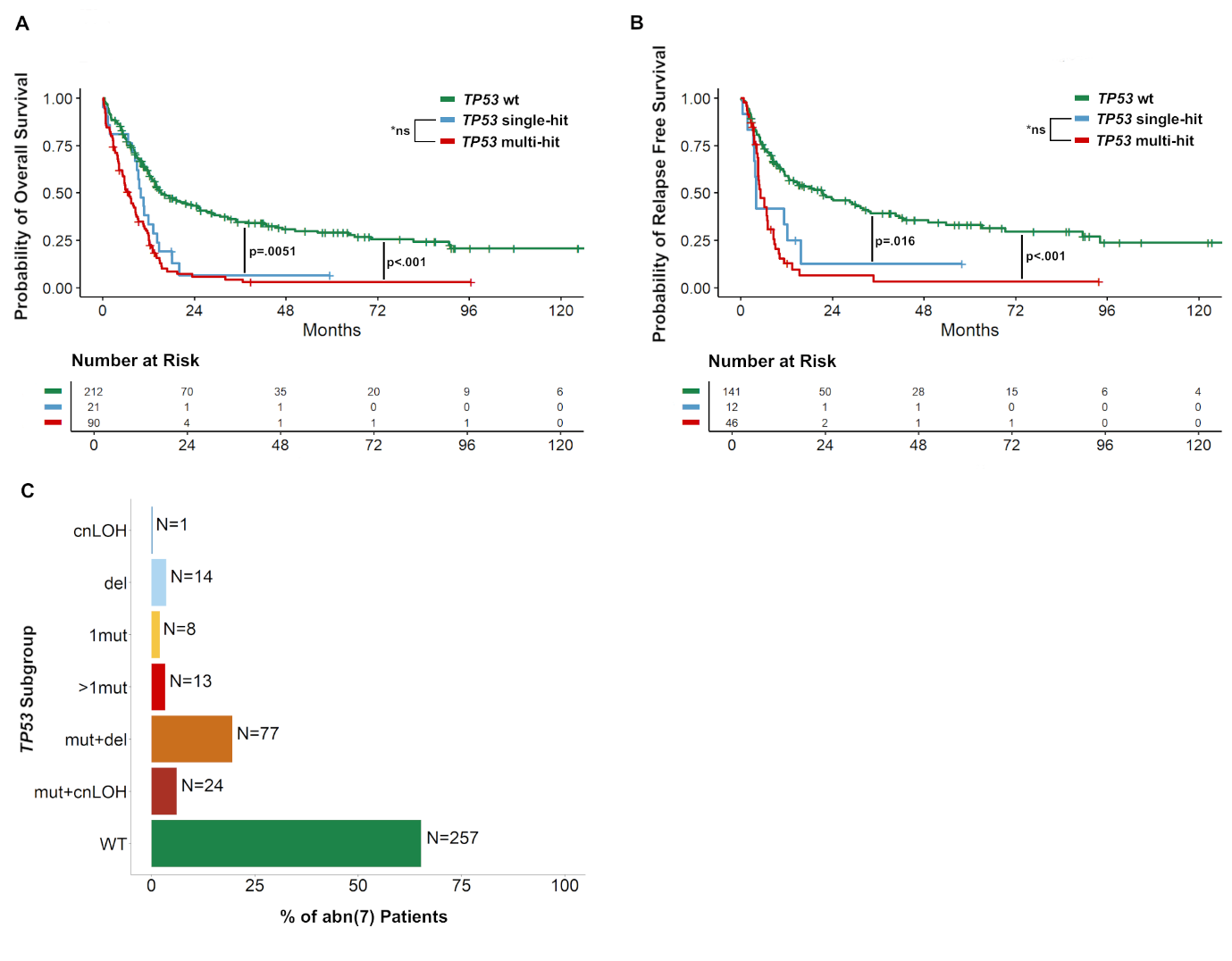


**Figure S22. *TP53* allelic status distribution and survival in abn(7) cohort.**

(A,B) Kaplan-Meier Curve showing the probability of (A) OS (n=323) and (B) RFS (n=199) in intensively treated patients compared according to their *TP53* allelic status (wt, single- or multi-hit, based on ICC classification ^26^). P-values from Pairwise LogRank Test, *ns = not significant, (A) *TP53* single-hit vs. *TP53* multi-hit, P =.17. (B) *TP53* single-hit vs. *TP53* multi-hit, P =.46.

(C) Bar Plot showing the distinct *TP53* allelic status subgroups with frequency (%) of patients (x-axis) for n=394 patients with available CNV data. Groups: >1mut, mut+del, and mut+cnLOH = multi-hit; del, 1mut, and cnLOH = single-hit, wt = wild type, according to ICC classification ^26^.

# Supplemental Tables

**Table S1. List of 66 genes in Customized Gene Panel for Targeted Sequencing**

| **Gene** | **Category** | **Exons** |  | **Gene** | **Category** | **Exons** |
| --- | --- | --- | --- | --- | --- | --- |
| ***ASXL1*** | Chromatin modifier | 1,2, 4-13 |  | ***MN1*** | Transcription regulator | 2 |
| ***ATM*** | DNA repair | 2-63 |  | ***MPL*** | Signaling | 10 |
| ***BCL11B*** | Transcription regulator | 1-4 |  | ***MT1G*** | Other | 1-3 |
| ***BCOR*** | Chromatin modifier | 2-15 |  | ***MYD88*** | Signaling | 1-5 |
| ***BCORL1*** | Chromatin modifier | 2-13 |  | ***NF1*** | Signaling | 28-38 |
| ***BRAF*** | Signaling | 16 |  | ***NOTCH1*** | Signaling | 26, 27, 34 |
| ***BRCC3*** | DNA repair | 1-11 |  | ***NPM1*** | Other | 11 |
| ***BRINP3*** | Other | 2-8 |  | ***NRAS*** | Signaling | 2-5 |
| ***CALR*** | Other | 8+9 |  | ***PHF6*** | Other | 4-6, 8-10 |
| ***CBL*** | Signaling | 1-16 |  | ***PPM1D*** | DNA repair | 1-6 |
| ***CEBPA*** | Transcription regulator | 1 |  | ***PTPN11*** | Signaling | 1-15 |
| ***CHEK2*** | DNA repair | 2-16 |  | ***RAD21*** | Cohesin complex | 2-14 |
| ***CSF3R*** | Other | 14+17 |  | ***ROBO2*** | Other | 1-26 |
| ***CUX1*** | Other | 1-24 |  | ***RUNX1*** | Transcription regulator | 1-8 |
| ***CXCR4*** | Other | 1+2 |  | ***SAMD9*** | Other | 3 |
| ***DHX15*** | Splicing | 1-14 |  | ***SAMD9L*** | Other | 5 |
| ***DNMT3A*** | DNA methylation | 2-23 |  | ***SETBP1*** | DNA methylation | 4 |
| ***ETV6*** | Transcription regulator | 1-8 |  | ***SF1*** | Splicing | 1-14 |
| ***EZH2*** | Chromatin modifier | 2-20 |  | ***SF3B1*** | Splicing | 1-25 |
| ***FAT1*** | Other | 2-29 |  | ***SMARCA2*** | Chromatin modifier | 2-33 |
| ***FLT3*** | Signaling | 6,14,15,20 |  | ***SRSF2*** | Splicing | 1+2 |
| ***GATA1*** | Transcription regulator | 2 |  | ***STAG2*** | Cohesin complex | 3-35 |
| ***GATA2*** | Transcription regulator | 2-6 |  | ***STAT3*** | Transcription regulator | 2-24 |
| ***GNAS*** | Signaling | 1-13 |  | ***TACC2*** | Other | 1-22 |
| ***GNB1*** | Signaling | 3-11 |  | ***TET2*** | DNA methylation | 3-11 |
| ***IDH1*** | DNA methylation | 3-10 |  | ***TP53*** | DNA repair | 2-11 |
| ***IDH2*** | DNA methylation | 1-11 |  | ***U2AF1*** | Splicing | 1-8 |
| ***JAK2*** | Signaling | 3-25 |  | ***WT1*** | Other | 1-10 |
| ***KIT*** | Signaling | 8-11, 17 |  | ***XPO1*** | Other | 15 |
| ***KMT2C*** | DNA methylation | 1-59 |  | ***ZBTB7A*** | Transcription regulator | 2+3 |
| ***KRAS*** | Signaling | 2-5 |  | ***ZFP36L2*** | Transcription regulator | 1+2 |
| ***LUC7L2*** | Splicing | 2 |  | ***ZNF217*** | Transcription regulator | 1-4 |
| ***MEIS2*** | Transcription regulator | 1-12 |  | ***ZRSR2*** | Splicing | 1-11 |

**Table S2. Distribution of known AML translocations according to abn(7) groups and *KMT2C* mutation status.**

| **Known AML translocations** | **Recurrent Fusions** | **n** | **-7/**  **CK,**  **n = 136** | **-7/**  **non-CK,**  **n = 192** | **del(7q)/**  **CK,**  **n= 70** | **del(7q)/**  **non-CK,**  **n = 92** | ***KMT2C***  **mut,**  **n = 82** | ***KMT2C***  **wt,**  **n = 437** |
| --- | --- | --- | --- | --- | --- | --- | --- | --- |
| inv(3)(q21.3q26.2)/  t(3;3) | MECOM(EVI1) rearrangement | 27 | 6 (4.5%) | 19 (10%) | 1 (1.5%) | 1 (1.1%) | 4 (5.1%) | 23 (5.4%) |
| *Missing* |  |  | *2* | *5* | *2* | *3* | *3* | *9* |
| inv(16)(p13q22)/  t(16;16) | CBFB::MYH11 | 8 | 0 (0%) | 0 (0%) | 2 (2.9%) | 5 (5.6%) | 3 (3.8%) | 5 (1.2%) |
| *missing* |  |  | *3* | *5* | *2* | *3* | *3* | *10* |
| t(8;21)(q22;q22) | RUNX1::RUNX1T | 3 | 0 (0%) | 1 (0.5%) | 2 (2.9%) | 0 (0%) | 2 (2.5%) | 1 (0.2%) |
| *missing* |  |  | *2* | *5* | *2* | *3* | *3* | *9* |
| t(9;22)(q34;q11) | BCR::ABL1 | 3 | 0 (0%) | 3 (1.6%) | 0 (0%) | 0 (0%) | 1 (1.3%) | 2 (0.5%) |
| *missing* |  |  | *2* | *5* | *2* | *3* | *3* | *9* |
| t(3;5) (q22∼q25;q32∼q35) | NPM::MLF1 | 2 | 1 (0.8%) | 0 (0%) | 0 (0%) | 0 (0%) | 0 (0%) | 2 (0.5%) |
| *missing* |  |  | *3* | *5* | *2* | *3* | *3* | *10* |
| t(9;11)(p21-22;q23) | MLLT3::MLL | 3 | 2 (1.5%) | 1 (0.5%) | 0 (0%) | 0 (0%) | 1 (1.3%) | 2 (0.5%) |
| *missing* |  |  | *3* | *5* | *2* | *3* | *3* | *10* |
| t(15;17)(q22;q21) | PML::RARA | 2 | 0 (0%) | 0 (0%) | 1 (1.5%) | 1 (1.1%) | 0 (0%) | 2 (0.5%) |
| *missing* |  |  | *2* | *5* | *2* | *3* | *3* | *9* |

**Table S3. Agreements between karyotype frequent events and results from SNP-backbone TS and Nanopore data.**


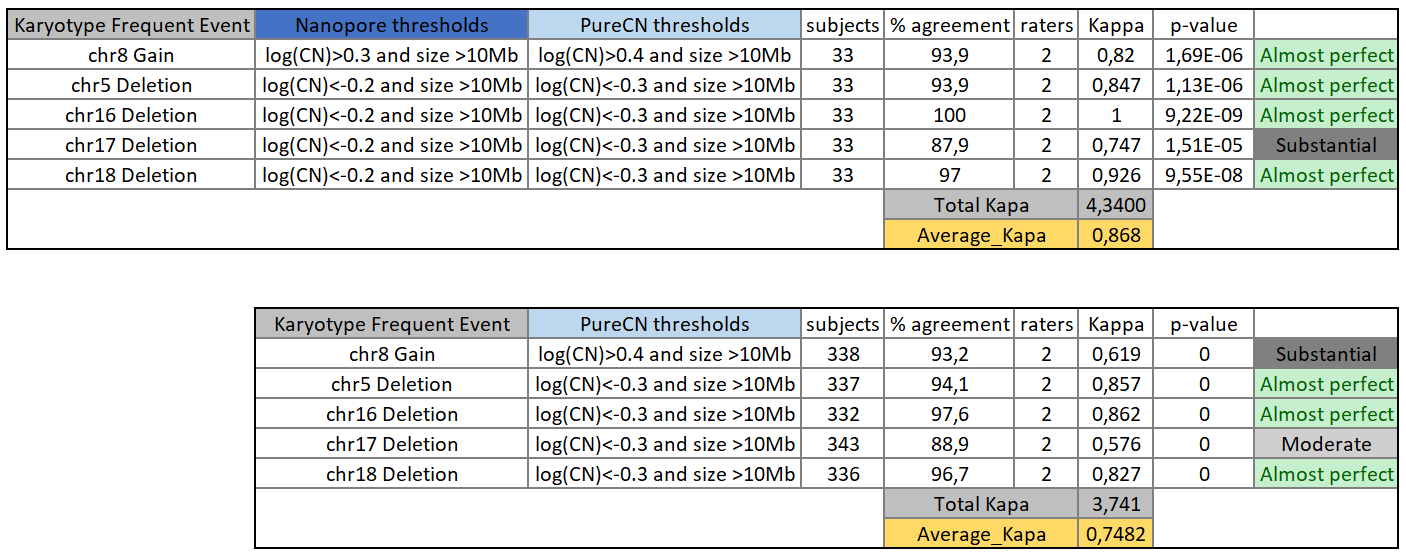


**Table S4. Estimations of proportions of CNVs in the extension cohort challenging to detect by G-banding assays.**

The table gives the number of patients and the % of abn(7) for genes of interest (relative to 342 patients). The probability of detection considers a limit of detection for G-banding ranging from 10Mb to 15Mb (depending on the resolution of preparations and microscopes in given centers). It reports a range for the proportions (Prop.) of CNVs at each gene that are below the resolutions of 10Mb to 15Mb. The table is ordered by a descendent number of patients with a small CNV(≤10Mb).

**Table S5. Estimations of proportions of deletions in genes of the chr7**

The table shows the number of patients and the % of abn(7), relative to 342 patients in the extension cohort, for genes of interest in the 7q-arm that suffer from recurrent deletions. The probability of detection considers a limit of detection for G-banding ranging from 10Mb to 15Mb (depending on the resolution of preparations and microscopes in given centres). The table reports a range for the proportions of CNVs at each gene that are below the resolutions of 10Mb to 15Mb.

**Table S6. Predictors of CR achievement**

The table shows the multivariate model for the analysis of CR. In the Logistic regression analysis, genomic events (gene mutations and cytogenetic aberrations) were taken as variables and potential clinical confounders as covariates. The intensively treated patients with CR information of the abn(7) cohort were considered (n=394). For clinical continuous variables, a separation into two groups was determined by the median value of the whole cohort (Table 1, age, 59 years old and WBC 9U/nL. Genomic events were included in the multivariate logistic regression analysis if they were detected in the >5% of patients and had a univariate P ≤.1 for CR before adjustments for multiple comparisons. Cytogenetic aberrations like -7 (Monosomy 7), del(7q), other monosomies, and complex karyotype (CK) are retrieved from clinical information. The odds ratio is given as OR (95% of CI) with P-values.


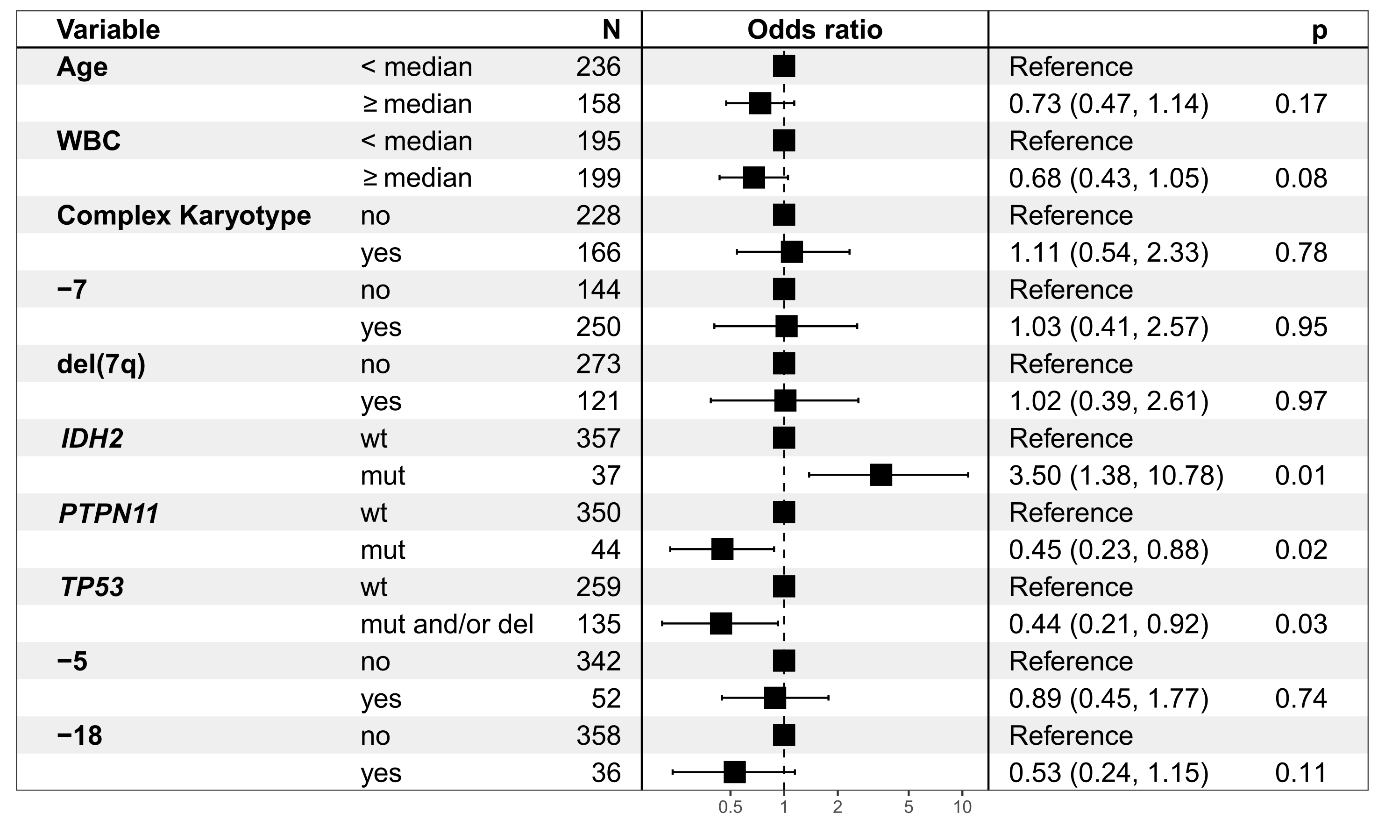


**Table S7. Clinical and genetic characteristics of patients according to *KMT2C* mutation status (mut vs. wt) in the extension cohort.**

The table shows the characteristics of the abn(7) patients grouped by the absence (wt) or presence of a mutation of *KMT2C* detected by TS. The groups include only the intensively treated patients (n=364). Median groups are according to median values of Table 1.

|  | | ***KMT2C* status (mut vs. wt)** | |  |
| --- | --- | --- | --- | --- |
| **Variable** | **N** | **mut**, n = 63^1^ | **wt**, n = 301^1^ | **P-value**^2^ |
| **Age (median, IQR)** | 364 | 55 (41, 64) | 55 (45, 64) | 0.6 |
| **Sex** | 364 |  |  | 0.8 |
| f |  | 29 (46%) | 133 (44%) |  |
| m |  | 34 (54%) | 168 (56%) |  |
| **AML type** | 363 |  |  | 0.5 |
| *de novo* AML |  | 54 (86%) | 238 (79%) |  |
| sAML |  | 5 (7.9%) | 39 (13%) |  |
| tAML |  | 4 (6.3%) | 23 (7.7%) |  |
| *missing data* |  | *0* | *1* |  |
| **FAB type** | 222 |  |  | 0.3 |
| M0 |  | 8 (20%) | 37 (20%) |  |
| M1 |  | 11 (28%) | 43 (24%) |  |
| M2 |  | 8 (20%) | 27 (15%) |  |
| M3 |  | 0 (0%) | 2 (1.1%) |  |
| M4 |  | 9 (23%) | 32 (18%) |  |
| M5 |  | 0 (0%) | 19 (10%) |  |
| M6 |  | 4 (10%) | 15 (8.2%) |  |
| M7 |  | 0 (0%) | 7 (3.8%) |  |
| *missing data* |  | *23* | *119* |  |
| **Abn(7) group** | 364 |  |  |  |
| -7/ non-CKns |  | 7 (11%) | 46 (15%) |  |
| -7 sole |  | 14 (22%) | 73 (24%) |  |
| del(7q)/ non-CKns |  | 6 (9.5%) | 15 (5.0%) |  |
| del(7q) sole  -7/ CK  del(7q)/ CK  other/ CK |  | 9 (14%)  13 (21%)  13 (21%)  0 | 36 (12%)  75 (25%)  34 (11%)  15 (5%) |  |
| other/ non-CK |  | 1 (1.6%) | 7 (2.3%) |  |
| **Complex Karyotype** | 364 |  |  | >0.9 |
| no |  | 37 (59%) | 177 (59%) |  |
| yes |  | 26 (41%) | 124 (41%) |  |
| **-7sole** | 364 |  |  | 0.7 |
| no |  | 49 (78%) | 228 (76%) |  |
| yes |  | 14 (22%) | 73 (24%) |  |
| **del(7q)sole** | 364 |  |  | 0.6 |
| no |  | 54 (86%) | 265 (88%) |  |
| yes |  | 9 (14%) | 36 (12%) |  |
| **WBC** | 355 |  |  | 0.3 |
| < median |  | 34 (55%) | 141 (48%) |  |
| ≥ median |  | 28 (45%) | 152 (52%) |  |
| *missing data* |  | *1* | *8* |  |
| **Blast count** | 334 |  |  | 0.2 |
| < median |  | 31 (53%) | 119 (43%) |  |
| ≥ median |  | 27 (47%) | 157 (57%) |  |
| *missing data* |  | *5* | *25* |  |
| **Platelets** | 327 |  |  | 0.081 |
| < median |  | 33 (58%) | 122 (45%) |  |
| ≥ median |  | 24 (42%) | 148 (55%) |  |
| *missing data* |  | *6* | *31* |  |
| **Complete Remission** | 362 |  |  | 0.4 |
| no |  | 25 (40%) | 135 (45%) |  |
| yes |  | 38 (60%) | 164 (55%) |  |
| *missing data* |  | *0* | *2* |  |
| **Allo-HSCT** | 330 |  |  | 0.7 |
| yes |  | 18 (32%) | 80 (29%) |  |
| no |  | 39 (68%) | 193 (71%) |  |
| *missing data* |  | *6* | *28* |  |
| ^1^Median (IQR); n (%) | | | | |
| ^2^Wilcoxon rank sum test; Pearson's Chi-squared test; Fisher's exact test | | | | |

**Table S8. Patient characteristics by AML Type**

The table shows the patient characteristics of all abn(7) patients with complete clinical information (n=518) grouped by AML type [*de novo*, secondary(s), and therapy-related (t) AML]. Median groups are according to median values of Table 1.

|  |  |  | **AML type** |  |  |
| --- | --- | --- | --- | --- | --- |
| **Patient characteristics** | **n** | ***de novo* AML**, n = 404^1^ | **sAML**, n = 75^1^ | **tAML**, n = 39^1^ | **P-value**^2^ |
| **Age (median, IQR)** | 518 | 58 (45, 67) | 68 (62, 75) | 54 (49, 63) | <.001 |
| **Sex** | 518 |  |  |  | .2 |
| f |  | 181 (45%) | 29 (39%) | 22 (56%) |  |
| m |  | 223 (55%) | 46 (61%) | 17 (44%) |  |
| **Abn(7) groups** | 518 |  |  |  |  |
| -7/ non-CKns |  | 56 (14%) | 4 (5.3%) | 6 (15%) |  |
| -7 sole |  | 84 (21%) | 31 (41%) | 10 (26%) |  |
| del(7q)/ non-CKns |  | 20 (5%) | 1 (1.3%) | 2 (5.1%) |  |
| del(7q) sole |  | 50 (12%) | 11 (15%) | 8 (21%) |  |
| -7/ CK |  | 119 (29%) | 10 (13%) | 7 (18%) |  |
| del(7q)/ CK |  | 58 (14%) | 8 (11%) | 4 (10%) |  |
| other/ CK |  | 10 (2.5%) | 8 (11%) | 1 (2.6%) |  |
| other/ non-CK |  | 7 (1.7%) | 2 (2.7%) | 1 (2.6%) |  |
| **Complex Karyotype** | 518 |  |  |  | .044 |
| no |  | 217 (54%) | 49 (65%) | 27 (69%) |  |
| yes |  | 187 (46%) | 26 (35%) | 12 (31%) |  |
| **-7sole** | 518 |  |  |  | <.001 |
| no |  | 320 (79%) | 44 (59%) | 29 (74%) |  |
| yes |  | 84 (21%) | 31 (44%) | 10 (26%) |  |
| **del(7q)sole** | 518 |  |  |  | .3 |
| no |  | 354 (88%) | 64 (85%) | 31 (79%) |  |
| yes |  | 50 (12%) | 11 (15%) | 8 (21%) |  |
| **WBC** | 504 |  |  |  | .6 |
| < median |  | 201 (51%) | 31 (45%) | 20 (53%) |  |
| ≥ median |  | 196 (49%) | 38 (55%) | 18 (47%) |  |
| *missing data* |  | *7* | *6* | *1* |  |
| **Blast Count** | 472 |  |  |  | .08 |
| < median |  | 176 (47%) | 37 (62%) | 19 (54%) |  |
| ≥ median |  | 201 (53%) | 23 (38%) | 16 (46%) |  |
| *missing data* |  | *27* | *15* | *4* |  |
| **Platelets** | 469 |  |  |  | .4 |
| < median |  | 182 (49%) | 34 (57%) | 17 (45%) |  |
| ≥ median |  | 189 (51%) | 26 (43%) | 21 (55%) |  |
| *missing data* |  | *33* | *15* | *1* |  |
| **Complete Remission** | 516 |  |  |  | .4 |
| no |  | 193 (48%) | 40 (53%) | 16 (41%) |  |
| yes |  | 209 (52%) | 35 (47%) | 23 (59%) |  |
| *missing data* |  | *2* | *0* | *0* |  |
| **Allo-HSCT in CR1** | 470 |  |  |  | .5 |
| yes |  | 102 (28%) | 17 (25%) | 7 (20%) |  |
| no |  | 264 (72%) | 52 (75%) | 28 (80%) |  |
| *missing data* |  | 38 | *6* | *4* |  |
| ^1^Median (IQR); n (%) | | | | | |
| ^2^Kruskal-Wallis rank sum test; Pearson's Chi-squared test; Fisher's exact test | | | | | |

**Table S9. Clinical characteristics of main abn(7) subgroups**

The table shows the intensively treated patients grouped by the presence of complex karyotype and -7 or del(7q). Median groups are according to median values of Table 1.

| **Variable** | **n** | **-7/CK**,  n = 104^1^ | **-7/non-CK**,  n = 158^1^ | **del(7q)/CK**,  n = 52^1^ | **del(7q)/non-CK**,  n = 76^1^ | **p-value**^2^ |
| --- | --- | --- | --- | --- | --- | --- |
| **Age (median, IQR)** | 390 | 56 (46, 63) | 52 (39, 64) | 56 (47, 63) | 57 (44, 65) | 0.5 |
| **Sex** | 390 |  |  |  |  | 0.017 |
| female |  | 45 (43%) | 65 (41%) | 21 (40%) | 47 (62%) |  |
| male |  | 59 (57%) | 93 (59%) | 31 (60%) | 29 (38%) |  |
| **AML type** | 389 |  |  |  |  |  |
| *de novo AML* |  | 97 (93%) | 119 (76%) | 44 (85%) | 60 (79%) |  |
| secondary |  | 2 (1.9%) | 24 (15%) | 5 (9.6%) | 8 (11%) |  |
| therapy-related |  | 5 (4.8%) | 14 (8.9%) | 3 (5.8%) | 8 (11%) |  |
| *missing data* |  | *0* | *1* | *0* | *0* |  |
| **WBC** | 381 |  |  |  |  | 0.010 |
| < median |  | 61 (59%) | 64 (42%) | 31 (61%) | 32 (44%) |  |
| ≥ median |  | 42 (41%) | 90 (58%) | 20 (39%) | 41 (56%) |  |
| *missing data* |  | *1* | *4* | *1* | *3* |  |
| **Blast Count** | 361 |  |  |  |  | 0.028 |
| < median |  | 51 (54%) | 72 (49%) | 25 (49%) | 21 (31%) |  |
| ≥ median |  | 44 (46%) | 75 (51%) | 26 (51%) | 47 (69%) |  |
| *missing data* |  | *9* | *11* | *1* | *8* |  |
| **Platelets** | 349 |  |  |  |  | 0.024 |
| < median |  | 48 (50%) | 65 (46%) | 31 (67%) | 26 (39%) |  |
| ≥ median |  | 48 (50%) | 75 (54%) | 15 (33%) | 41 (61%) |  |
| *missing data* |  | *8* | *18* | *6* | *9* |  |
| **Complete remission** | 389 |  |  |  |  | 0.088 |
| no |  | 48 (46%) | 56 (36%) | 24 (46%) | 23 (30%) |  |
| yes |  | 56 (54%) | 101 (64%) | 28 (54%) | 53 (70%) |  |
| *missing data* |  | *0* | *1* | *0* | *0* |  |
| **Allo-HSCT in CR1** | 349 |  |  |  |  | 0.5 |
| yes |  | 27 (30%) | 52 (37%) | 13 (27%) | 24 (35%) |  |
| no |  | 64 (70%) | 89 (63%) | 35 (73%) | 45 (65%) |  |
| *missing data* |  | *13* | *17* | *4* | *7* |  |
| ^1^Median (IQR); n (%) | | | | | | |
| ^2^Kruskal-Wallis rank sum test; Pearson's Chi-squared test | | | | | | |

# Supplemental Datasets

### Dataset 1. Clinical data

### Dataset 2. SNP-Backbone information

### Dataset 3. SNVs from WES calls

### Dataset 4. SNVs from Targeted NGS

### Dataset 5. CNVs from Targeted NGS

### Dataset 6. cnLOH from Targeted NGS

### Dataset 7. CNVs from WES calls

# References

1. Shiraishi Y, Sato Y, Chiba K, et al. An empirical Bayesian framework for somatic mutation detection from cancer genome sequencing data. *Nucleic Acids Res*. Apr 2013;41(7):e89.

2. Christen F, Hoyer K, Yoshida K, et al. Genomic landscape and clonal evolution of acute myeloid leukemia with t(8;21): an international study on 331 patients. *Blood*. Mar 7 2019;133(10):1140-1151.

3. Arends CM, Galan-Sousa J, Hoyer K, et al. Hematopoietic lineage distribution and evolutionary dynamics of clonal hematopoiesis. *Leukemia*. Sep 2018;32(9):1908-1919.

4. Frick M, Chan W, Arends CM, et al. Role of Donor Clonal Hematopoiesis in Allogeneic Hematopoietic Stem-Cell Transplantation. *J Clin Oncol*. Feb 10 2019;37(5):375-385.

5. Damm F, Mylonas E, Cosson A, et al. Acquired initiating mutations in early hematopoietic cells of CLL patients. *Cancer Discov*. Sep 2014;4(9):1088-101.

6. Robinson JT, Thorvaldsdóttir H, Winckler W, et al. Integrative genomics viewer. *Nature Biotechnology*. 2011/01/01 2011;29(1):24-26.

7. Ley TJ, Miller C, Ding L, et al. Genomic and epigenomic landscapes of adult de novo acute myeloid leukemia. *N Engl J Med*. May 30 2013;368(22):2059-74.

8. Eisfeld A-K, Kohlschmidt J, Mrózek K, et al. Mutational Landscape and Gene Expression Patterns in Adult Acute Myeloid Leukemias with Monosomy 7 as a Sole Abnormality. *Cancer Research*. 2017;77(1):207-218.

9. Yoshida K, Gowers KHC, Lee-Six H, et al. Tobacco smoking and somatic mutations in human bronchial epithelium. *Nature*. 2020/02/01 2020;578(7794):266-272.

10. Alexandrov LB, Kim J, Haradhvala NJ, et al. The repertoire of mutational signatures in human cancer. *Nature*. 2020/02/01 2020;578(7793):94-101.

11. Machado HE, Mitchell E, Øbro NF, et al. Diverse mutational landscapes in human lymphocytes. *Nature*. 2022/08/01 2022;608(7924):724-732.

12. Van Loo P, Nordgard SH, Lingjærde OC, et al. Allele-specific copy number analysis of tumors. *Proc Natl Acad Sci U S A*. Sep 28 2010;107(39):16910-5.

13. Bernard E, Nannya Y, Hasserjian RP, et al. Implications of TP53 allelic state for genome stability, clinical presentation and outcomes in myelodysplastic syndromes. *Nature Medicine*. 2020/10/01 2020;26(10):1549-1556.

14. Hoyer K, Hablesreiter R, Inoue Y, et al. A genetically defined signature of responsiveness to erlotinib in early-stage pancreatic cancer patients: Results from the CONKO-005 trial. *EBioMedicine*. Apr 2021;66:103327.

15. Yannakou CK, Jones K, McBean M, et al. ASXL1 c.1934dup;p.Gly646Trpfs*12—a true somatic alteration requiring a new approach. *Blood Cancer Journal*. 2017/12/20 2017;7(12):656.

16. Schlenk RF, Döhner K, Krauter J, et al. Mutations and treatment outcome in cytogenetically normal acute myeloid leukemia. *N Engl J Med*. May 1 2008;358(18):1909-18.

17. Damm F, Heuser M, Morgan M, et al. Integrative prognostic risk score in acute myeloid leukemia with normal karyotype. *Blood*. 2011/04// 2011;117(17):4561-4568.

18. Gale RE, Green C, Allen C, et al. The impact of FLT3 internal tandem duplication mutant level, number, size, and interaction with NPM1 mutations in a large cohort of young adult patients with acute myeloid leukemia. *Blood*. Mar 1 2008;111(5):2776-84.

19. Riester M, Singh AP, Brannon AR, et al. PureCN: copy number calling and SNV classification using targeted short read sequencing. *Source Code for Biology and Medicine*. 2016/12/15 2016;11(1):13.

20. Klever MK, Sträng E, Hetzel S, et al. AML with complex karyotype: extreme genomic complexity revealed by combined long-read sequencing and Hi-C technology. *Blood Adv*. Nov 14 2023;7(21):6520-6531.

21. Scheinin I, Sie D, Bengtsson H, et al. DNA copy number analysis of fresh and formalin-fixed specimens by shallow whole-genome sequencing with identification and exclusion of problematic regions in the genome assembly. *Genome Res*. Dec 2014;24(12):2022-32.

22. Noerenberg D, Briest F, Hennch C, et al. Genetic Characterization of Primary Mediastinal B-Cell Lymphoma: Pathogenesis and Patient Outcomes. *J Clin Oncol*. Feb 1 2024;42(4):452-466.

23. Niknafs N, Balan A, Cherry C, et al. Persistent mutation burden drives sustained anti-tumor immune responses. *Nature Medicine*. 2023/02/01 2023;29(2):440-449.

24. Canisius S, Martens JWM, Wessels LFA. A novel independence test for somatic alterations in cancer shows that biology drives mutual exclusivity but chance explains most co-occurrence. *Genome Biology*. 2016/12/16 2016;17(1):261.

25. Harrison E; Drake T; Pius R. finalfit: Quickly Create Elegant Regression Results Tables and Plots when Modelling. Version R package version 1.0.7. 2023.

26. Arber DA, Orazi A, Hasserjian RP, et al. International Consensus Classification of Myeloid Neoplasms and Acute Leukemias: integrating morphologic, clinical, and genomic data. *Blood*. Sep 15 2022;140(11):1200-1228.
